# Supplementary material for: Asymmetric rotaxanes as dual-modality supramolecular imaging agents for targeting cancer biomarkers
Source: Commun Chem. 2023 Jun 1;6:107. doi: 10.1038/s42004-023-00906-5 (PMC10235045; doi:10.1038/s42004-023-00906-5)
Supplement: Supplementary file 2 — Supplementary Information [file 42004_2023_906_MOESM2_ESM.pdf]

Supporting Information

Asymmetric rotaxanes as dual-modality supramolecular imaging agents for targeting cancer biomarkers

Faustine d'Orchymont and Jason P. Holland*

University of Zurich, Department of Chemistry, Winterthurerstrasse 190, CH-8057, Zurich, Switzerland

* **Corresponding Author:**

Prof. Dr Jason P. Holland

ORCID: orcid.org/0000-0002-0066-219X

Tel: +41-44-63-53990

E-mail: jason.holland@chem.uzh.ch

Website: www.hollandlab.org

First Author:

Dr Faustine d'Orchymont

ORCID: orcid.org/0000-0002-3726-1648

E-mail: faustine.dorchymont@chem.uzh.ch

Table of Contents

Supplementary Note 1 5

Synthesis and Characterisation 5

General procedure A: Preparation of ^{nat}Ga complexes 5

Synthesis of the fluorescein-alkyne dissymmetrical guest with an ethylenediamine group 1 5

Scheme S1. Synthesis of 1: (a) HCl, THF/H_2O, 23 °C, 5 min, (b) Boc-1,2-diaminoethane, $Cl(CH_2)_2Cl$, 23 °C, 1 h, (c) $NaBH(OAc)_3$, 50 °C, 2 h, 45%, (d) TFA, CH_2Cl_2, 0 to 23 °C, 2 h, 99%, (e) NHS, EDC·HCl, DMF, 23 °C, 2 h, (f) 12, Et_3N, DMF, 23°C, 12 h, 45%. 5

Figure S1. ^{1}H NMR of compound 11 (MeOD, 500 MHz). 6

Figure S2. $^{13}C\{^1H\}$ NMR of compound 11 (MeOD, 126 MHz). 7

Figure S3. HSQC of compound 11 (MeOD). 7

Figure S4. HMBC of compound 11 (MeOD). 8

Figure S5. ^{1}H NMR of compound 12 (MeOD, 500 MHz). 9

Figure S6. $^{13}C\{^1H\}$ NMR of compound 12 (MeOD, 126 MHz). 9

Figure S7. HSQC of compound 12 (MeOD). 10

Figure S8. HMBC of compound 12 (MeOD). 10

Figure S9. Reverse-phase analytical HPLC chromatogram of compound 1, λ= 254 nm. 11

Figure S10. ^{1}H NMR of compound 1 (MeOD, 500 MHz). 12

Figure S11. $^{13}C\{^1H\}$ NMR of compound 1 (MeOD, 126 MHz). 12

Figure S12. HSQC of compound 1 (MeOD). 13

Figure S13. HMBC of compound 1 (MeOD). 13

^{1}H NMR titration for 1⊃β-CD 14

Figure S14. Selected regions of the ^{1}H NMR spectra of (A) 1 when [1] increases, and (B) β-CD (except H_1) when [β-CD] decreases but [1] + [β-CD] = 2.5 mM. 14

Figure S15. Chemical shifts variation of selected proton resonance peaks for (A) β-CD, and (B) and (C) 1, as a function of substrate concentration. 15

Figure S16. Job's plots corresponding to the induced chemical shift variation of selected resonance peaks associated with (A) β-CD, and (B) and (C) 1 protons during the formation of the 1⊃β-CD inclusion complex. 15

Figure S17. (A) Benesi-Hildebrand, (B) Scott, and (C) Scatchard plots for the 1⊃β-CD inclusion complex. 15

Synthesis of the [3]pseudorotaxane 4 and the metallo[3]rotaxane ^{nat}Ga-4 16

Scheme S2. Synthesis of [3]semirotaxane 4: (a) H_2O, 70 °C, 1 min, 72%. 16

Figure S18. Reverse-phase analytical HPLC chromatogram of complex 4, λ= 254 nm. 17

Figure S19. HRMS (ESI+) spectrum of compound 4 17

Figure S20. ^{1}H NMR of compound 4 (D_2O, 500 MHz). 18

Figure S21. $^{13}C\{^1H\}$ NMR of compound 4 (D_2O, 126 MHz). 18

Figure S22. DEPT-135 of compound 4 (D_2O). 19

Figure S23. DEPT-90 of compound 4 (D_2O). 19

Figure S24. HSQC of compound 4 (D_2O). 20

Figure S25. HMBC of compound 4 (D_2O). 20

Figure S26. ROESY of compound 4 (D_2O). 21

Figure S27. Reverse-phase analytical HPLC chromatogram of complex ^{nat}Ga-4, λ= 254 nm. 21
Figure S28. HRMS (ESI+) spectrum of compound ^{nat}Ga-4. 22
Radiosynthesis of [^{68}Ga]Ga-4 22
Synthesis of the fluorescein-azido compound 5 23
Scheme S3. Synthesis of 5: (a) NHS, EDC·HCl, DMF, 23 °C, 2 h; (b) Boc-1,2-diaminoethane, Et_3N, DMF, 23 °C, 12 h; (c) TFA, CH_2Cl_2, 0 to 23 °C, 2 h, 29%; (d) NHS, EDC·HCl, DMF, 23 °C, 2 h; (e) 14, Et_3N, DMF, 23 °C, 12 h, 94%. 23
Figure S29. ^{1}H NMR of compound 14 (D_2O, 500 MHz). 24
Figure S30. ^{13}C{^{1}H} NMR of compound 14 (D_2O, 126 MHz). 24
Figure S31. HSQC of compound 14 (D_2O). 25
Figure S32. Reverse-phase analytical HPLC chromatogram of compound 5, λ= 254 nm. 26
Figure S33. ^{1}H NMR of compound 5 (MeOD, 500 MHz). 26
Figure S34. ^{13}C{^{1}H} NMR of compound 5 (MeOD, 126 MHz). 27
Figure S35. HSQC of compound 5 (MeOD). 27
Synthesis of [4]semirotaxane 6 28
Scheme S4. Synthesis of [4]semirotaxane 6: (a) H_2O, 70 °C, 1 min, 16%. 28
Figure S36. Reverse-phase analytical HPLC chromatogram of [4]semirotaxane 6, λ = 254 nm. 29
Figure S37. HRMS (ESI+) spectrum of [4]semirotaxane 6. 29
Figure S38. ^{1}H NMR of [4]semirotaxane 6 (D_2O, 500 MHz). 30
Figure S39. ^{13}C{^{1}H} NMR of [4]semirotaxane 6 (D_2O, 126 MHz). 31
Figure S40. Reverse-phase analytical HPLC chromatogram of complex ^{nat}Ga-6, λ = 254 nm. 32
Figure S41. HRMS (ESI+) spectrum of complex ^{nat}Ga-6. 32
Radiosynthesis of [^{68}Ga]Ga-6 33
Synthesis of [4]rotaxane 7 34
Scheme S5. Synthesis of [4]rotaxane 7: (a) H_2O, 70 °C, 1 min, 65%. 34
Figure S42. Reverse-phase analytical HPLC chromatogram of [4]rotaxane 7, λ = 254 nm. 35
Figure S43. HRMS (ESI+) spectrum of [4]rotaxane 7. 35
Figure S44. ^{1}H NMR of [4]rotaxane 7 (D_2O, 500 MHz). 36
Figure S45. ^{13}C{^{1}H} NMR of [4]rotaxane 7 (D_2O, 126 MHz). 37
Figure S46. DEPT-135 of [4]rotaxane 7 (D_2O). 38
Figure S47. DEPT-90 of [4]rotaxane 7 (D_2O). 38
Figure S48. HSQC of [4]rotaxane 7 (D_2O). 39
Figure S49. HMBC of [4]rotaxane 7 (D_2O). 39
Figure S50. ROESY of [4]rotaxane 7 (D_2O). 40
Electronic absorption spectroscopy and determination of the molar absorption coefficient for [4]rotaxane 7 40
Figure S51. Electronic absorption spectroscopy to determine molar absorption coefficients of [4]rotaxane 7. 40
Table S1. Molar absorption coefficients of [4]rotaxane 7. 41
Figure S52. Excitation and fluorescence emission spectra for the [4]rotaxane 7. 41
Synthesis of the PSMA targeted [3]semirotaxane 9 and metallo[3]rotaxane ^{nat}Ga-9 42

Figure S53. Reverse-phase analytical HPLC chromatogram of [3]semirotaxane 9, λ= 254 nm. 42
Figure S54. HRMS (ESI+) spectrum of [3]semirotaxane 9. 43
Figure S55. ^{1}H NMR of [3]semirotaxane 9 (D_2O, 500 MHz). 43
Figure S56. $^{13}C\{^{1}H\}$ NMR of [3]semirotaxane 9 (D_2O, 126 MHz). 44
Figure S57. DEPT-135 of [3]semirotaxane 9 (D_2O) 44
Figure S58. DEPT-90 of [3]semirotaxane 9 (D_2O) 45
Figure S59. HSQC of [3]semirotaxane 9 (D_2O). 45
Figure S60. HMBC of [3]semirotaxane 9 (D_2O). 46
Figure S61. ROESY of [3]semirotaxane 9 (D_2O). 46
Figure S62. Reverse-phase analytical HPLC chromatogram of complex ^{nat}Ga-9, λ= 254 nm. 47
Figure S63. HRMS (ESI+) spectrum of complex ^{nat}Ga-9. 47
Radiosynthesis of [^{68}Ga]Ga-9 48
Figure S64. (A) Radio-iTLC chromatograms of 9 in citrate buffer and associated control; (B) Analytical HPLC chromatograms recorded at 254 nm of the purified semirotaxane 9, and the corresponding ^{nat}Ga-9 and [^{68}Ga]Ga-9 [3]rotaxanes. 48
Quantum yield of compound ^{nat}Ga-9 48
Equation S1. Quantum yield calculated using the comparative method of Williams et al.,[2] which involves the use of the well characterised standard fluorescein sample with a known quantum yield $\Phi_{Fluorescein}$. 48
Electronic absorption spectroscopy and determination of the molar absorption coefficient for compound ^{nat}Ga-9 49
Figure S65. Electronic absorption spectroscopy to determine molar absorption coefficients of compound ^{nat}Ga-9. 49
Table S2. Molar absorption coefficients of compound ^{nat}Ga-9. 49
Figure S66. Electronic excitation and fluorescence emission spectra of compound ^{nat}Ga-9. 49
Stability studies for 4, 9, ^{nat}Ga-4, ^{nat}Ga-9, [^{68}Ga]Ga-4and [^{68}Ga]Ga-9 50
Figure S67. HPLC chromatograms recorded at 254 nm at various time points for: (A) 4, (B) ^{nat}Ga-4, (C) 9, (D) ^{nat}Ga-9, and (E) the corresponding plot obtained from integration of the HPLC data showing the relative stability of the compounds versus time. 50
Table S3. Percentage RCP of [^{68}Ga]Ga-4 and [^{68}Ga]Ga-9 determined from radio-iTLC following incubation with PBS up to 2 h at 37 °C. 51
Table S4. Percentage RCP of [^{68}Ga]Ga-4 and [^{68}Ga]Ga-9 determined from radio-SEC-HPLC following incubation with human serum for up to 2 h at 37 °C. 51
Cell binding assays with [^{68}Ga]Ga-4 and [^{68}Ga]Ga-9 52
Figure S68. Chemical structure of the PSMA binding ligand used in cellular blocking experiments. 52
References 52

Supplementary Note 1

Synthesis and Characterisation

Compounds **3**, **8**, **10**, and **13** were synthesised following procedures described elsewhere.[1]

General procedure A: Preparation of natGa complexes

To a suspension of the compound containing a desmetallated chelate in H_2O (5 mL) a solution of $Ga(NO_3)_3$ (2 equiv.) in H_2O (0.5 mL) was added dropwise. The resulting clear, colourless solution was stirred at 23 °C for 2 h. After evaporation of the solvent under reduced pressure, the crude mixture was purified by semi-preparative HPLC using the method previously described.[1] After lyophilisation, the ^{nat}Ga, complexes were obtained as a slightly yellow solids. The purity of the products was measured by analytical HPLC and the complex identities were confirmed by HRMS (ESI) spectrometry.

Synthesis of the fluorescein-alkyne dissymmetrical guest with an ethylenediamine group **1**

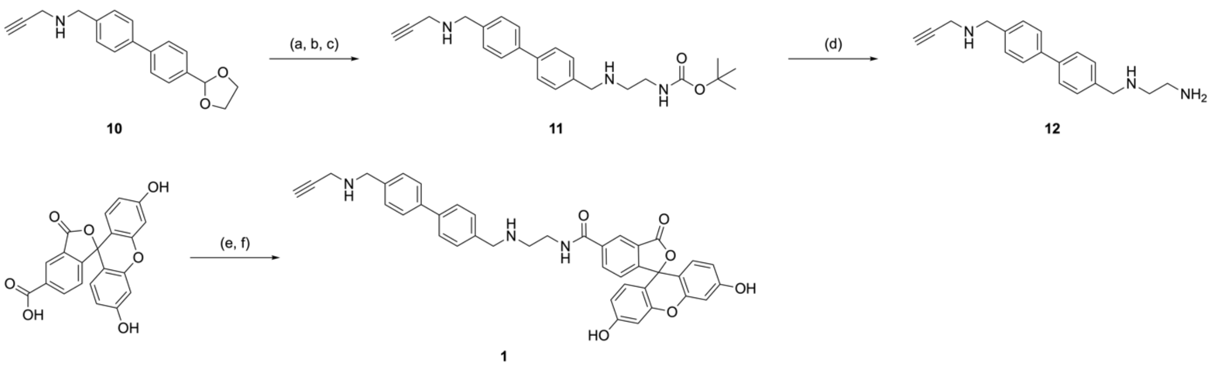

Scheme S1. Synthesis of **1**: (a) HCl, THF/H_2O, 23 °C, 5 min, (b) Boc-1,2-diaminoethane, $Cl(CH_2)_2Cl$, 23 °C, 1 h, (c) $NaBH(OAc)_3$, 50 °C, 2 h, 45%, (d) TFA, CH_2Cl_2, 0 to 23 °C, 2 h, 99%, (e) NHS, EDC·HCl, DMF, 23 °C, 2 h, (f) **12**, Et_3N, DMF, 23°C, 12 h, 45%.

Compound 11

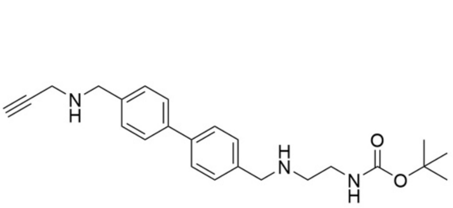

Compound **10** (20 mg, 0.07 mmol, 1 equiv.) was dissolved in a solution of HCl/H_2O/THF (1:6:7, 2 mL). The solution was stirred for 5 min at 23 °C. $NaHCO_3$ was then added to adjust to pH7. EtOAc was added and the organic phase was washed with brine. The solvent was removed under reduced pressure to afford the acetal deprotected intermediate as a brown residue which was used for the next step without further purification. To a solution of this intermediate (17 mg, 0.07 mmol, 1 equiv.) in 1,2-dichloroethane (5 mL) was added *N*-Boc-ethylenediamine (13 mg, 0.08 mmol, 1.2 equiv.). The resulting mixture was stirred at 23 °C for 1 h. Then $NaBH(OAc)_3$ (29 mg, 0.14 mmol, 2 equiv.) was added. The resulting

mixture was stirred at 23 °C for 1 h. Then the reaction was quenched by the addition of MeOH. The solvent was removed under reduced pressure. The crude residue was dissolved in CH_2Cl_2, washed with an aqueous sat. solution of $NaHCO_3$, and brine, and concentrated under reduced pressure. The crude product was purified by silica gel column chromatography (CH_2Cl_2/MeOH 9:1) to afford **11** (12 mg, 45% yield) as a slightly yellow oil. R_f (CH_2Cl_2/MeOH 9:1) 0.21; ^{1}H NMR (500 MHz, MeOD) δ = 7.58−7.62 (*m*, 4H), 7.39−7.44 (*m*, 4H), 3.88 (*s*, 2H), 3.82 (*s*, 2H), 3.39 (*d*, *J* = 2.5 Hz, 2H), 3.22 (*t*, *J* = 6.1 Hz, 2H), 2.72 (*t*, *J* = 6.2 Hz, 2H), 2.67 (*t*, *J* = 2.5 Hz, 1H), 1.43 ppm (*s*, 9H); $^{13}C\{^1H\}$ NMR (126 MHz, MeOD) δ = 158.6, 141.2, 141.2, 139.3, 139.1, 130.3, 130.1, 128.0, 128.0, 81.9, 80.2, 73.5, 53.7, 52.4, 49.4, 40.6, 37.5, 28.7 ppm; HRMS (ESI) *m/z* calcd for $C_{24}H_{32}N_3O_2$ $[M+H]^+$ 394.2489, found 394.2492 (100).

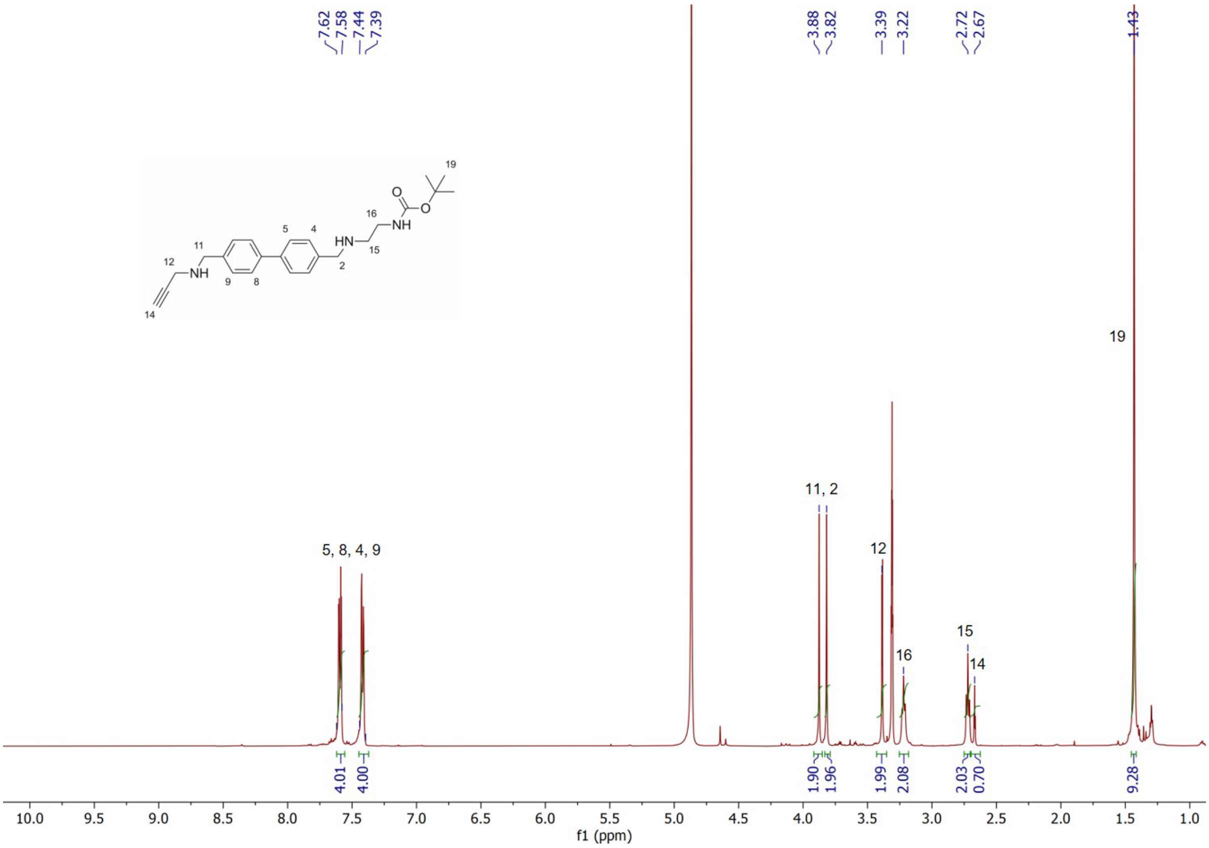

Figure S1. ^{1}H NMR of compound **11** (MeOD, 500 MHz).

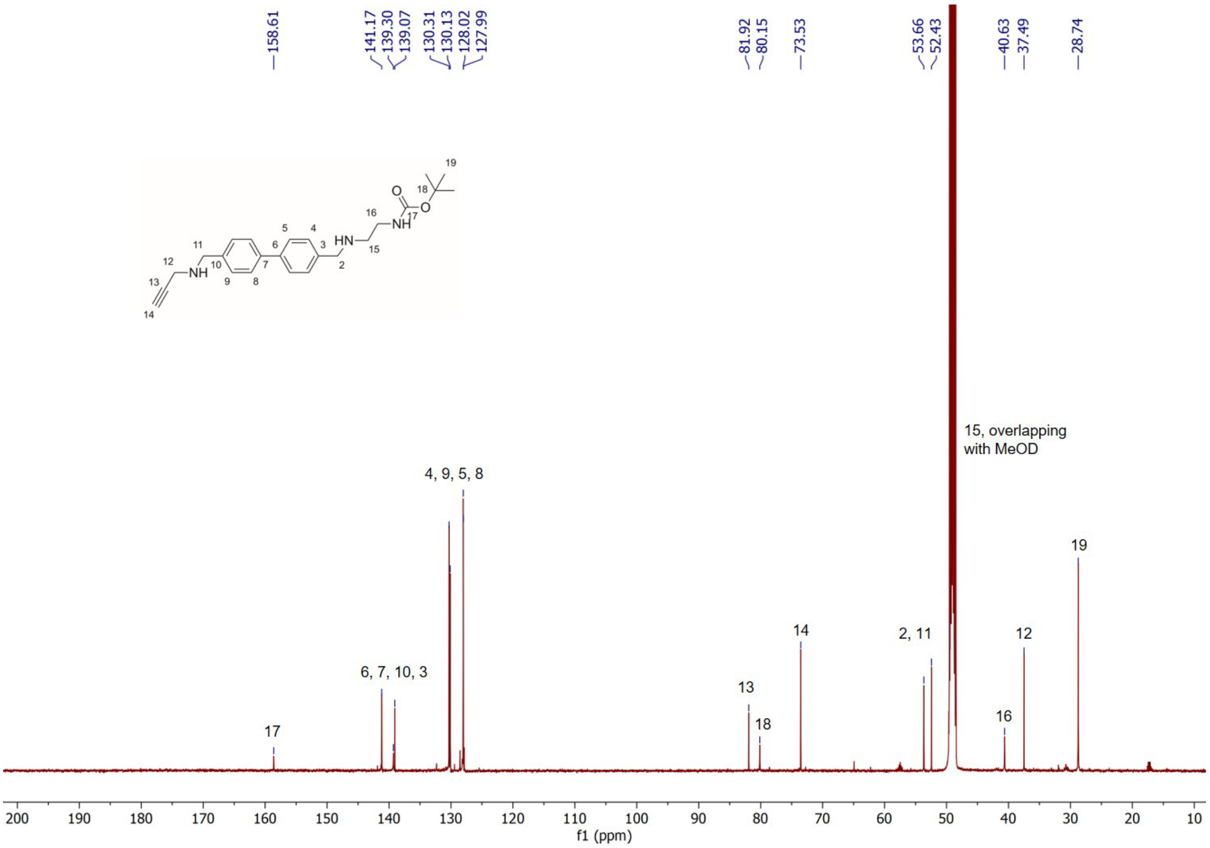

Figure S2. $^{13}C\{^{1}H\}$ NMR of compound **11** (MeOD, 126 MHz).

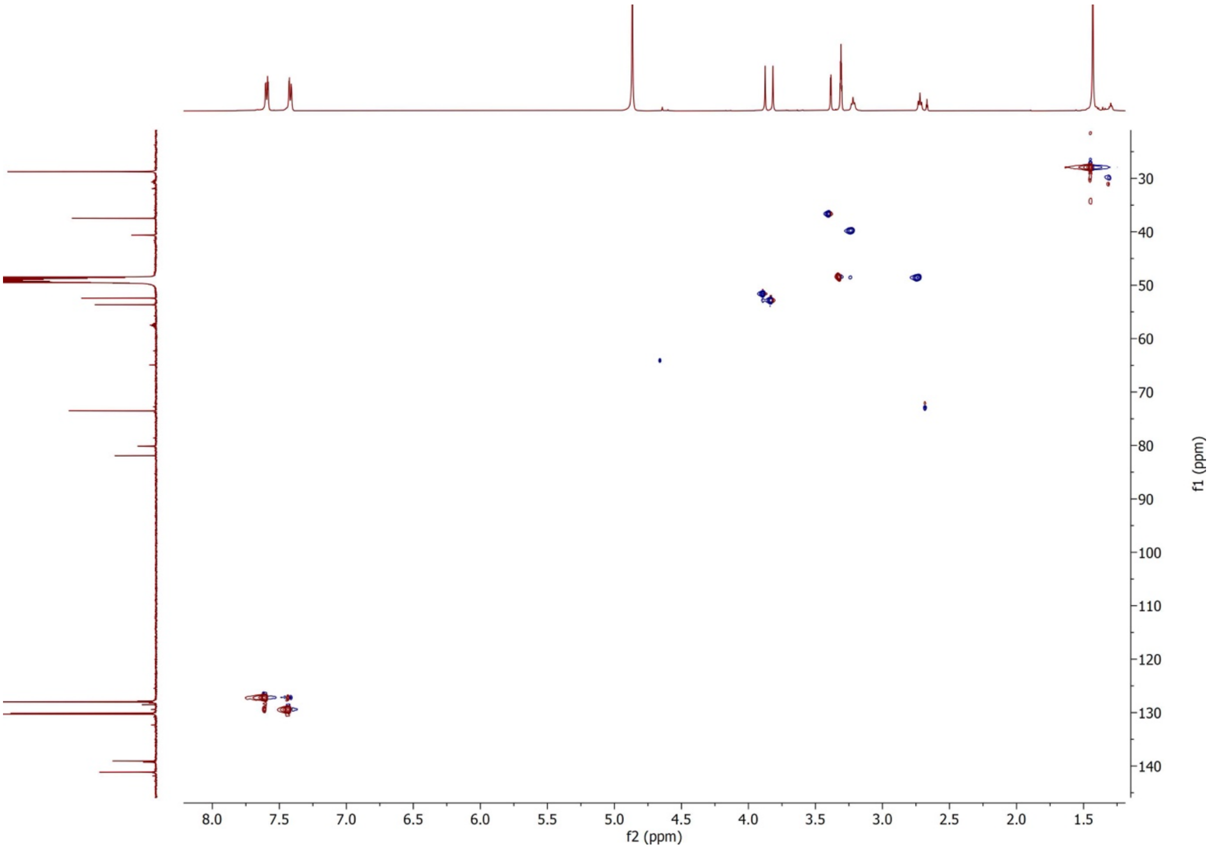

Figure S3. HSQC of compound **11** (MeOD).

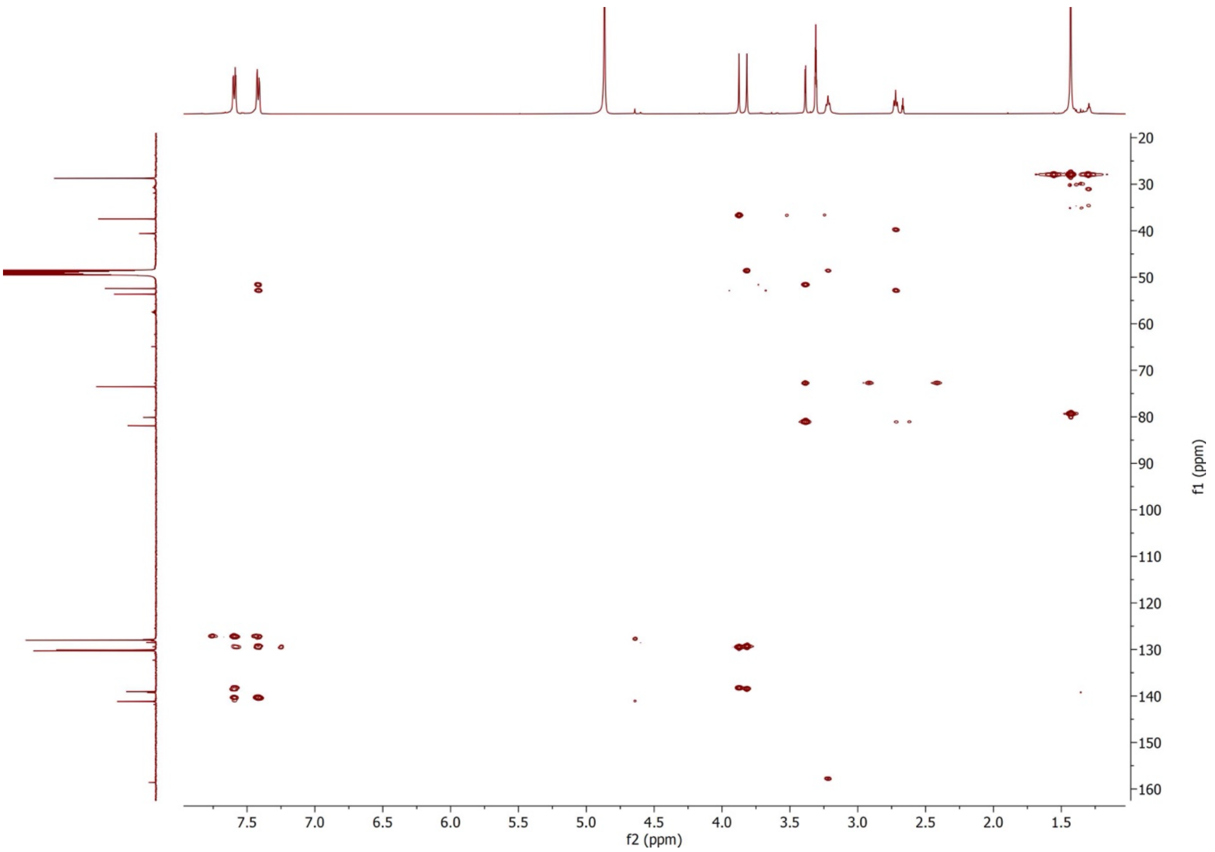

Figure S4. HMBC of compound **11** (MeOD).

Compound 12

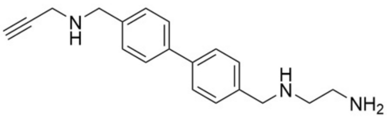

To an ice-cooled solution of **11** (11 mg, 0.03 mmol, 1 equiv.) in CH_2Cl_2 (1 mL) was added TFA (1 mL). The resulting mixture was warmed up to 23 °C, protected from light, and stirred at 23 °C for 2 h. The solvent was then removed under reduced pressure and the crude residue was washed with Et_2O, separated from the supernatant by centrifugation to afford **12** (8 mg, 99% yield) as a brown oil; ^{1}H NMR (500 MHz, MeOD) δ = 7.76 (*d*, *J* =8.2 Hz, 2H), 7.75 (*d*, *J* =8.2 Hz, 2H), 7.61 (*d*, *J* =8.3 Hz, 2H), 7.59 (*d*, *J* =8.3 Hz, 2H), 4.34 (*s*, 2H), 4.31 (*s*, 2H), 3.98 (*d*, *J* =2.4 Hz, 2H), 3.37−3.41 (*m*, 2H), 3.32−3.37 (*m*, 2H), 3.31 ppm (*t*, 1H, overlapping with MeOD); ^{13}C{^{1}H} NMR (126 MHz, MeOD) δ =163.2 (*q*, ^2J = 34.4 Hz), 142.9, 142.5, 132.6, 131.8, 131.6, 131.5, 128.9, 128.8, , 118.2 (*q*, ^1J = 291.2 Hz), 79.6, 74.5, 52.4, 50.9, 45.4, 37.1, 36.9 ppm; HRMS (ESI) *m/z* calcd for $C_{19}H_{24}N_3$ $[M+H]^+$ 294.1965, found 294.1963 (100).

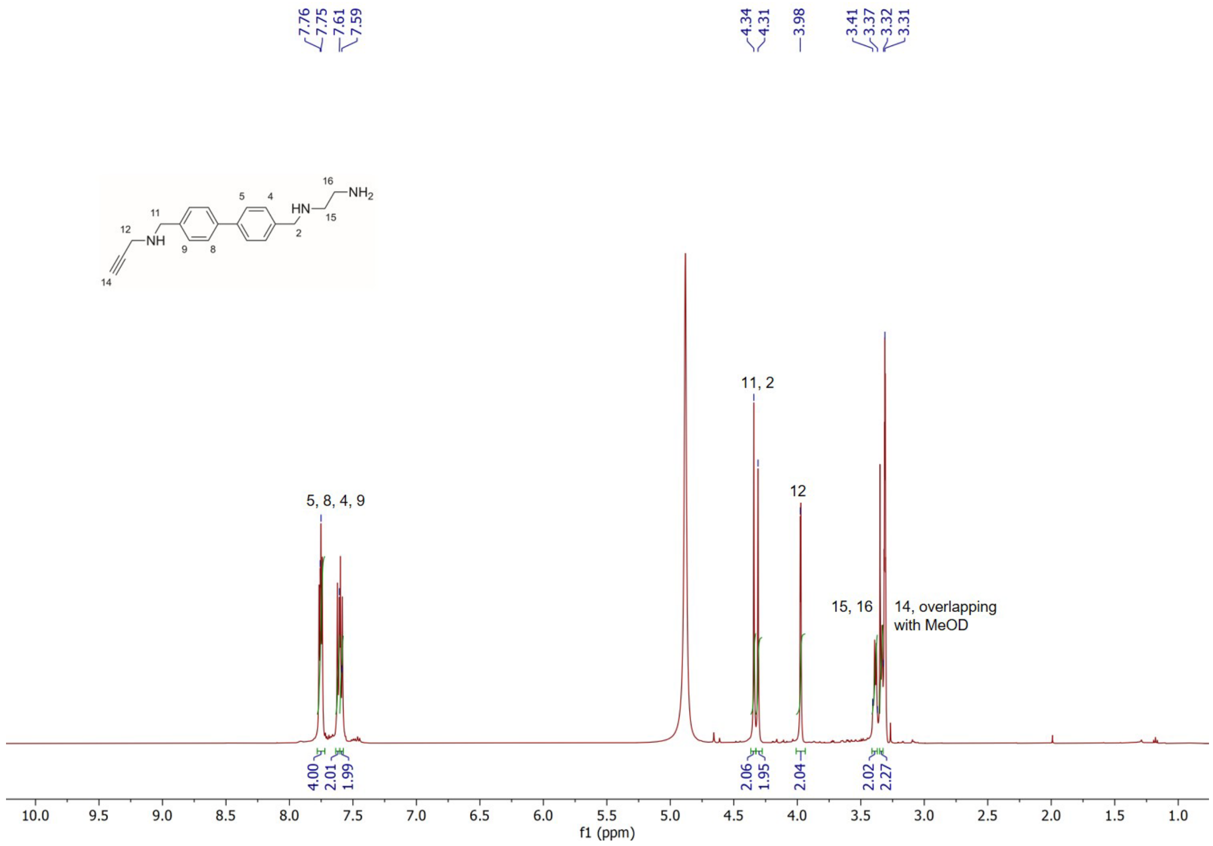

Figure S5. ^{1}H NMR of compound **12** (MeOD, 500 MHz).

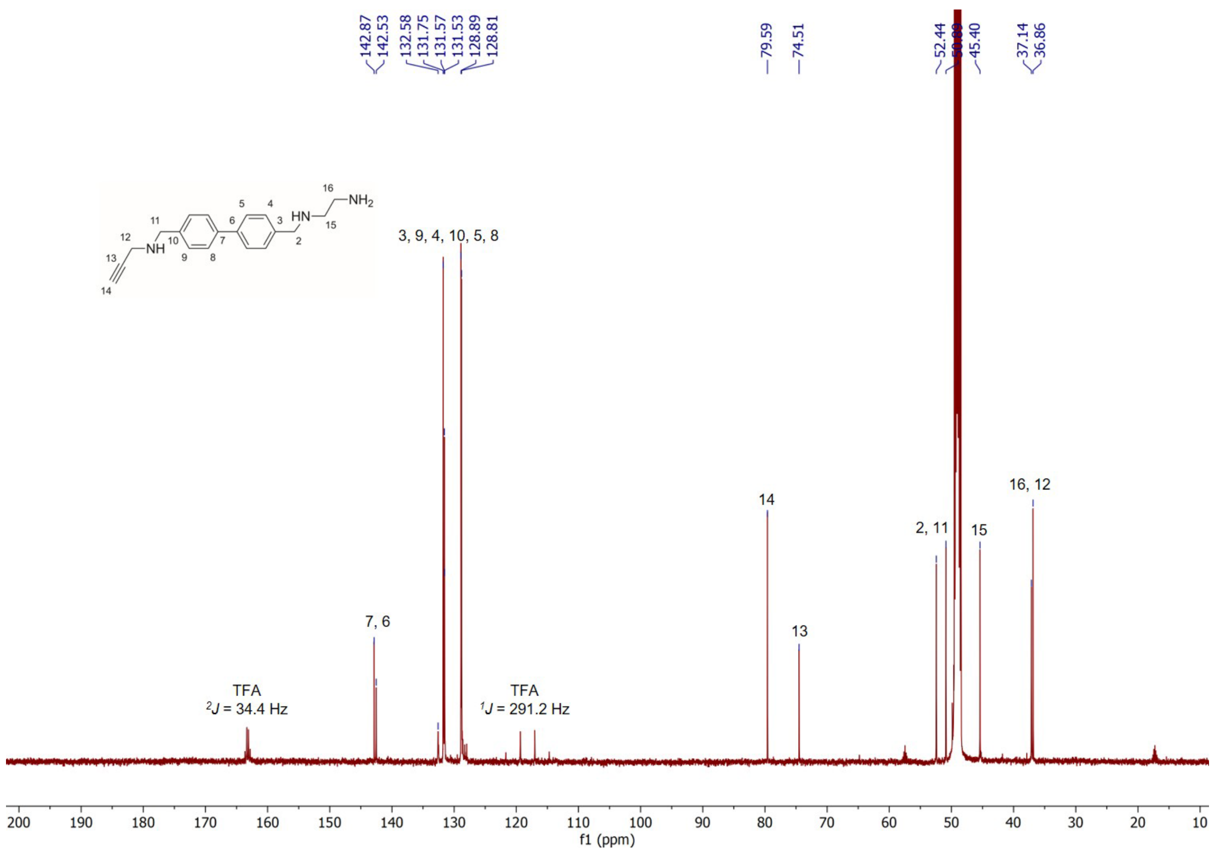

Figure S6. $^{13}C\{^{1}H\}$ NMR of compound **12** (MeOD, 126 MHz).

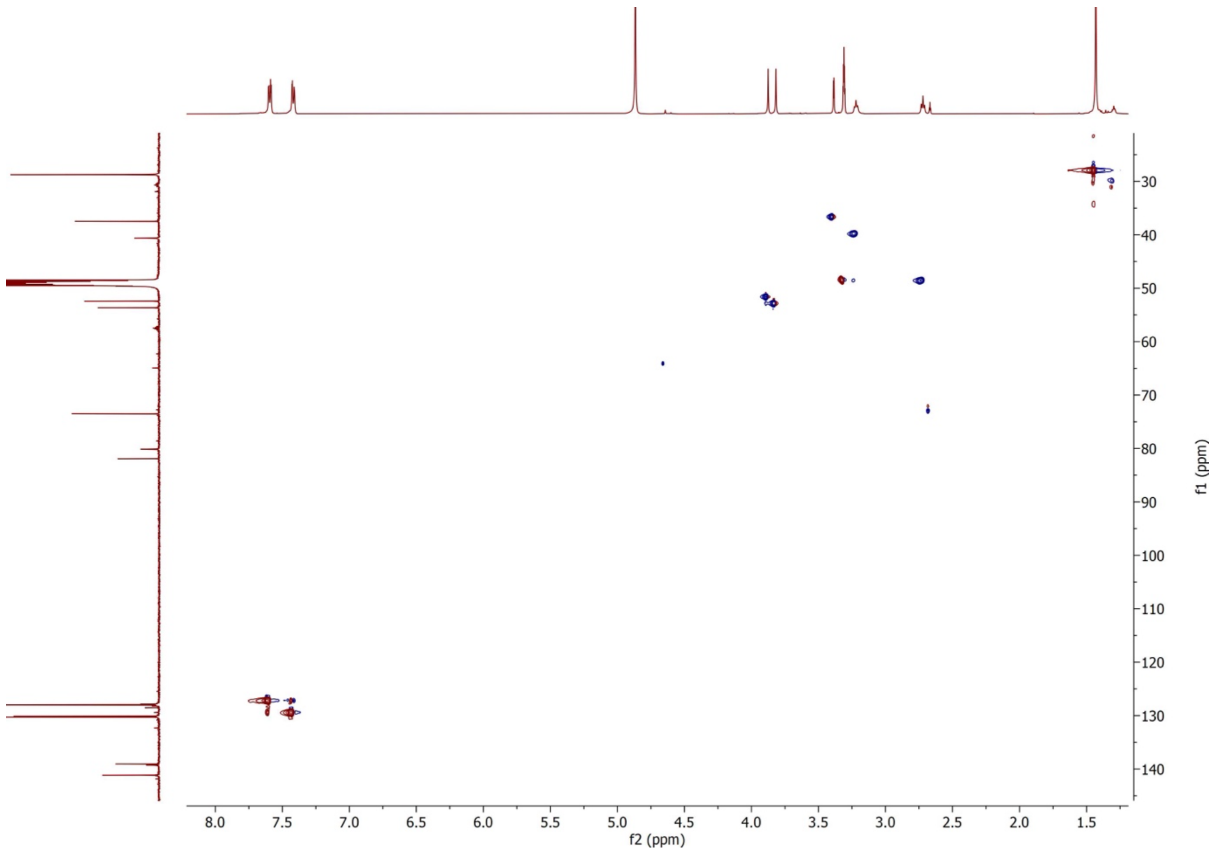

Figure S7. HSQC of compound **12** (MeOD).

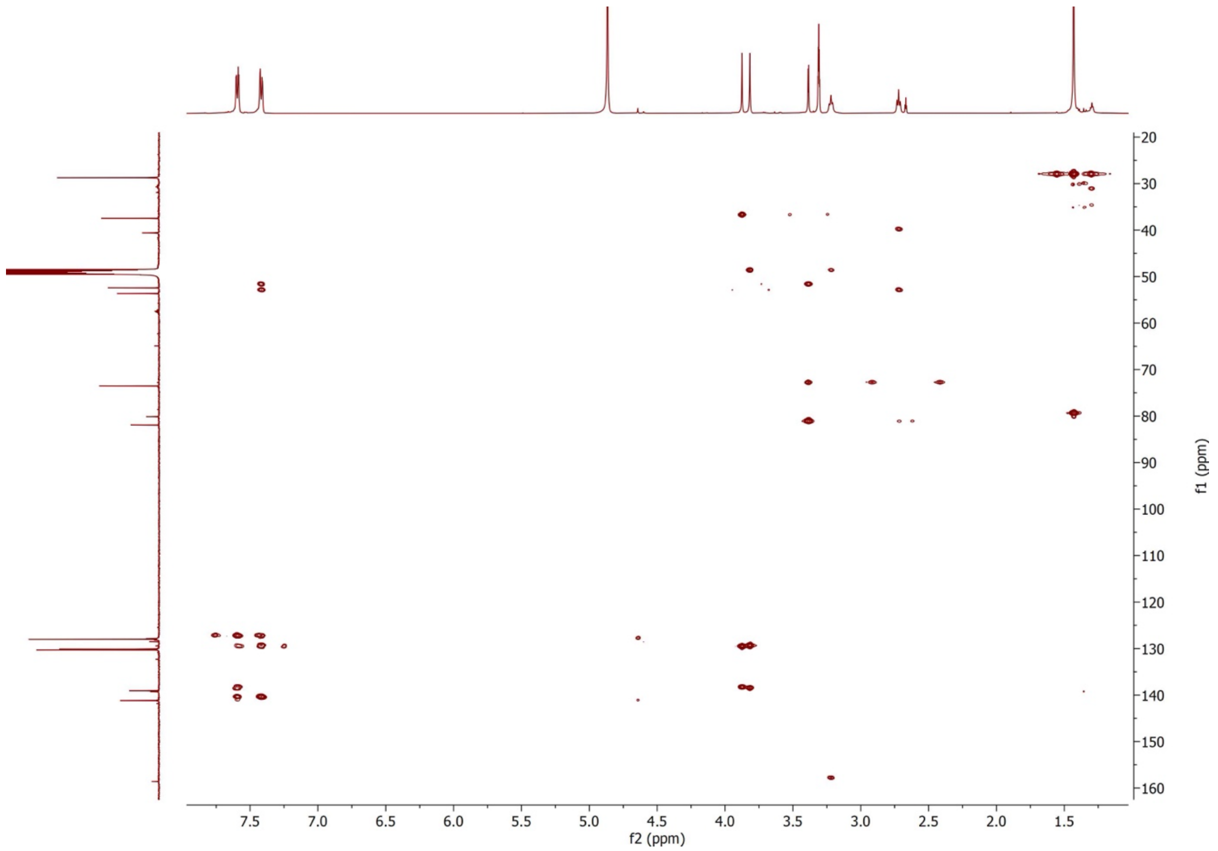

Figure S8. HMBC of compound **12** (MeOD).

Compound 1

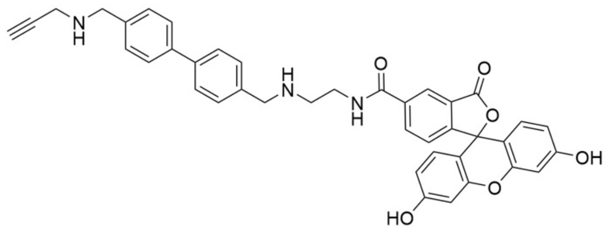

To a solution of 5-carboxyfluorescein (7 mg, 0.02 mmol, 1.1 equiv.) in DMF (1 mL) were added EDC·HCl (4 mg, 0.02 mmol, 1.1 equiv.) and *N*-hydroxysuccinimide (2 mg, 0.02 mmol, 1.1 equiv.). The resulting solution was stirred at 23 °C for 2 h. Then **12** (5 mg, 0.02 mmol, 1 equiv.) and Et_3N (0.003 mL, 0.02 mmol, 1.2 equiv.) were added and the reaction mixture was stirred for 12 h at 23 °C. Then DMF was evaporated under reduced pressure. The crude was purified by using semi-preparative HPLC at a flow rate of 7 mL min^{-1} with a linear gradient of A (MeOH, Sigma-Aldrich, HPLC grade) and B (distilled water containing 0.1% TFA): t = 0 min A 5% + B 95%, t = 30 min A 100% + B 0%. The sample was lyophilised to afford **1** (5 mg, 45% yield) as a bright yellow residue; The product was estimated by analytical HPLC to have a purity >95%; ^{1}H NMR (500 MHz, MeOD) δ = 8.49 (*d*, *J* = 1.5 Hz, 1H), 8.25 (*dd*, *J* = 1.5, 8.1 Hz, 1H), 7.77 (*d*, *J* = 8.1 Hz, 4H), 7.64 (*d*, *J* = 8.3 Hz, 2H), 7.58 (*d*, *J* = 8.3 Hz, 2H), 7.33 (*d*, *J* = 8.1 Hz, 1H), 6.74 (*d*, *J* = 2.3 Hz, 2H), 6.60 (*d*, *J* = 8.8 Hz, 2H), 6.56 (*dd*, *J* = 2.3, 8.8 Hz, 2H), 4.37 (*s*, 2H), 4.34 (*s*, 2H), 3.97 (*d*, *J* = 2.5 Hz, 2H), 3.82 (*t*, *J* = 5.7 Hz, 2H), 3.39 (*t*, *J* = 5.7 Hz, 2H), 3.31 ppm (*t*, 1H, overlapping with MeOD); ^{13}C{^{1}H} NMR (126 MHz, MeOD) δ = 170.3, 169.6, 162.1, 161.6 (*q*, ^{2}J = 37.6 Hz), 154.4, 154.4, 142.8, 142.6, 137.0, 135.5, 132.1, 131.8, 131.7, 131.5, 130.3, 128.9, 128.9, 128.9, 126.1, 125.5, 117.5 (*q*, ^{1}J = 288.2 Hz), 114.1, 111.1, 103.6, 79.7, 74.4, 52.0, 50.9, 48.8, 37.8, 36.9 ppm; HRMS (ESI) *m/z* calcd for $C_{40}H_{35}N_3O_6$ $[M+2H]^{2+}$ 326.6257, found 326.6257 (100).

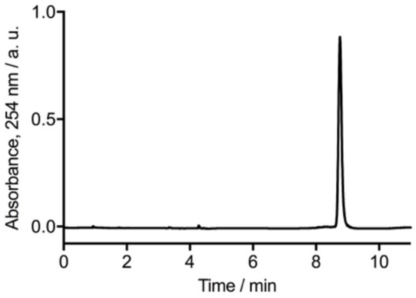

Figure S9. Reverse-phase analytical HPLC chromatogram of compound **1**, λ= 254 nm.

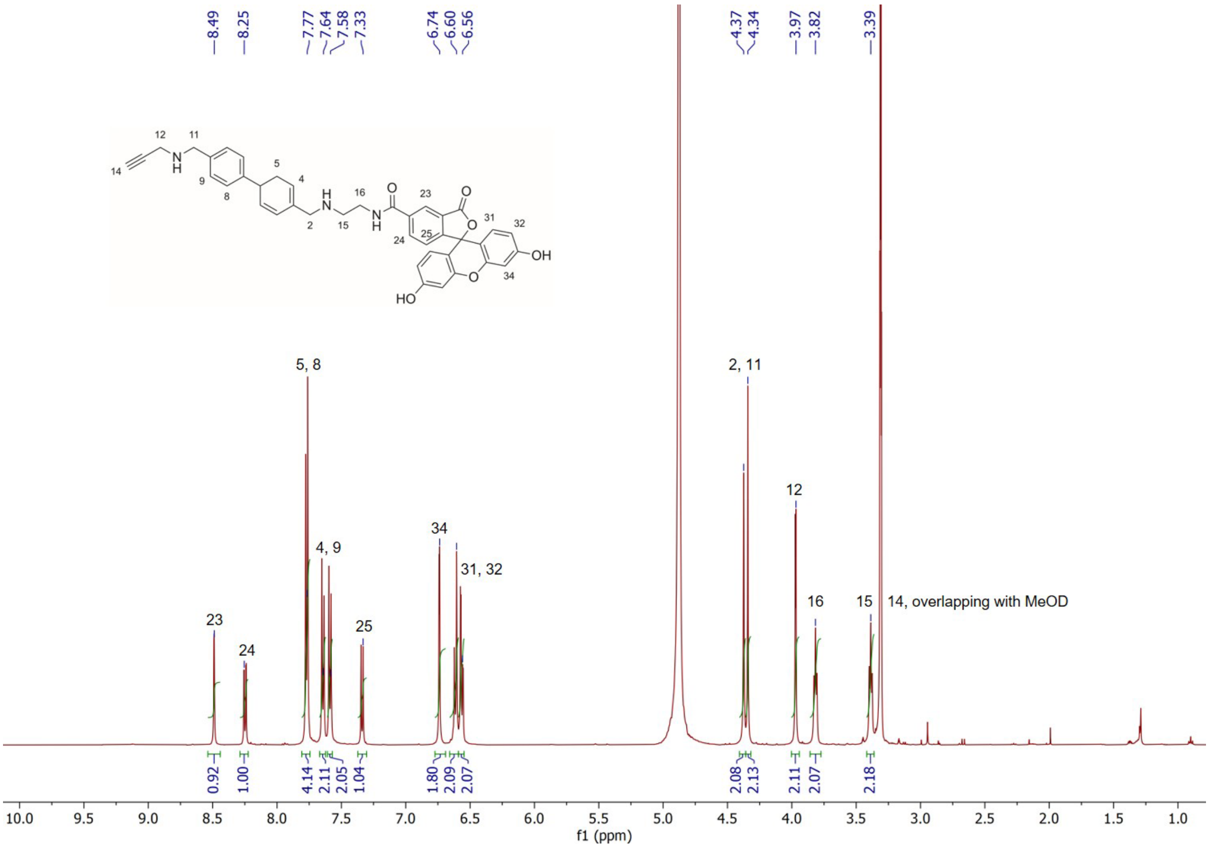

Figure S10. ^{1}H NMR of compound **1** (MeOD, 500 MHz).

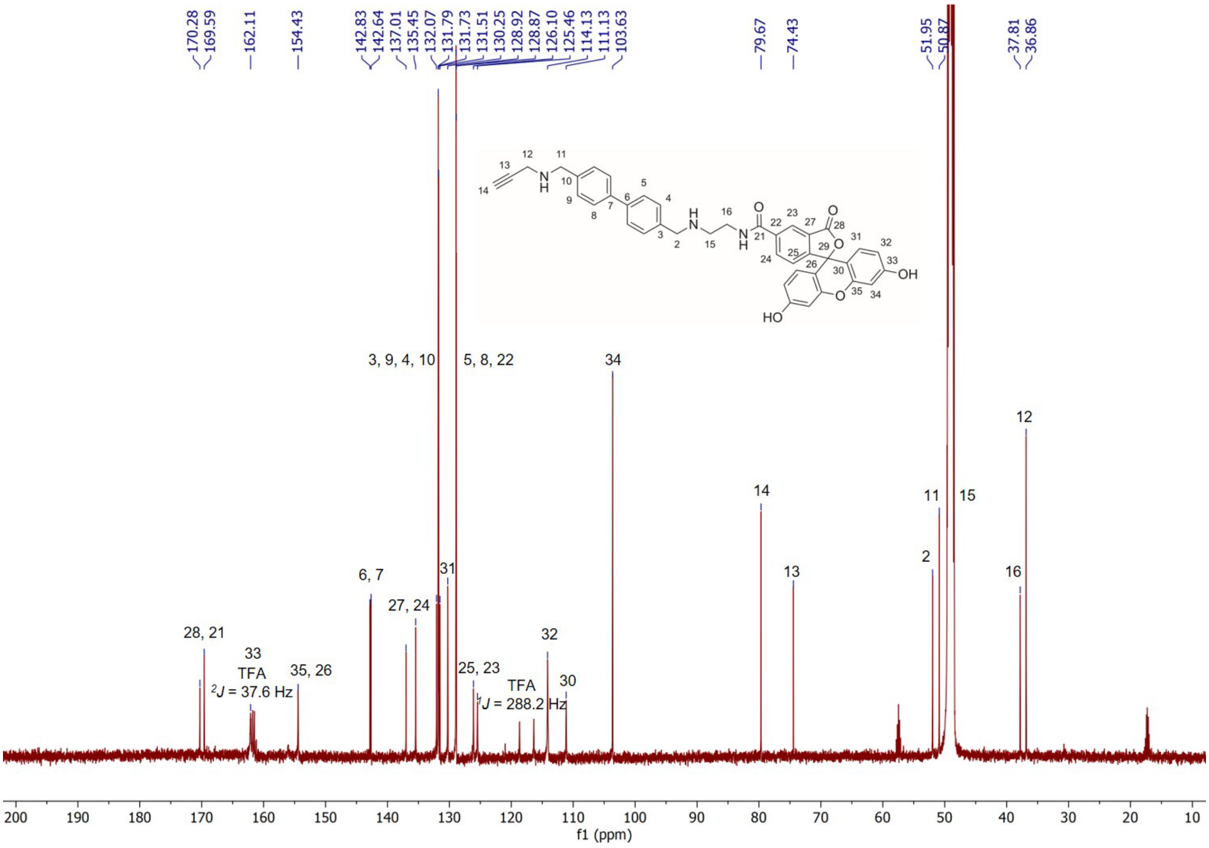

Figure S11. $^{13}C\{^{1}H\}$ NMR of compound **1** (MeOD, 126 MHz).

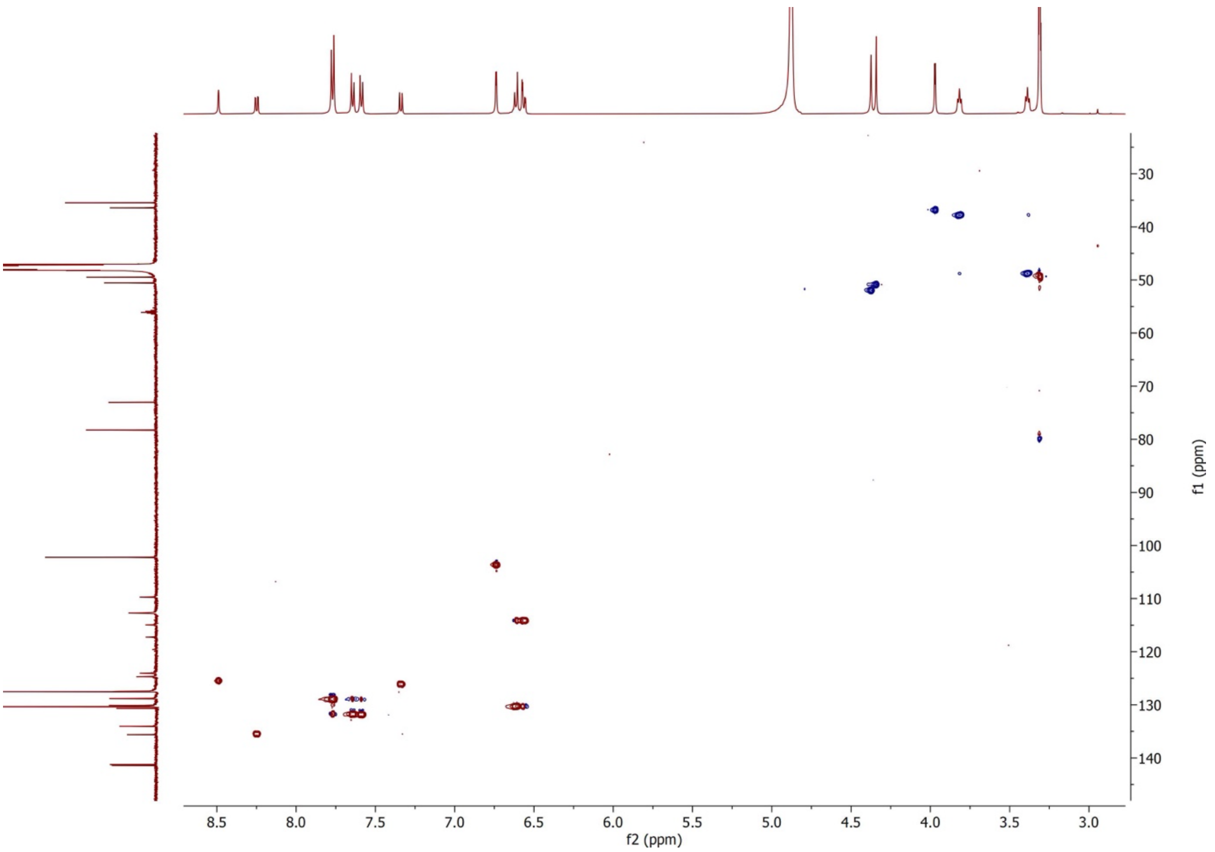

Figure S12. HSQC of compound **1** (MeOD).

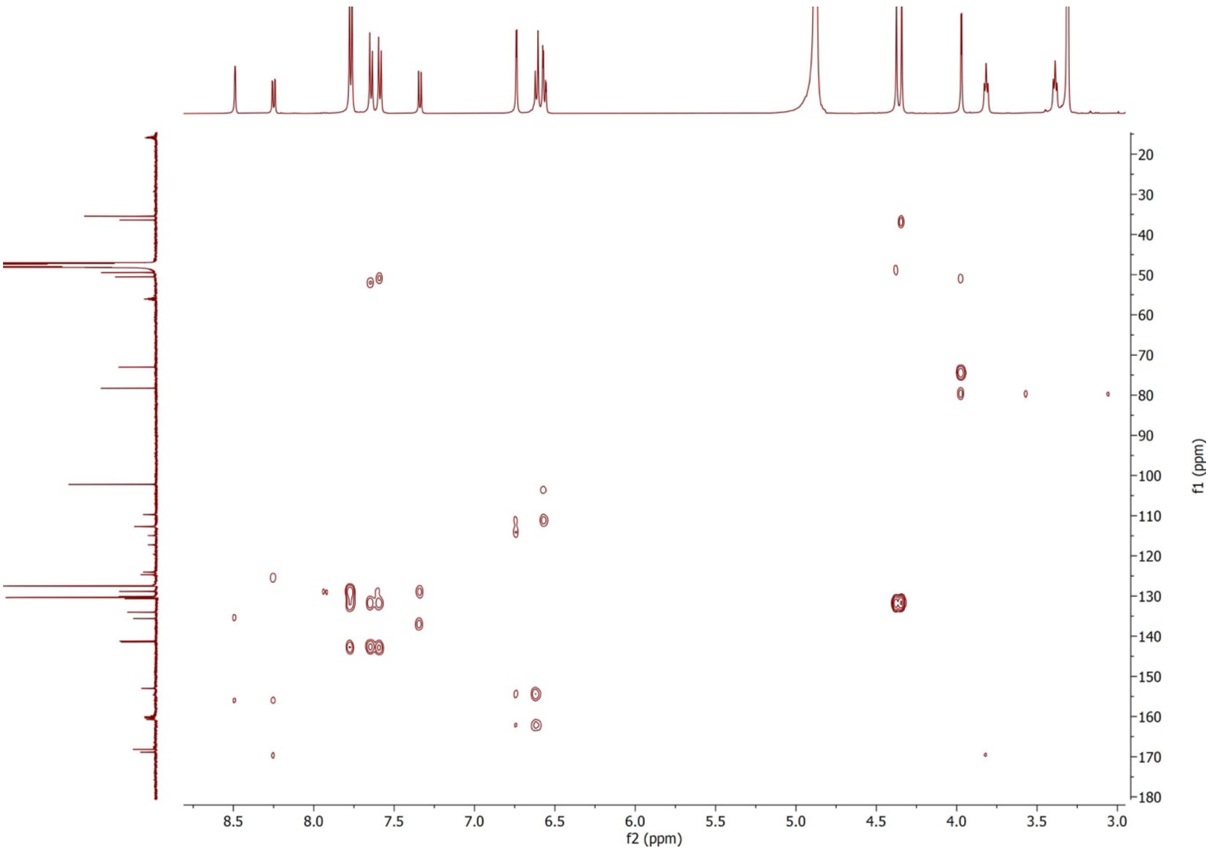

Figure S13. HMBC of compound **1** (MeOD).

^{1}H NMR titration for **1**⊃β-CD

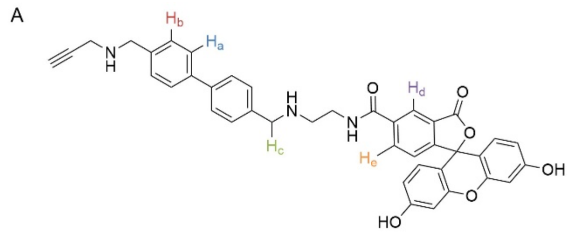

Sample preparation

2.5 mM stock solutions of β-CD and **1** were prepared in D_2O, with TMS used as an internal reference for the calibration of the spectra. NMR samples were prepared with different proportions of the host and the guest stock solutions so that the total volume for each sample was 0.5 mL, the total concentration of ([β-CD] + [**1**]) per sample was 2.5 mM, and the mole fraction r_X = [X]/([**1**] + [β-CD]) was between 0 and 1, with X = β-CD or **1** and [β-CD] and [**1**] the concentrations of the host (β-CD) and **1**, respectively. For the evaluation of the binding constant K, β-CD was added by portion to an NMR tube containing a 0.25 mM solution of **1**. ^{1}H NMR spectra were recorded before each new addition. The ratios were calculated from the NMR spectra.

Data confirm the 1:1 stoichiometry of the **1**⊃β-CD inclusion complex.

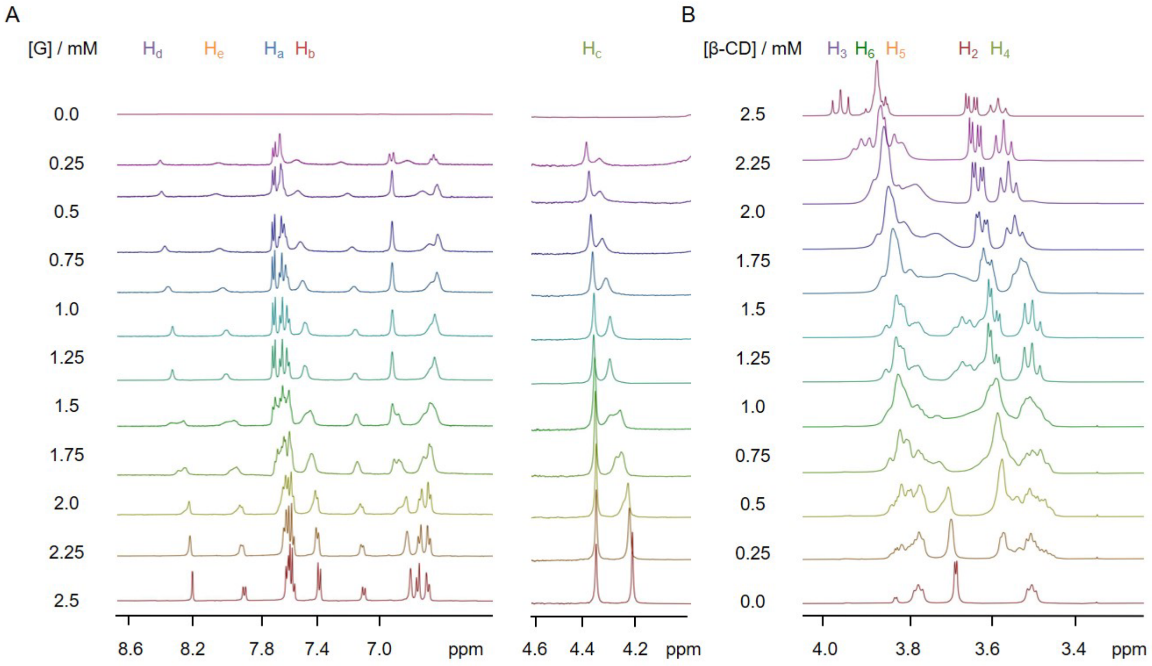

Figure S14. Selected regions of the ^{1}H NMR spectra of (A) **1** when [**1**] increases, and (B) β-CD (except H_1) when [β-CD] decreases but [**1**] + [β-CD] = 2.5 mM.

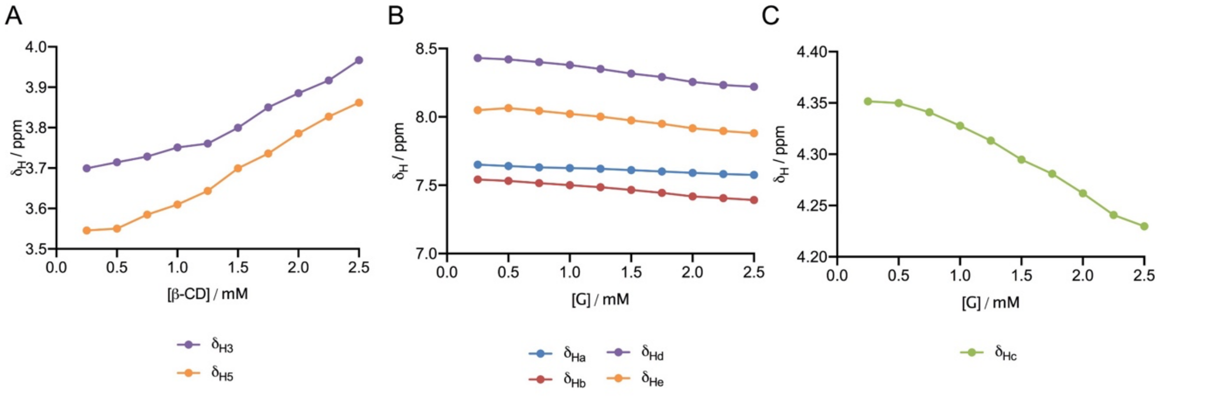

Figure S15. Chemical shifts variation of selected proton resonance peaks for (A) β-CD, and (B) and (C) **1**, as a function of substrate concentration.

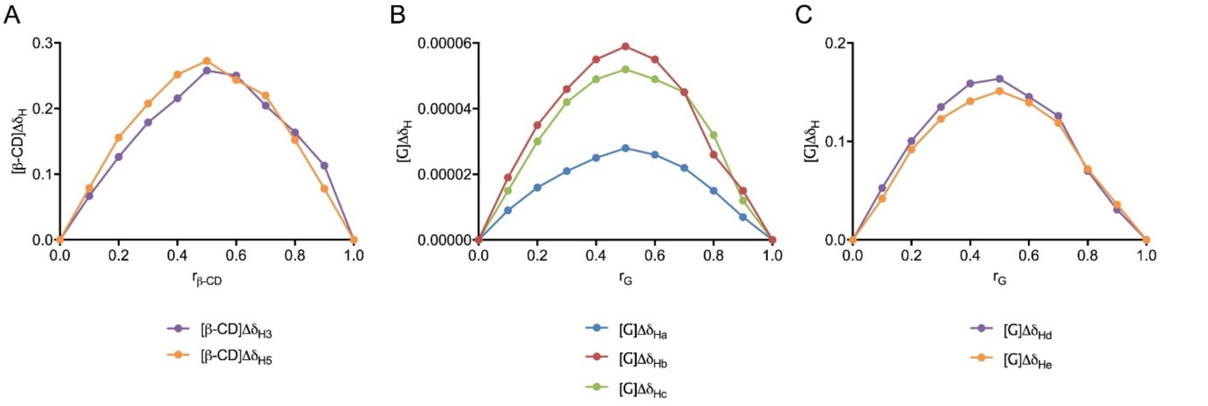

Figure S16. Job's plots corresponding to the induced chemical shift variation of selected resonance peaks associated with (A) β-CD, and (B) and (C) **1** protons during the formation of the **1**⊃β-CD inclusion complex.

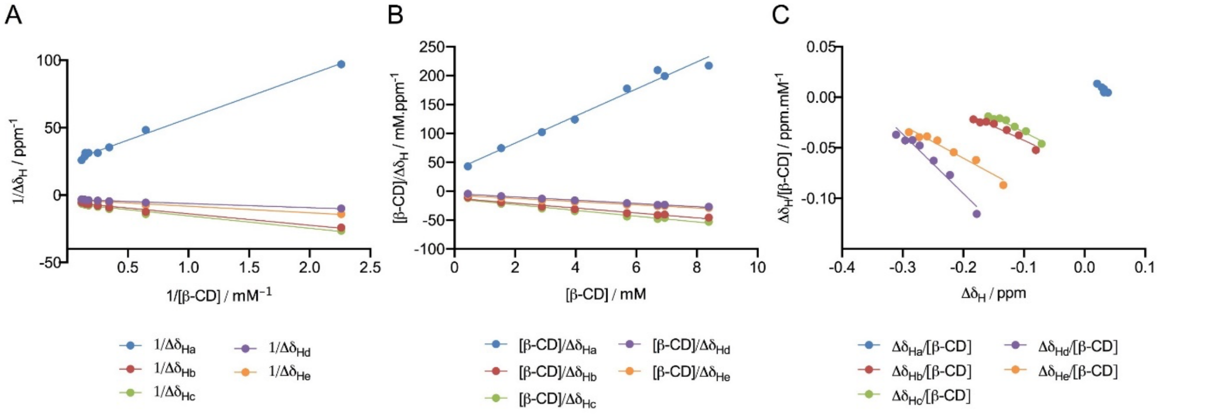

Figure S17. (A) Benesi-Hildebrand, (B) Scott, and (C) Scatchard plots for the **1**⊃β-CD inclusion complex.

*Synthesis of the [3]pseudorotaxane **4** and the metallo[3]rotaxane ^{nat}Ga-**4***

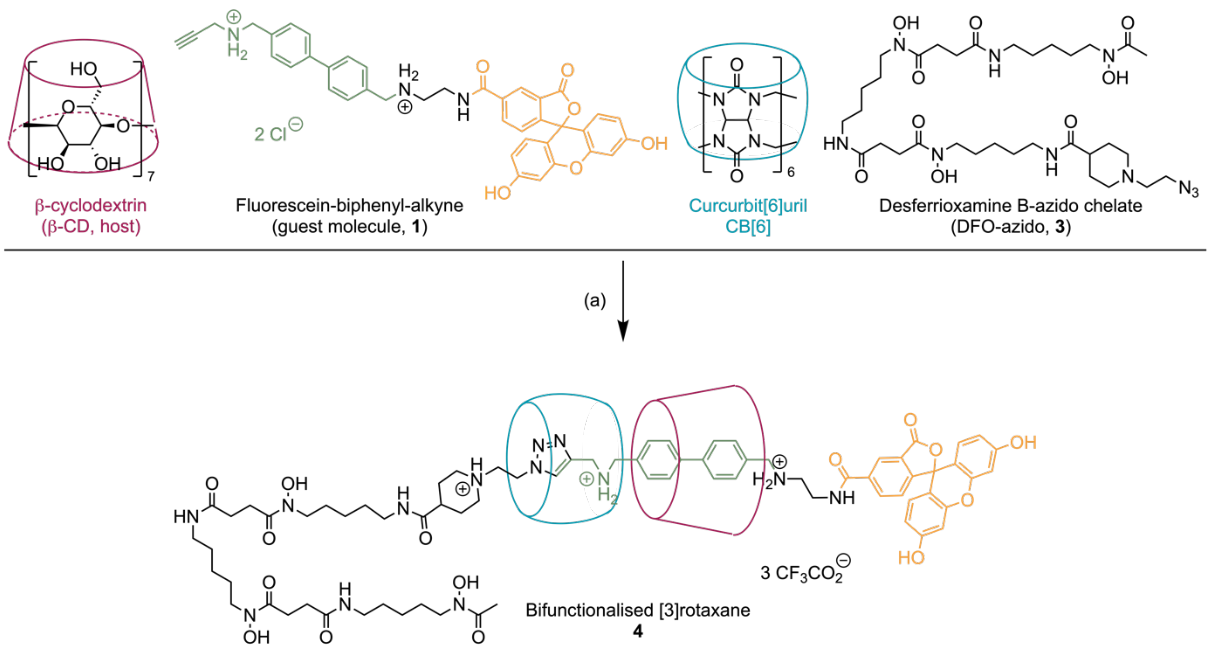

Scheme S2. Synthesis of [3]semirotaxane **4**: (a) H_2O, 70 °C, 1 min, 72%.

Compound 4

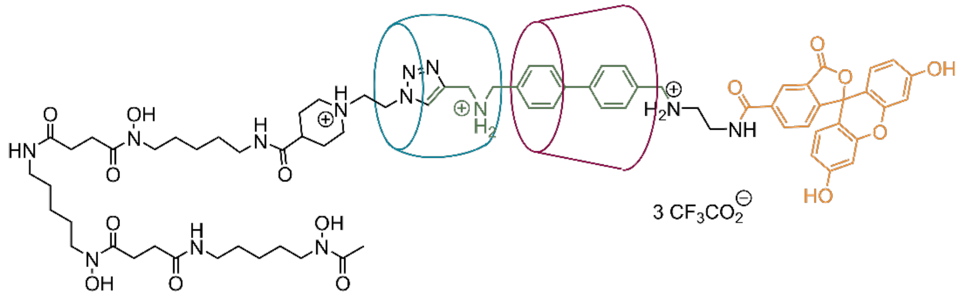

To a solution of **1** (1.0 mg, 1.5 μmol, 1 equiv.) and β-CD (1.8 mg, 1.5 μmol, 1 equiv.) in H_2O (0.5 mL) were added **3** (1.1 mg, 1.5 μmol, 1 equiv.) and CB[6] (1.5 mg, 1.5 μmol, 1 equiv.). The reaction mixture was heated to 70 °C for 1 min. The crude was purified by using preparative HPLC at a flow rate of 7 mL min^{-1} with a linear gradient of A (MeOH, Sigma-Aldrich, HPLC grade) and B (distilled water containing 0.1% TFA): *t* = 0 min A 5% + B 95%, *t* = 30 min A 100% + B 0%. After lyophilisation, **4** was obtained as a white powder (3.9 mg, 72% yield). The product was estimated by analytical HPLC to have a purity >95%; ^{1}H NMR (500 MHz, D_2O) δ = 8.74 (*d*, *J* = 1.3 Hz, 1H), 8.29 (*dd*, *J* = 1.3, 8.0 Hz, 1H), 8.07 (*d*, *J* = 8.1, Hz, 2H), 7.68 (*d*, *J* = 8.1 Hz, 2H), 7.66 (*d*, *J* = 8.0 Hz, 1H), 7.58 (*d*, *J* = 8.0, Hz, 1H), 7.55 (*d*, *J* = 8.0 Hz, 2H), 7.48 (*dd*, *J* = 1.5, 9.0, Hz, 2H), 7.35 (*dd*, *J* = 1.5, 1.5 Hz, 2H), 7.15 (*ddd*, *J* = 1.5, 1.5, 9.1, Hz, 2H), 6.56 (*s*, 1H), 5.69−5.79 (*m*, 12H), 5.49 (*s*, 12H), 5.01−5.06 (*m*, 7H), 4.60 (*s*, 2H), 4.16−4.46 (*m*, 20H), 3.91−3.96 (*m*, 2H), 3.47−3.77 (*m*, 48H), 3.45−3.47 (*m*, 2H), 3.07−3.29 (*m*, 10H), 2.60−2.71 (*m*, 5H), 2.42−2.55 (*m*, 4H), 2.16−2.30 (*m*, 2H), 2.16 (*s*, 3H), 2.04−2.30 (*m*, 2H), 1.57−1.71 (*m*, 6H), 1.47−1.57 (*m*, 6H), 1.23−1.38 ppm (*m*, 6H); $^{13}C\{^{1}H\}$ NMR (126 MHz, D_2O) δ = 177.0, 177.0, 176.1, 174.8, 174.7, 174.6, 173.8, 173.5, 170.5, 169.7, 169.7, 165.0, 163.0 (*q*, ^{2}J = 35.6 Hz), 156.5, 156.3, 156.3, 156.1, 155.6, 141.4, 139.9, 139.1, 135.2, 132.6, 131.0, 130.9, 130.8, 130.7, 127.8, 126.8, 126.6,

126.0, 119.6, 116.7, 116.6, 116.3 (*q*, ^{1}J = 291.9 Hz), 113.1, 113.0, 102.8, 102.4, 81.7, 73.3, 72.0, 71.8, 70.4, 70.3, 70.2, 70.1, 60.0, 55.4, 52.8, 52.4, 52.0, 51.5, 51.5, 51.4, 51.4, 50.8, 50.5, 47.8, 47.7, 47.2, 44.8, 44.5, 42.8, 40.0, 39.8, 39.2, 39.1, 38.9, 38.8, 36.5, 30.4, 30.4, 30.3, 29.4, 28.7, 27.9, 27.8, 27.6, 26.4, 25.7, 25.4, 23.0, 23.0, 22.9, 22.5, 19.2 ppm; HRMS (ESI) *m/z* calcd for $C_{151}H_{203}N_{37}O_{62}$ $[M+4H]^{4+}$ 881.5962 found 881.5963 (100); calcd for $C_{151}H_{202}N_{37}O_{62}$ $[M+3H]^{3+}$ 1175.1258, found 1175.1264 (100).

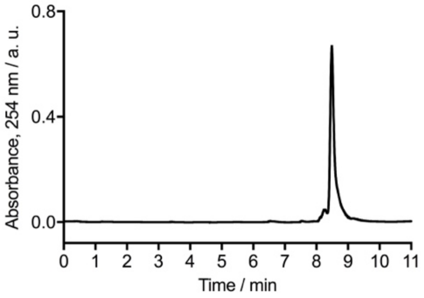

Figure S18. Reverse-phase analytical HPLC chromatogram of complex **4**, λ= 254 nm.

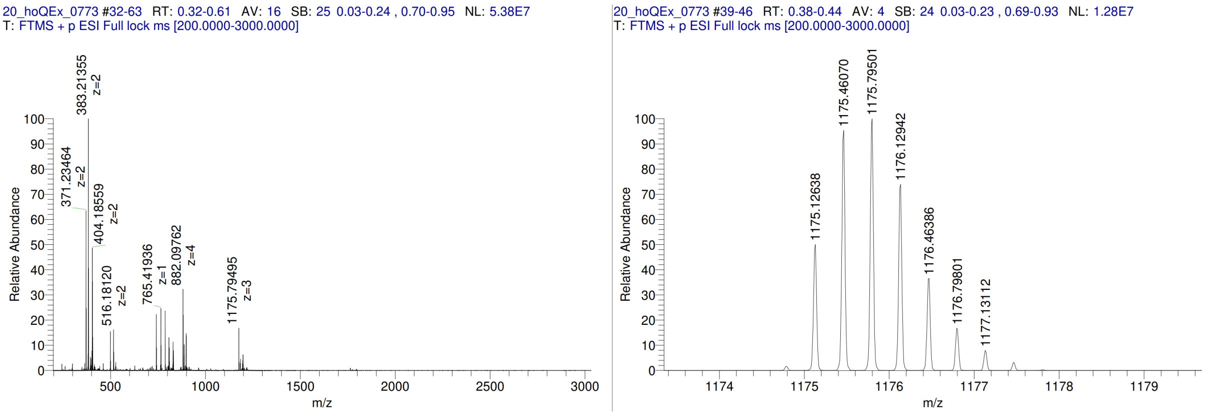

Figure S19. HRMS (ESI+) spectrum of compound **4**.

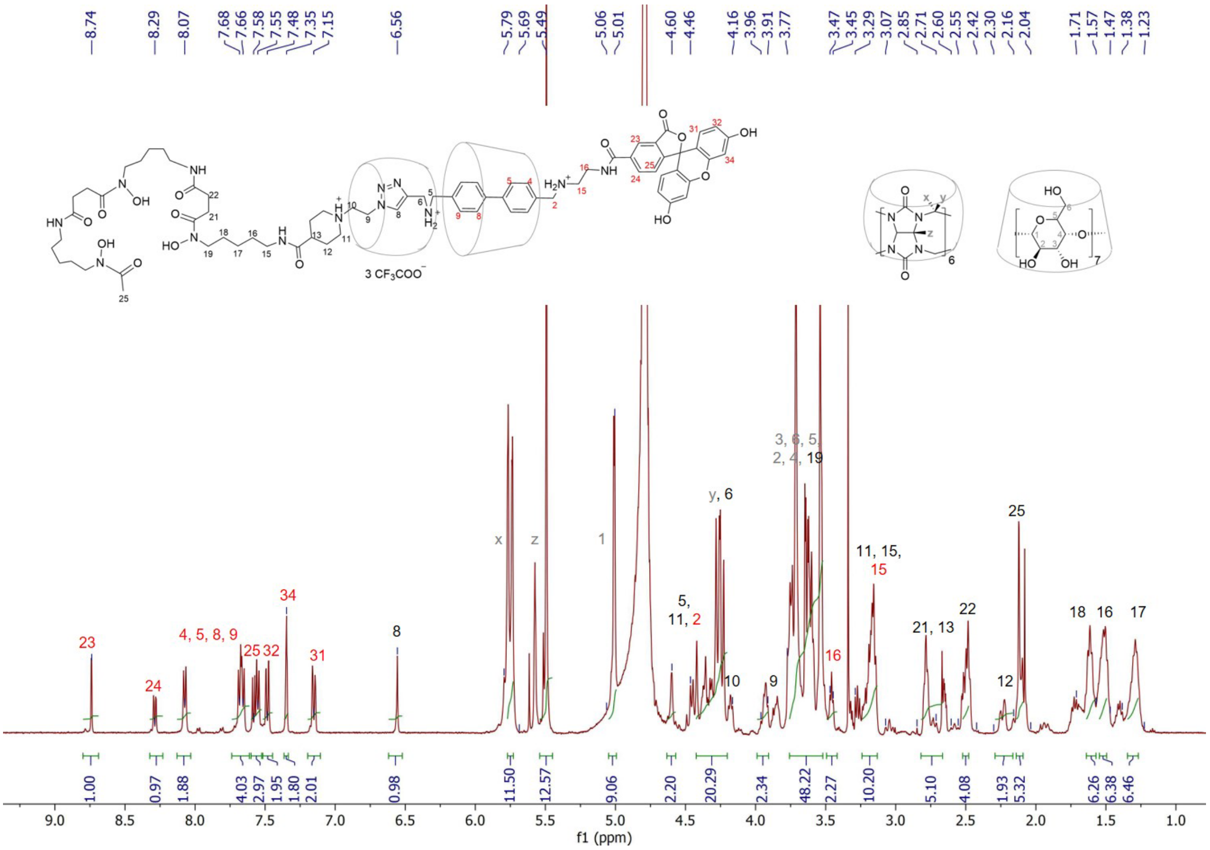

Figure S20. ^{1}H NMR of compound **4** (D_2O, 500 MHz).

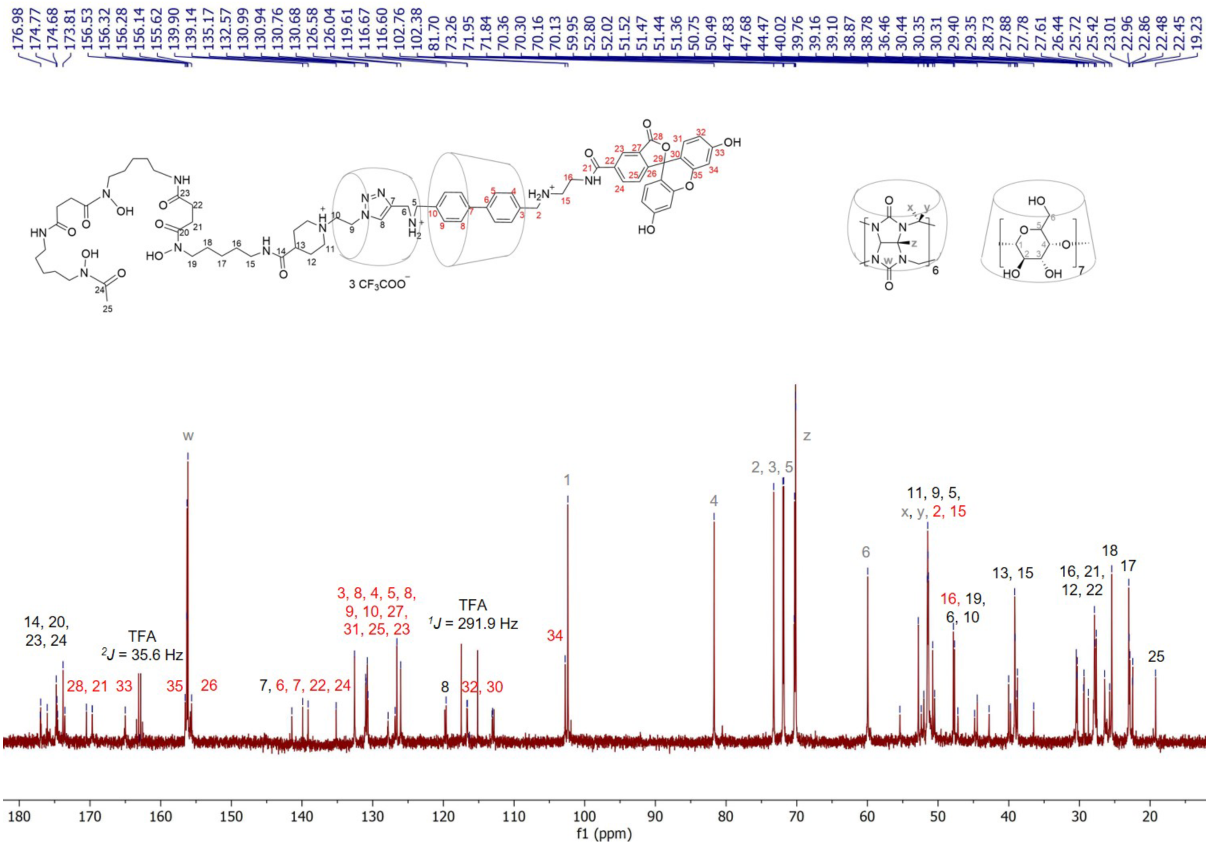

Figure S21. $^{13}C\{^{1}H\}$ NMR of compound **4** (D_2O, 126 MHz).

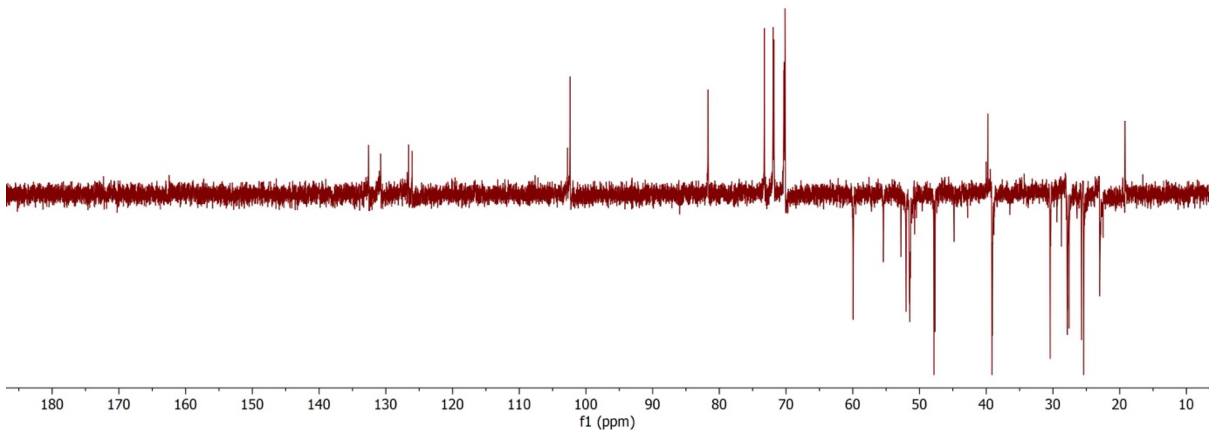

Figure S22. DEPT-135 of compound **4** (D_2O).

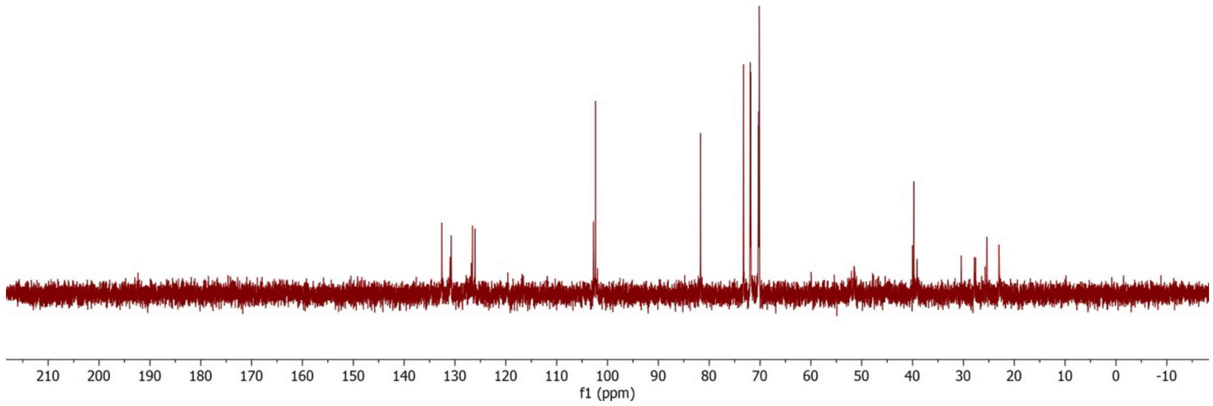

Figure S23. DEPT-90 of compound **4** (D_2O).

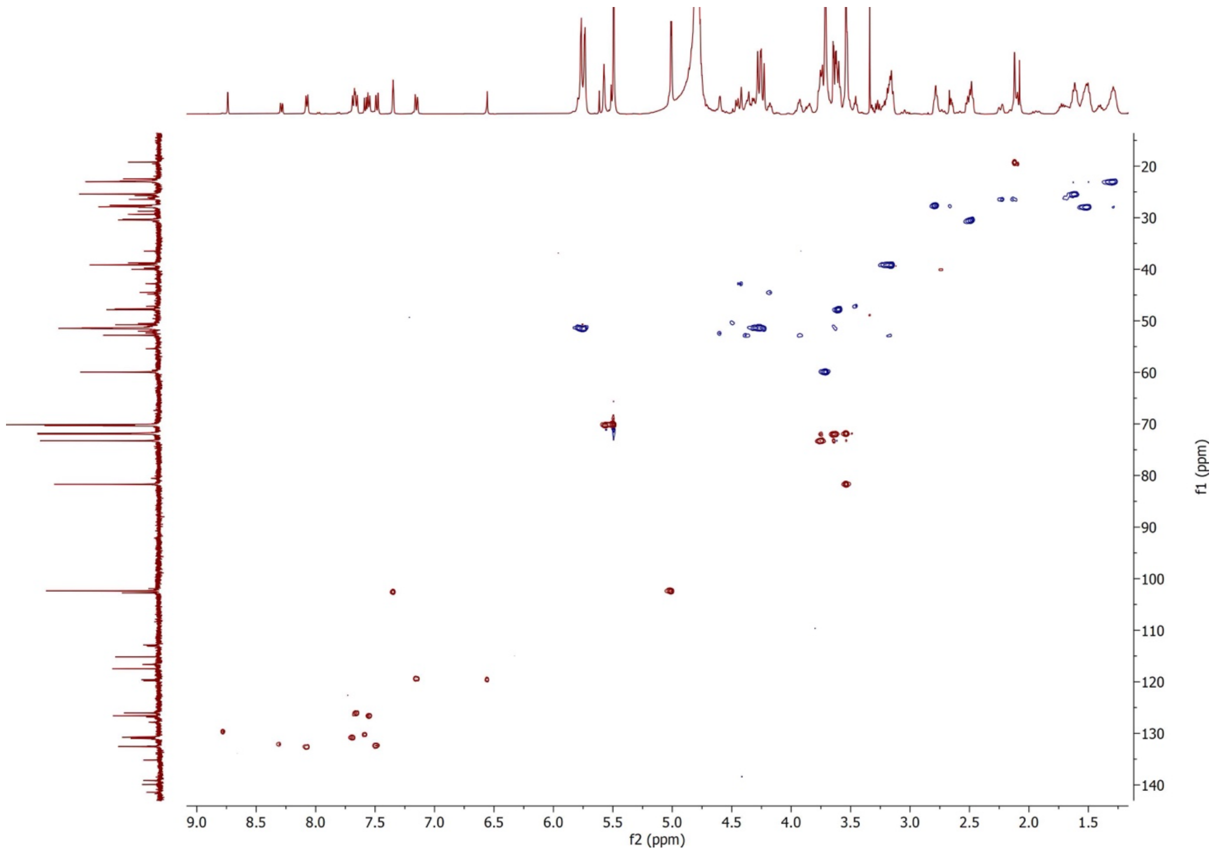

Figure S24. HSQC of compound **4** (D_2O).

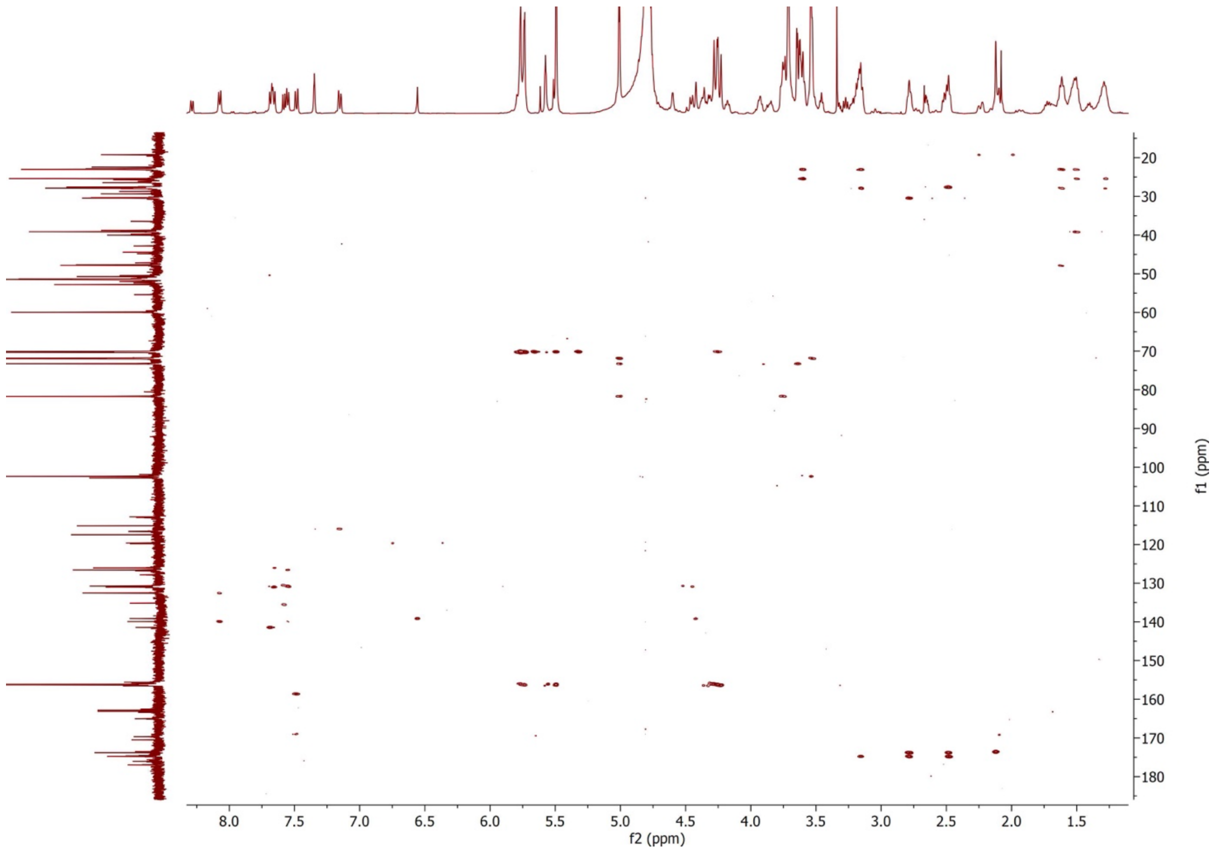

Figure S25. HMBC of compound **4** (D_2O).

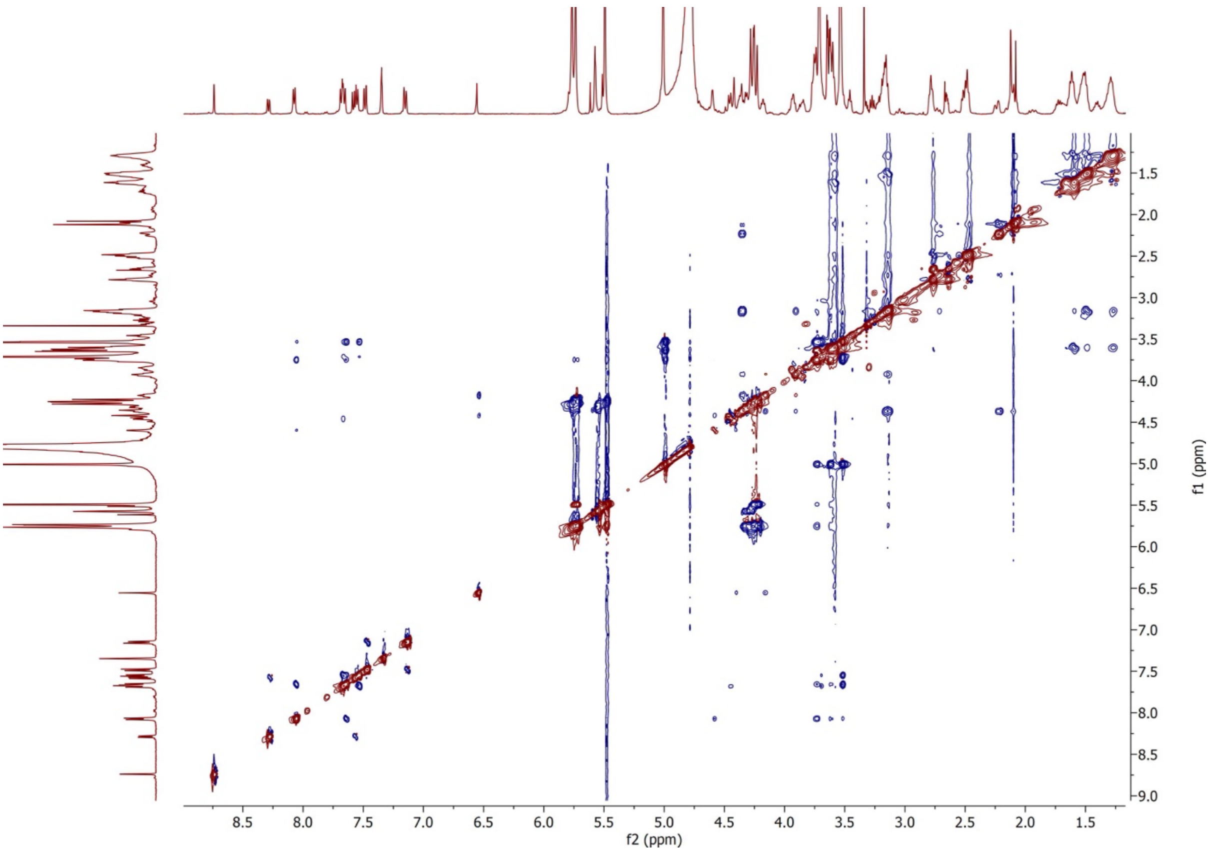

Figure S26. ROESY of compound **4** (D_2O).

Metallo[3]rotaxane ^{nat}Ga-4

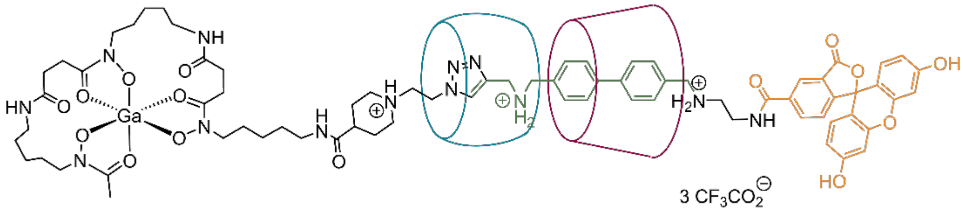

Following General procedure A, ^{nat}Ga-**4** was obtained as a slightly yellow residue. The product was estimated by analytical HPLC to have a purity >95%; HRMS (ESI) *m/z* calcd for $C_{151}H_{201}GaN_{37}O_{62}$ $[M+3H]^{3+}$ 1197.7651 found 1197.7630 (100).

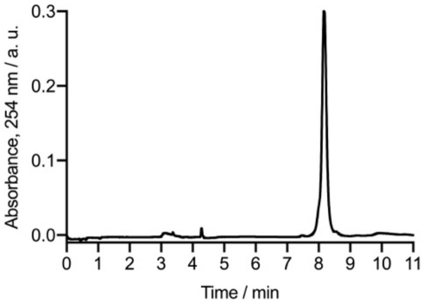

Figure S27. Reverse-phase analytical HPLC chromatogram of complex ^{nat}Ga-**4**, λ= 254 nm.

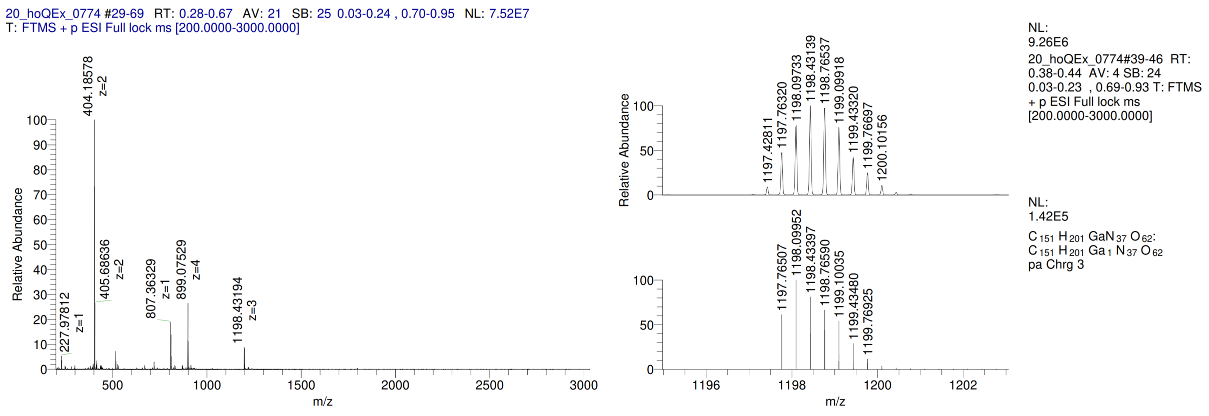

Figure S28. HRMS (ESI+) spectrum of compound ^{nat}Ga-**4**.

*Radiosynthesis of [^{68}Ga]Ga-**4***

Radiolabelling reactions to prepare [^{68}Ga]Ga-**4** were accomplished by the addition of an aliquot of [^{68}Ga][Ga(H_2O)$_6$]Cl_3(aq.) stock solution (~12 MBq, diluted in H_2O to ~190 μL) to an aqueous solution of **4** (10 μL of 1 mM stock in H_2O) buffered with NaOAc (0.2 M, pH4.4, 50 μL) with a total reaction volume of 250 μL. The reactions were monitored by radio-iTLC (citrate buffer, 1.0 M, pH4.5) and complexation was found to be complete in less than 10 min at 23 ºC giving a radiochemical conversion (RCC) >99% (R_f = 0.0 – 0.1). The product was characterised by analytical HPLC following the method described in the general section. Note: the UV-Vis detector and radioactivity detector were arranged serially with an offset time of approximately 0.10-0.30 min (depending on temperature). After optimisation [^{68}Ga]Ga-**4** was isolated with a molar activity of 20 MBq nmol^{-1} (measured with titration experiments, R^2 = 0.8648). The identity of the radiolabelled compound ([^{68}Ga]Ga-**4**) was confirmed by co-injection with an authenticated sample of non-radiolabelled complex ^{nat}Ga-**4**.

*Synthesis of the fluorescein-azido compound **5***

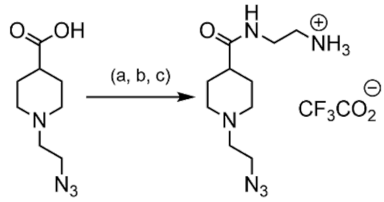

13 14

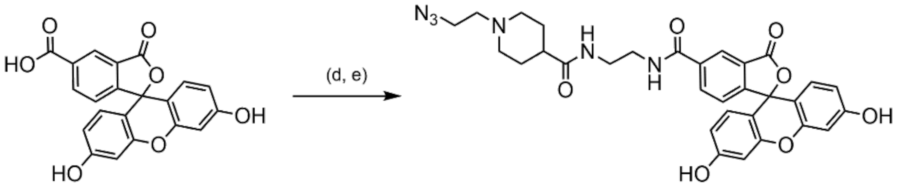

5-carboxyfluorescein 5

Scheme S3. Synthesis of **5**: (a) NHS, EDC·HCl, DMF, 23 °C, 2 h; (b) Boc-1,2-diaminoethane, Et_3N, DMF, 23 °C, 12 h; (c) TFA, CH_2Cl_2, 0 to 23 °C, 2 h, 29%; (d) NHS, EDC·HCl, DMF, 23 °C, 2 h; (e) **14**, Et_3N, DMF, 23 °C, 12 h, 94%.

Compound 14

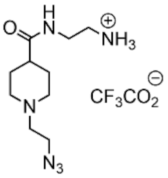

To a solution of **13** (35 mg, 0.18 mmol, 1 equiv.) in DMF (1 mL) were added EDC·HCl (37 mg, 0.19 mmol, 1.1 equiv.) and *N*-hydroxysuccinimide (22 mg, 0.19 mmol, 1.1 equiv.). The resulting solution was stirred at 23 °C for 2 h. Then Boc-1,2-diaminoethane (30 mg, 0.19 mmol, 1.1 equiv.) and Et_3N (0.029 mL, 0.20 mmol, 1.2 equiv.) were added and the reaction mixture was stirred for 12 h at 23 °C. Then DMF was evaporated under reduced pressure. The bright yellow residue was dissolved in CH_2Cl_2 (1 mL), ice cooled and TFA (1 mL) was added. The resulting mixture was warmed up to 23 °C, protected from light and stirred at 23 °C for 2 h. The solvent was then removed under reduced pressure and the crude residue was washed with Et_2O to afford **14** (20 mg, 29% yield) as a slightly yellow oil. ^{1}H NMR (500 MHz, D_2O) δ = 3.86 (*m*, 2H), 3.70−3.72 (*m*, 2H), 3.52 (*m*, 2H), 3.34 (*m*, 2H), 3.15 (*m*, 2H), 3.03−3.10 (*m*, 2H), 2.62−2.67 (*m*, 1H), 2.09−2.16 (*m*, 2H), 1.91−2.02 ppm (*m*, 2H); ^{13}C{^{1}H} NMR (126 MHz, D_2O) δ = 176.8, 162.9 (*q*, ^2J = 35.7 Hz), 116.3 (*q*, ^1J = 291.9 Hz), 55.4, 52.0, 44.8, 39.5, 39.0, 36.9, 25.6 ppm; HRMS (ESI) *m/z* calcd for $C_{10}H_{20}N_6O$ $[M+Na]^+$ 263.1591, found 263.1584 (100).

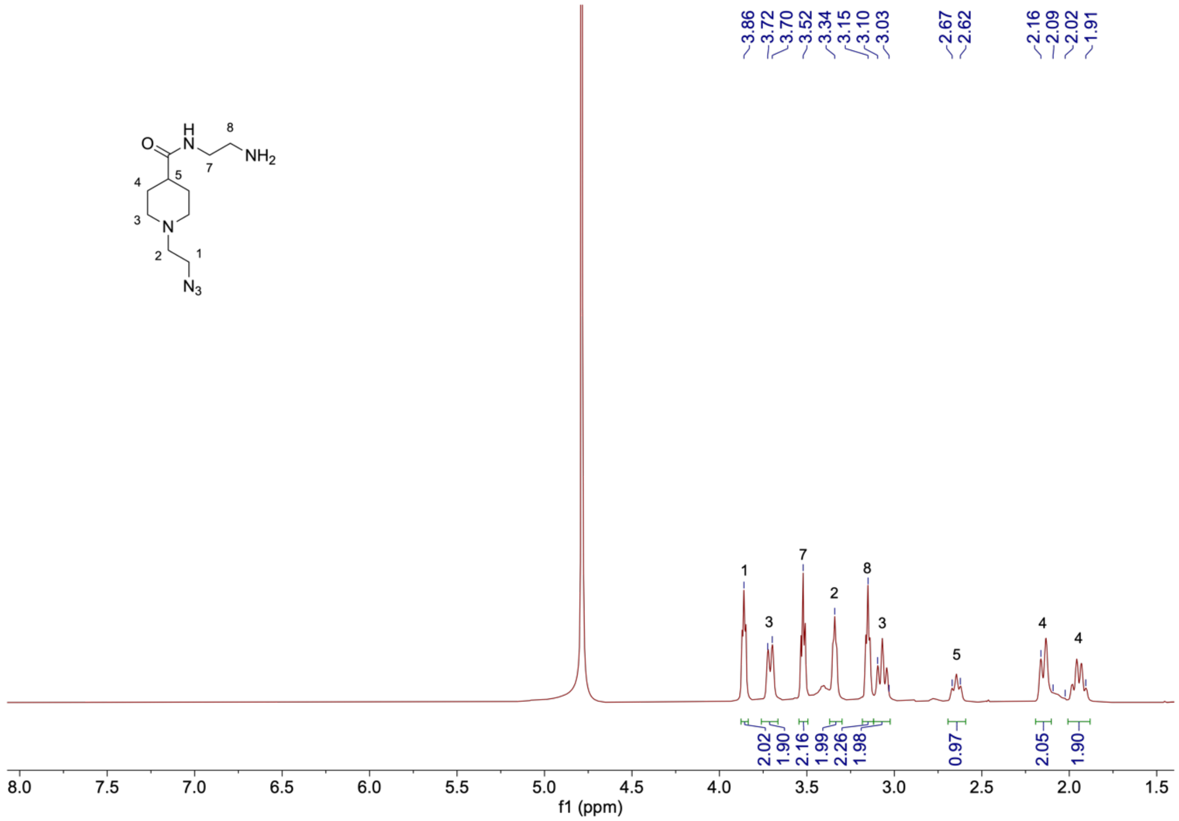

Figure S29. ^{1}H NMR of compound **14** (D_2O, 500 MHz).

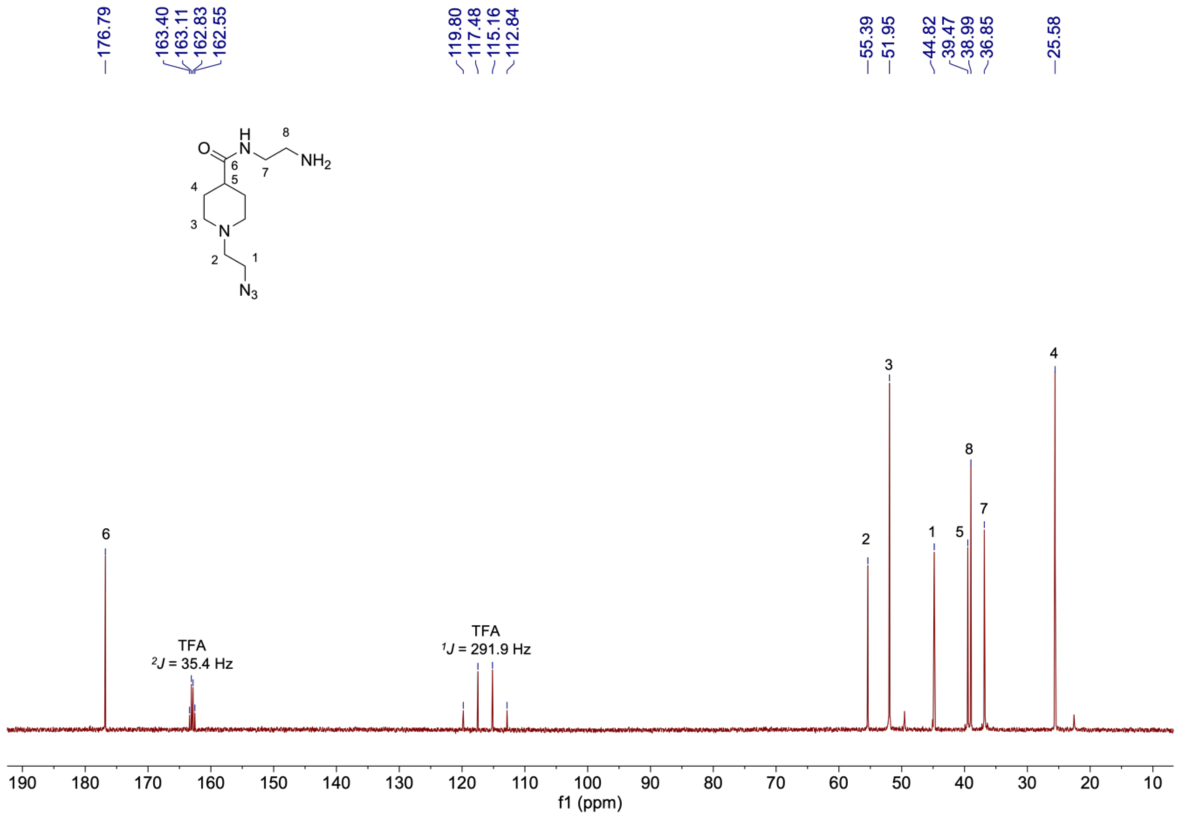

Figure S30. $^{13}C\{^{1}H\}$ NMR of compound **14** (D_2O, 126 MHz).

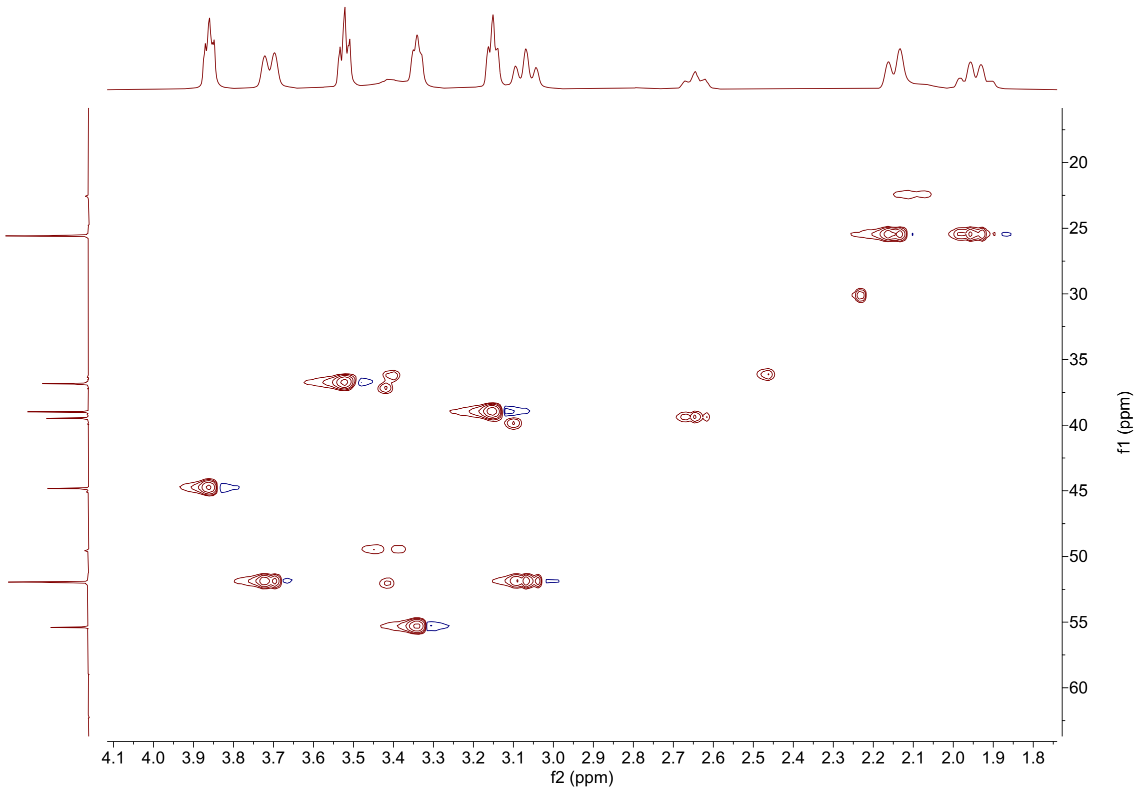

Figure S31. HSQC of compound **14** (D_2O).

Compound 5 (Fluorescein-azido)

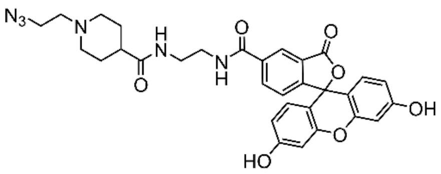

To a solution of 5-carboxyfluorescein (17 mg, 0.05 mmol, 1.1 equiv.) in DMF (1 mL) were added EDC·HCl (9 mg, 0.05 mmol, 1.1 equiv.) and *N*-hydroxysuccinimide (5 mg, 0.05 mmol, 1.1 equiv.). The resulting solution was stirred at 23 °C for 2 h. Then **14** (10 mg, 0.04 mmol, 1 equiv.) and Et_3N (0.007 mL, 0.05 mmol, 1.2 equiv.) were added and the reaction mixture was stirred for 12 h at 23 °C. Then DMF was evaporated under reduced pressure. The crude was purified by using semi-preparative HPLC at a flow rate of 7 mL min^{-1} with a linear gradient of A (MeOH, Sigma-Aldrich, HPLC grade) and B (distilled water containing 0.1% TFA): t = 0 min A 5% + B 95%, t = 30 min A 100% + B 0%. The sample was lyophilised to afford **5** (63 mg, 94% yield) as a bright yellow residue; The product was estimated by analytical HPLC to have a purity >95%; ^{1}H NMR (500 MHz, MeOD) δ = 8.43 (*d*, *J* = 1.5 Hz, 1H), 8.19 (*dd*, *J* = 1.6, 8.0 Hz, 1H), 7.32 (*d*, *J* = 8.1 Hz, 1H), 6.74 (*d*, *J* = 2.3 Hz, 2H), 6.63 (*d*, *J* = 8.8 Hz, 2H), 6.58 (*dd*, *J* = 2.3, 8.8 Hz, 2H), 3.82 (*dd*, *J* = 5.4, 5.4 Hz, 2H), 3.64−3.66 (*m*, 2H), 3.57 (*dd*, *J* = 5.3, 5.3 Hz, 2H), 3.46 (*dd*, *J* = 6.2, 6.2 Hz, 2H), 3.31 (*t*, 2H, overlapping with MeOD), 2.97−3.03 (*m*, 2H), 2.48−2.59 (*m*, 2H), 1.90−2.13 ppm (*m*, 2H); ^{13}C{^{1}H} NMR (126 MHz, MeOD) δ = 174.1, 168.5, 166.9, 160.3 (*q*, ^{2}J = 35.7 Hz), 152.6, 136.1, 133.5, 128.5, 127.0, 124.2, 123.5, 116.3 (*q*, ^{1}J = 291.3 Hz), 112.3, 109.4, 101.8, 54.9, 51.6, 44.6, 39.2, 39.1, 38.4, 25.4 ppm; HRMS (ESI) *m/z* calcd for $C_{31}H_{31}N_6O_7$ $[M+H]^+$ 599.2249, found 599.2246 (100).

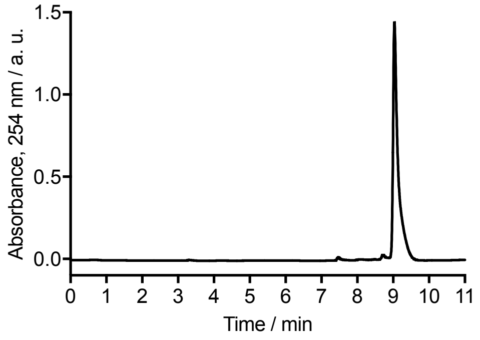

Figure S32. Reverse-phase analytical HPLC chromatogram of compound **5**, λ= 254 nm.

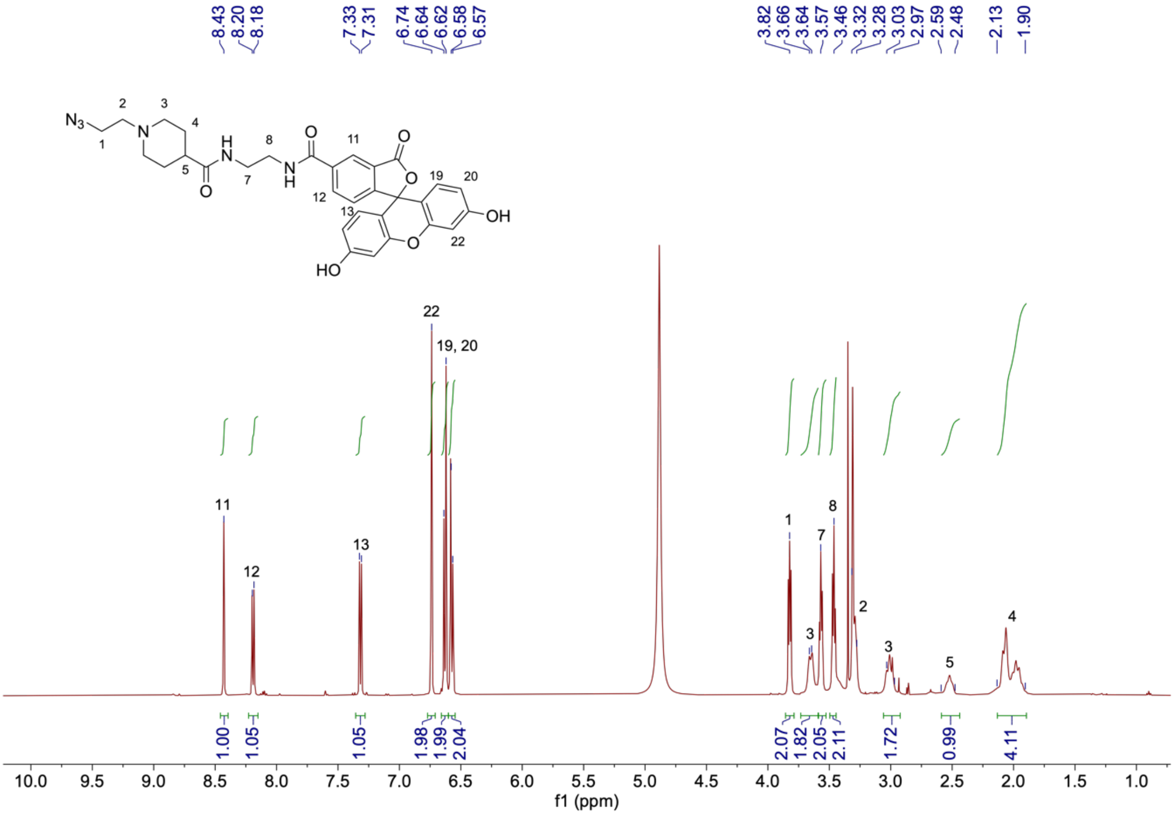

Figure S33. ^{1}H NMR of compound **5** (MeOD, 500 MHz).

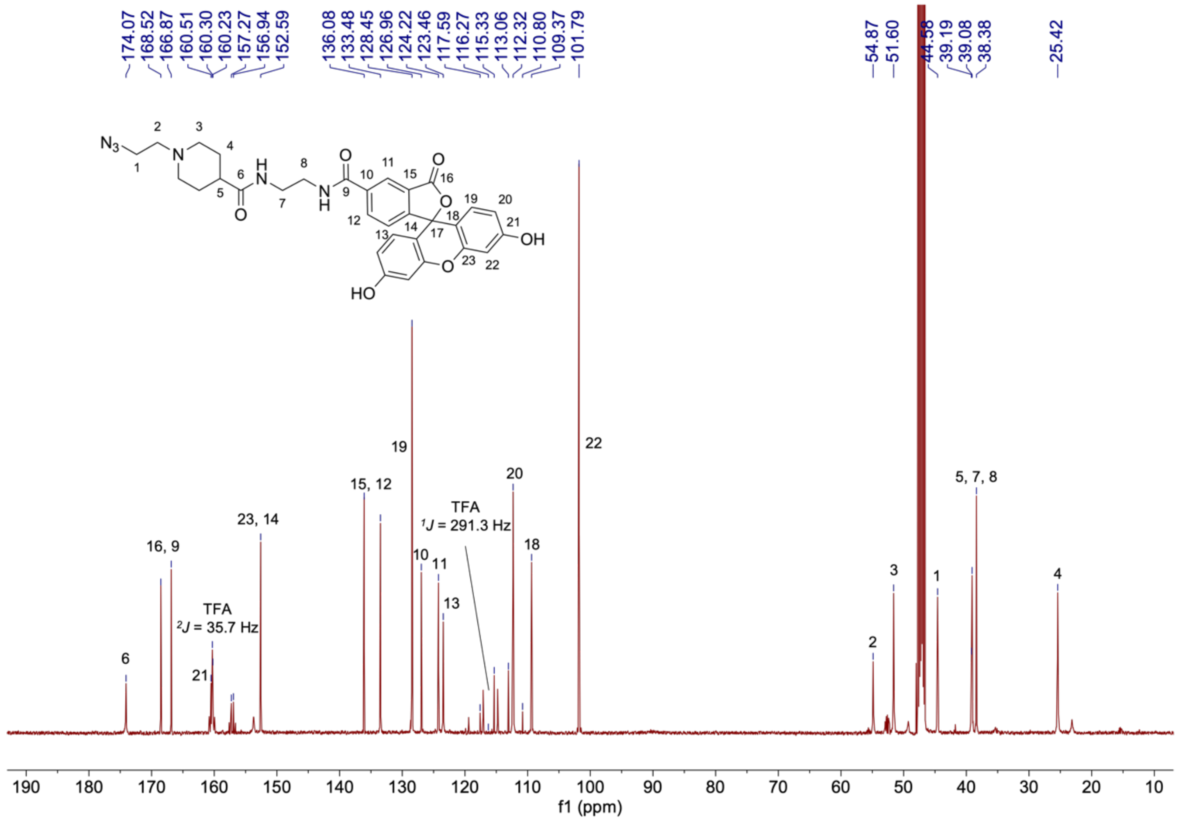

Figure S34. $^{13}C\{^1H\}$ NMR of compound **5** (MeOD, 126 MHz).

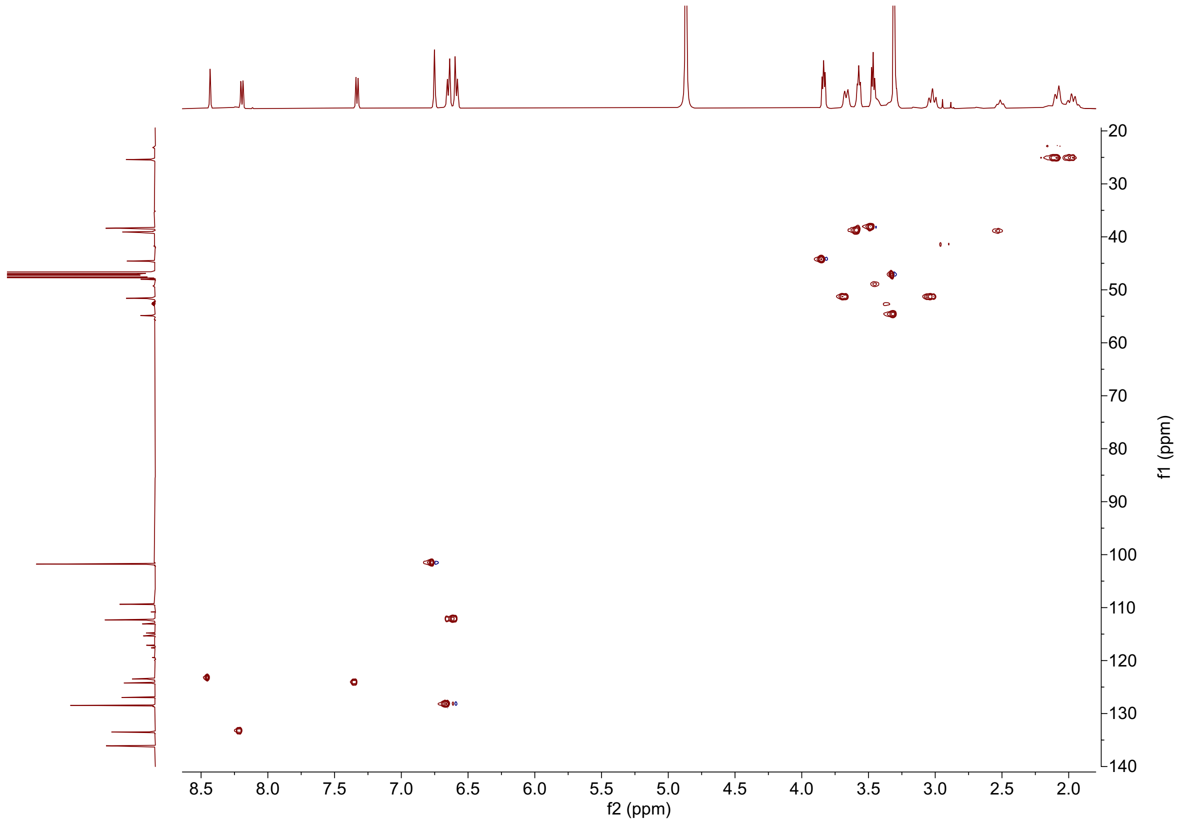

Figure S35. HSQC of compound **5** (MeOD).

*Synthesis of [4]semirotaxane **6***

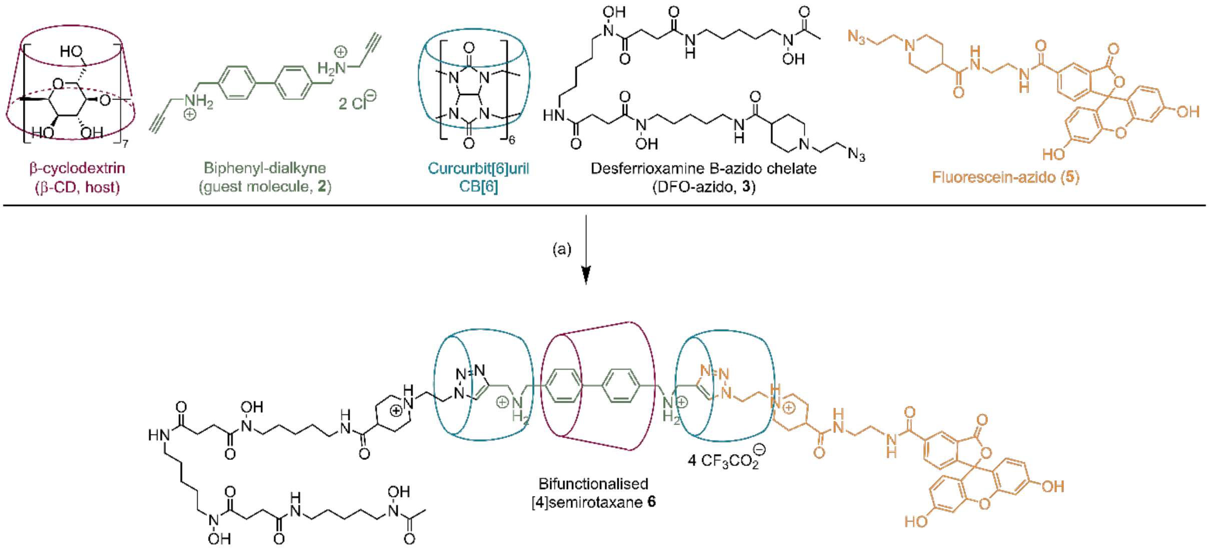

Scheme S4. Synthesis of [4]semirotaxane **6**: (a) H_2O, 70 °C, 1 min, 16%.

Compound 6

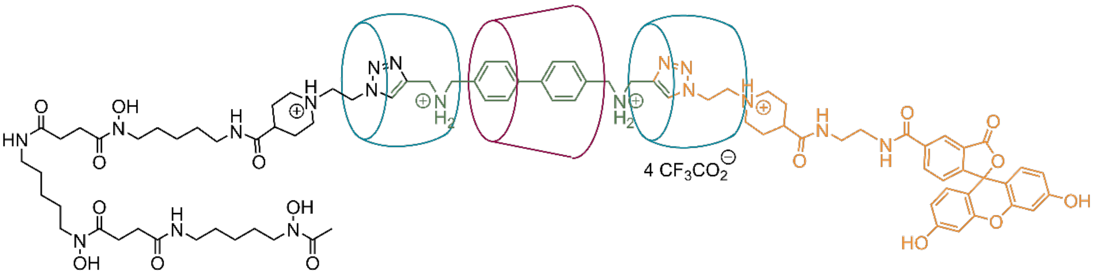

To a solution of **2** (0.70 mg, 2.4 μmol, 1 equiv.) and β-CD (2.75 mg, 2.4 μmol, 1 equiv.) in H_2O (0.5 mL) were added **5** (1.45 mg, 2.4 μmol, 2 equiv.), **3** (1.80 mg, 2.4 μmol, 1 equiv.), and CB[6] (4.82 mg, 4.8 μmol, 2 equiv.). The reaction mixture was heated to 70 °C for 5 min. The crude mixture was purified by using preparative HPLC at a flow rate of 7 mL min^{-1} with a linear gradient of A (distilled water containing 0.1% TFA) and B (MeOH, Sigma-Aldrich, HPLC grade): *t* = 0 min A 95% + B 5%, *t* = 30 min A 0% + B 100%. After lyophilisation, [4]semirotaxane **6** was obtained as a bright yellow powder (1.83 mg, 16% yield). The product was estimated by analytical HPLC to have a purity >95%; ^{1}H NMR (500 MHz, D_2O) δ = 8.39 (*s*, 1H), 8.06 (*dd*, *J* = 8.1, 8.1 Hz, 1H), 7.99 (*dd*, *J* = 8.0, 8.0 Hz, 2H), 7.91 (*dd*, *J* = 8.8, 8.8 Hz, 2H), 7.57 (*d*, *J* = 7.4 Hz, 2H), 7.42 (*d*, *J* = 7.8 Hz, 2H), 7.38 (*dd*, *J* = 8.1, 8.1 Hz, 2H), 6.92−7.02 (*m*, 4H), 6.65−6.72 (*m*, 2H), 6.72 (*s*, 1H), 6.65 (*s*, 1H), 5.52−5.76 (*m*, 24H), 5.32−5.48 (*m*, 24H), 4.92−4.99 (*m*, 7H), 4.47−4.60 (*m*, 4H), 4.27−4.42 (*m*, 6H), 3.96−4.27 (*m*, 28H), 3.71−3.92 (*m*, 6H), 3.41−3.92 (*m*, 48H), 3.01−3.22 (*m*, 10H), 2.64−2.77 (*m*, 4H), 2.37−2.48 (*m*, 4H), 1.91−2.19 (*m*, 8H), 2.05 (*s*, 3H), 1.49−1.67 (*m*, 6H), 1.39−1.49 (*m*, 6H), 1.17−1.34 ppm (*m*, 6H); ^{13}C{^{1}H} NMR (126 MHz, D_2O) δ = 176.0, 174.8, 173.8, 170.8, 169.7, 169.2, 163.0 (*q*, ^{2}J = 34.9 Hz), 156.3, 156.3, 156.3, 156.1, 156.1, 156.1, 155.4, 140.6, 139.9, 139.0, 139.0, 136.3, 135.9, 132.7, 131.6, 131.0, 130.9, 130.6, 126.0, 125.7, 125.7, 119.7, 116.3 (*q*, ^{1}J = 292.0 Hz), 112.6, 112.6, 103.0, 102.5, 81.3, 73.3, 71.9, 71.7, 70.2, 70.1, 70.1, 59.6, 52.8, 52.7, 52.0, 51.9, 51.4, 51.2, 47.8, 47.7, 44.5, 40.0, 40.0, 39.5, 39.2, 38.3, 30.4, 27.9, 27.9, 27.6, 26.4,

25.8, 25.4, 23.2, 23.0, 19.2 ppm; HRMS (ESI) *m/z* calcd for $C_{198}H_{256}N_{66}O_{75}$ [M+4H]$^{4+}$ 1189.4556 found 1189.4587 (100).

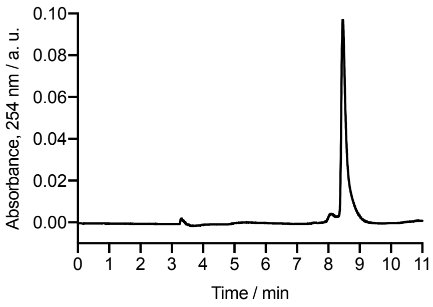

Figure S36. Reverse-phase analytical HPLC chromatogram of [4]semirotaxane **6**, λ = 254 nm.

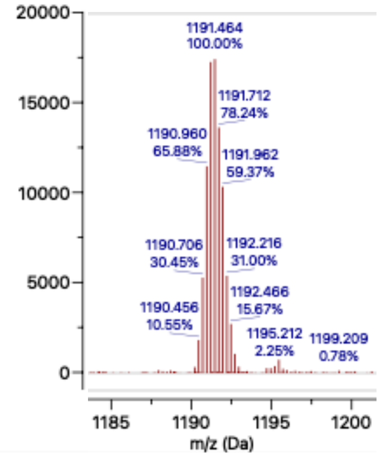

Figure S37. HRMS (ESI+) spectrum of [4]semirotaxane **6**.

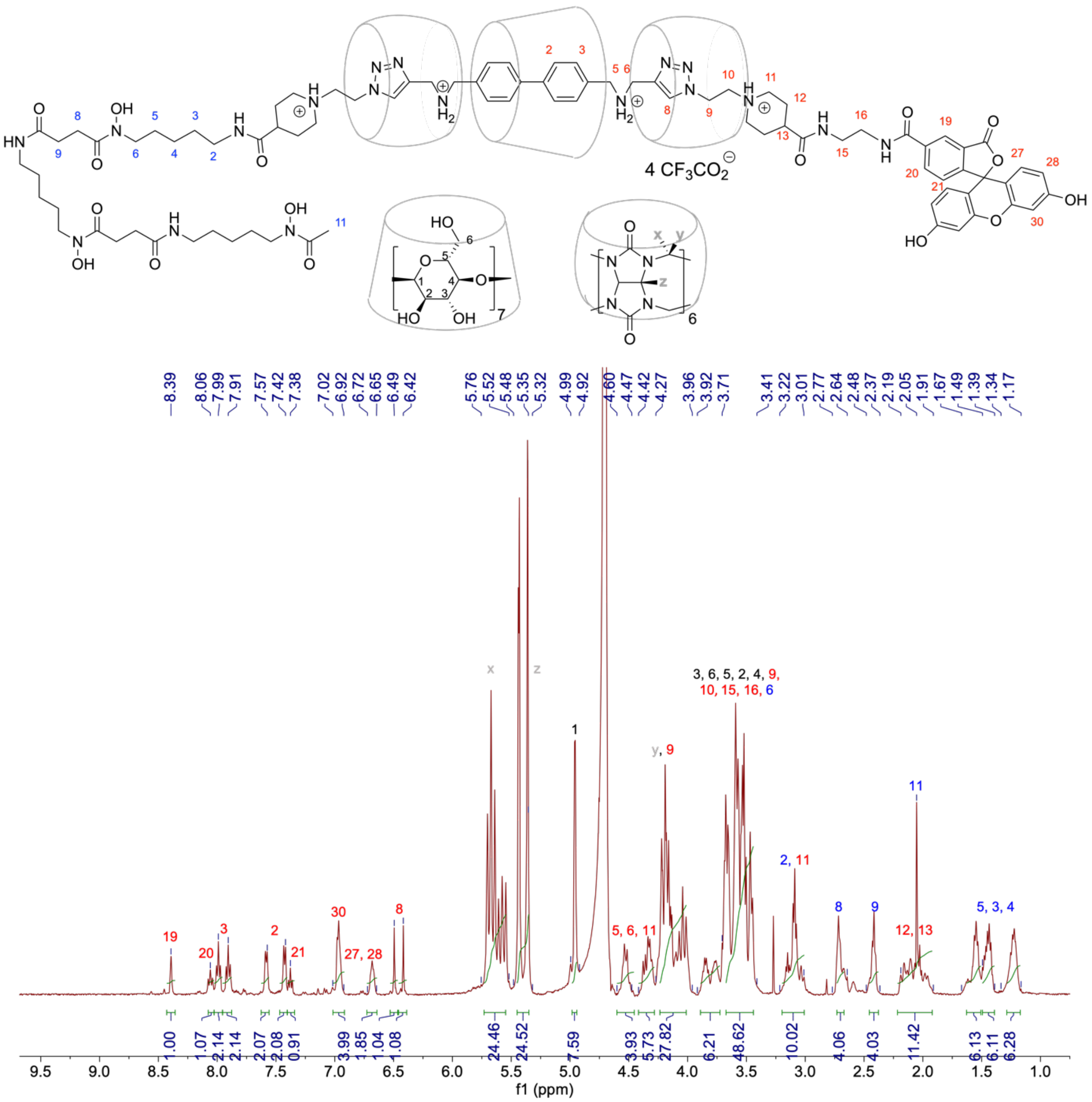

Figure S38. ^{1}H NMR of [4]semirotaxane **6** (D_2O, 500 MHz).

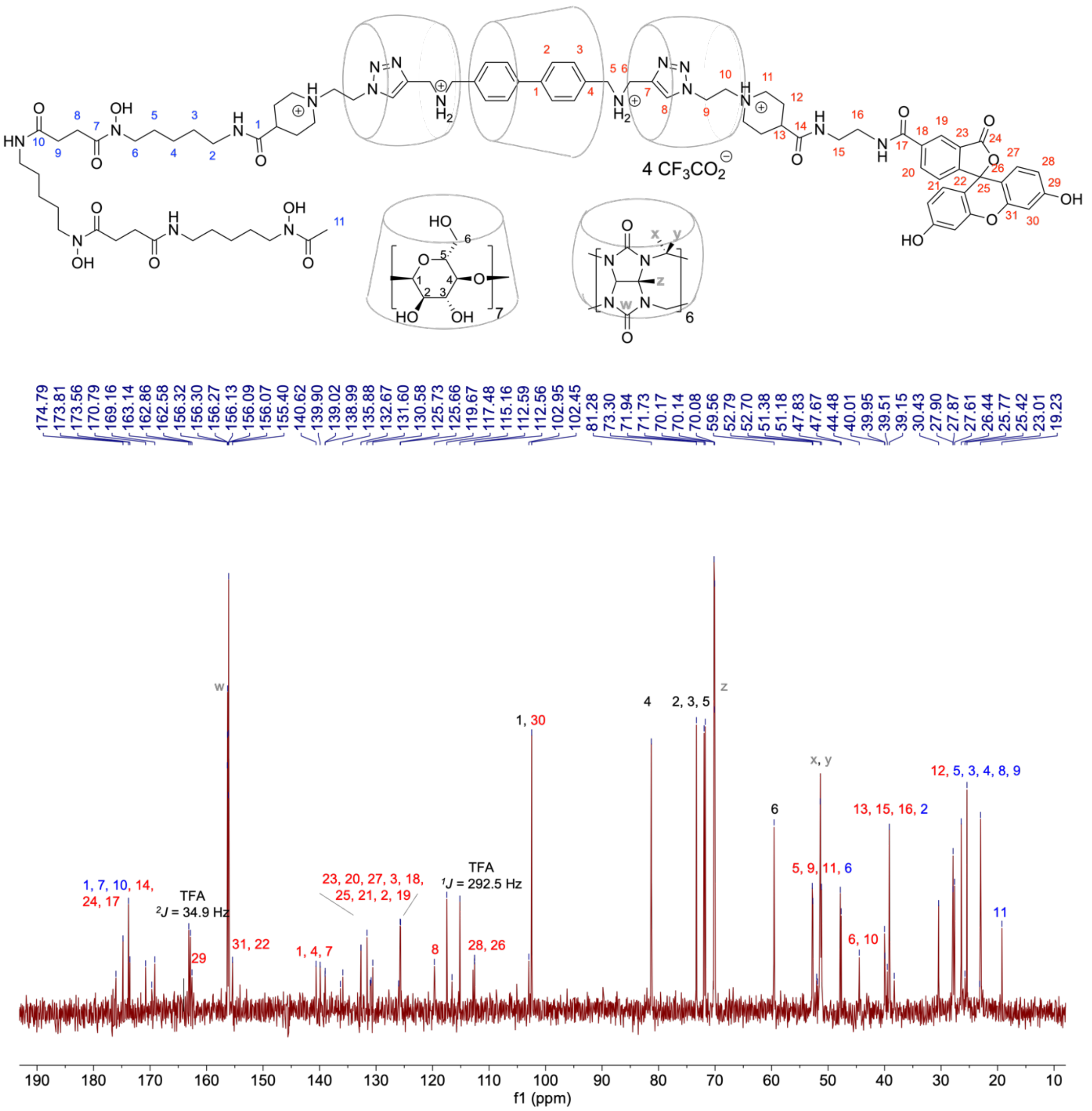

Figure S39. $^{13}C\{^{1}H\}$ NMR of [4]semirotaxane **6** (D_2O, 126 MHz).

Metallo[4]rotaxane natGa-6

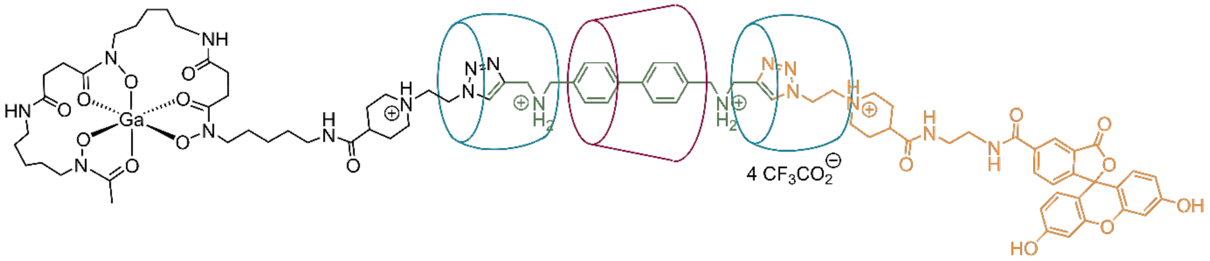

Following General procedure A, natGa-**6** was obtained as a bright yellow residue. The product was estimated by analytical HPLC to have a purity >95%; HRMS (ESI) *m/z* calcd for $C_{198}H_{254}GaN_{66}O_{75}$ $[M+5H]^{5+}$ 964.9464 found 964.9466 (100).

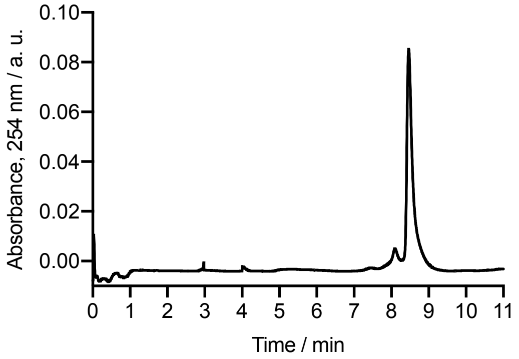

Figure S40. Reverse-phase analytical HPLC chromatogram of complex natGa-**6**, λ = 254 nm.

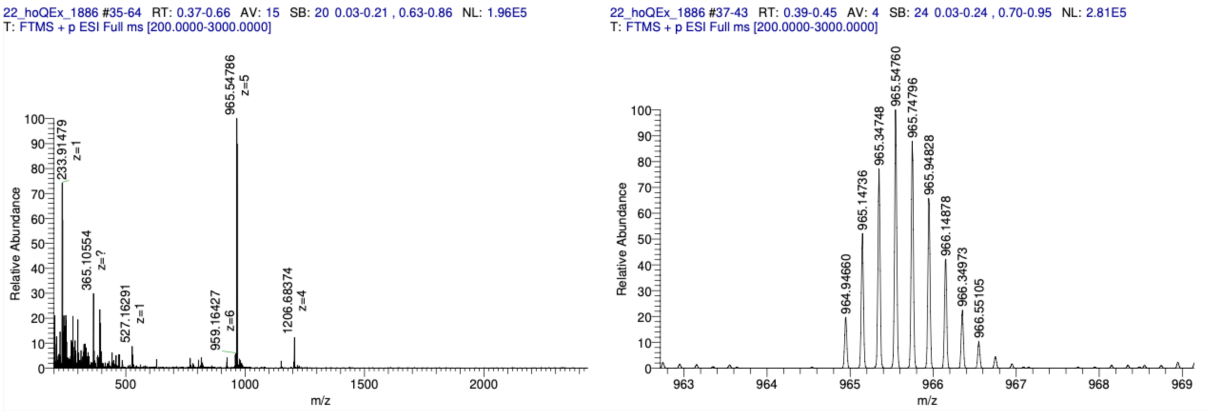

Figure S41. HRMS (ESI+) spectrum of complex natGa-**6**.

*Radiosynthesis of [^{68}Ga]Ga-**6***

Radiolabelling reactions to prepare [^{68}Ga]Ga-**6** were accomplished by the addition of an aliquot of [^{68}Ga][$Ga(H_2O)_6$]Cl_3(aq.) stock solution (~15 MBq diluted in H_2O to ~190 μL) to an aqueous solution of **6** (10 μL of 1 mM stock in H_2O) buffered with NaOAc (0.2 M, pH4.4, 50 μL) with a total reaction volume of 250 μL. The reactions were monitored by radio-iTLC (citrate buffer, 1.0 M, pH4.5) and complexation was found to be complete in less than 10 min at 23 ºC giving a radiochemical conversion (RCC) >99% (R_f = 0.0 – 0.1). The product was characterised by analytical HPLC following the method described in the general section. Note: the UV-Vis detector and radioactivity detector were arranged serially with an offset time of approximately 0.10-0.30 min (depending on temperature). The identity of the radiolabelled compound ([^{68}Ga]Ga-**6**) was confirmed by co-injection with an authenticated sample of non-radiolabelled complex ^{nat}Ga-**6**.

*Synthesis of [4]rotaxane **7***

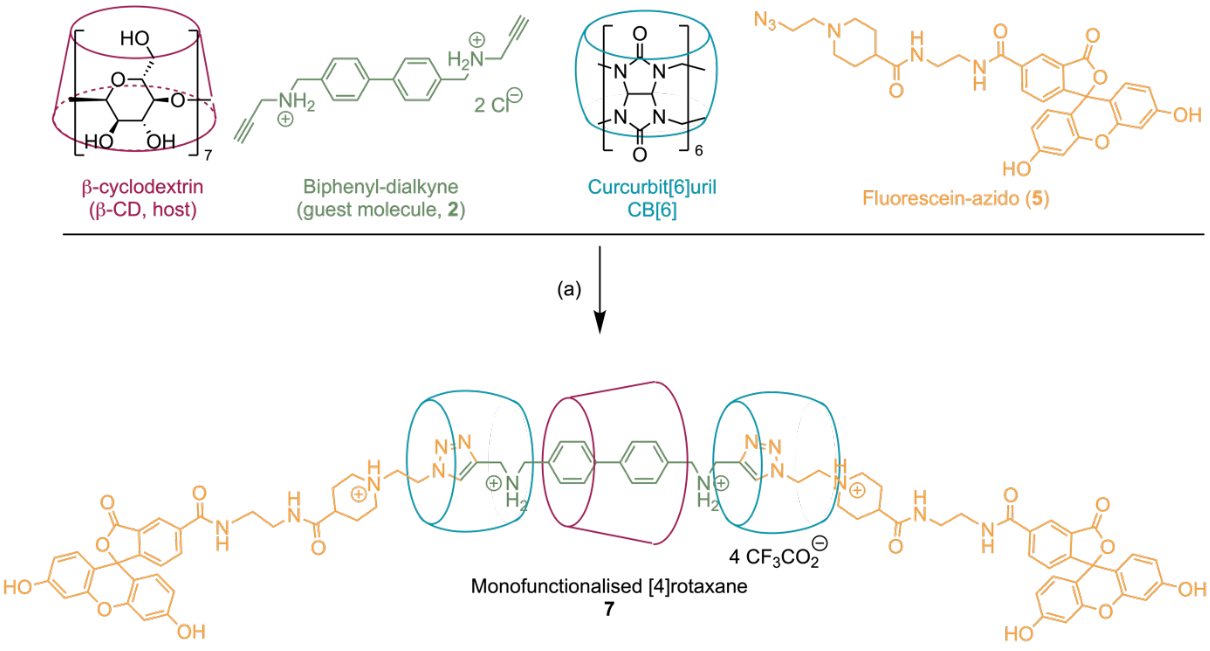

Scheme S5. Synthesis of [4]rotaxane **7**: (a) H_2O, 70 °C, 1 min, 65%.

Compound 7

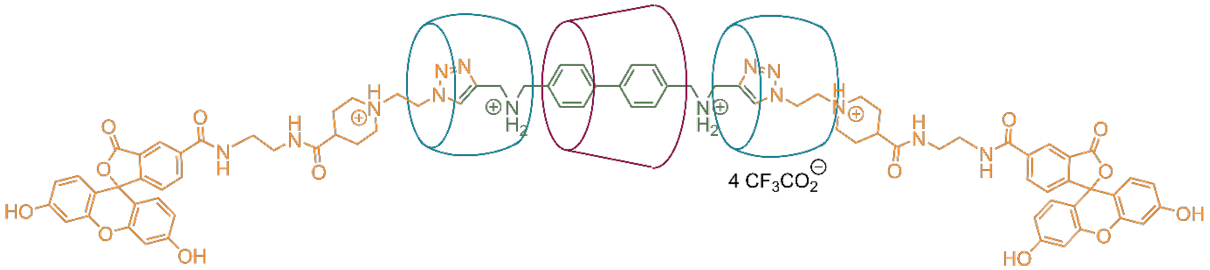

To a solution of **2** (0.35 mg, 1.2 μmol, 1 equiv.) and β-CD (1.37 mg, 1.2 μmol, 1 equiv.) in H_2O (0.5 mL) were added **5** (1.45 mg, 2.4 μmol, 2 equiv.) and CB[6] (2.41 mg, 2.4 μmol, 2 equiv.). The reaction mixture was heated to 70 °C for 1 min. The crude mixture was purified by using preparative HPLC at a flow rate of 7 mL min^{-1} with a linear gradient of A (distilled water containing 0.1% TFA) and B (MeOH, Sigma-Aldrich, HPLC grade): *t* = 0 min A 95% + B 5%, *t* = 30 min A 0% + B 100%. After lyophilisation, [4]rotaxane **7** was obtained as a bright yellow powder (3.31 mg, 65% yield). The product was estimated by analytical HPLC to have a purity >95%; ^{1}H NMR (500 MHz, D_2O) δ = 8.37 (*s*, 2H), 8.01−8.10 (*m*, 4H), 7.98 (*d*, *J* = 7.9 Hz, 2H), 7.66 (*d*, *J* = 7.9 Hz, 2H), 7.51 (*d*, *J* = 7.9 Hz, 2H), 6.83 (*s*, 4H), 6.70−6.76 (*m*, 4H), 6.54−6.61 (*m*, 4H), 6.48 (*s*, 2H), 5.53−5.79 (*m*, 24H), 5.37−5.49 (*m*, 24H), 5.00−5.07 (*m*, 7H), 4.55−4.65 (*m*, 4H), 4.36−4.46 (*m*, 4H), 4.24−4.36 (*m*, 4H), 3.97−4.24 (*m*, 28H), 3.47−3.90 (*m*, 52H), 3.02−3.18 (*m*, 4H), 2.71−2.81 (*m*, 2H), 2.12−2.27 (*m*, 4H), 1.98−2.09 ppm (*m*, 4H); $^{13}C\{^{1}H\}$ NMR (126 MHz, D_2O) δ = 176.5, 170.2, 168.4, 162.9 (*q*, ^{2}J = 35.3 Hz), 161.7, 156.3, 156.2, 156.0, 156.0, 156.0, 155.9, 153.7, 140.6, 139.9, 139.2, 139.1, 135.9, 133.9, 132.6, 131.6, 131.4, 131.1, 130.9, 130.0, 128.4, 127.6, 126.1, 125.7, 124.9, 119.6, 119.5, 116.7 (*q*, ^{1}J = 292.5 Hz), 114.4, 112.8, 111.0, 110.9, 102.8, 102.4, 81.3, 73.3, 71.9, 71.7, 70.1, 59.6, 52.7, 51.3, 51.1, 44.4, 43.3, 43.0, 39.9, 38.6, 26.4 ppm; HRMS (ESI) *m/z* calcd for $C_{196}H_{226}N_{62}O_{73}$ $[M+4H]^{4+}$ 1153.8964 found 1153.8959 (100).

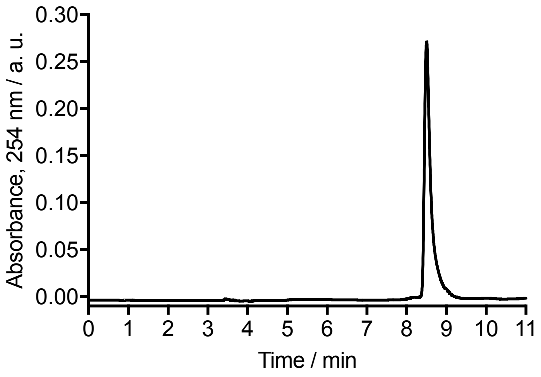

Figure S42. Reverse-phase analytical HPLC chromatogram of [4]rotaxane **7**, λ = 254 nm.

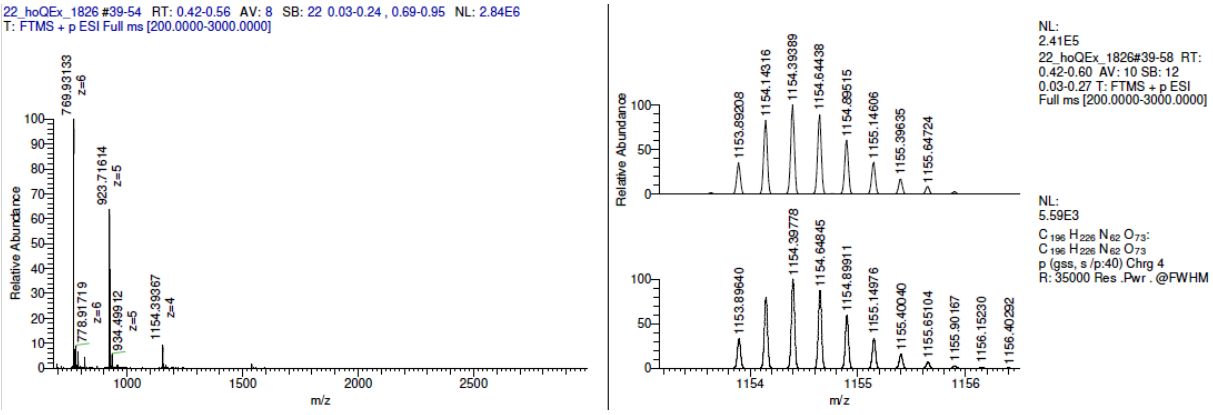

Figure S43. HRMS (ESI+) spectrum of [4]rotaxane **7**.

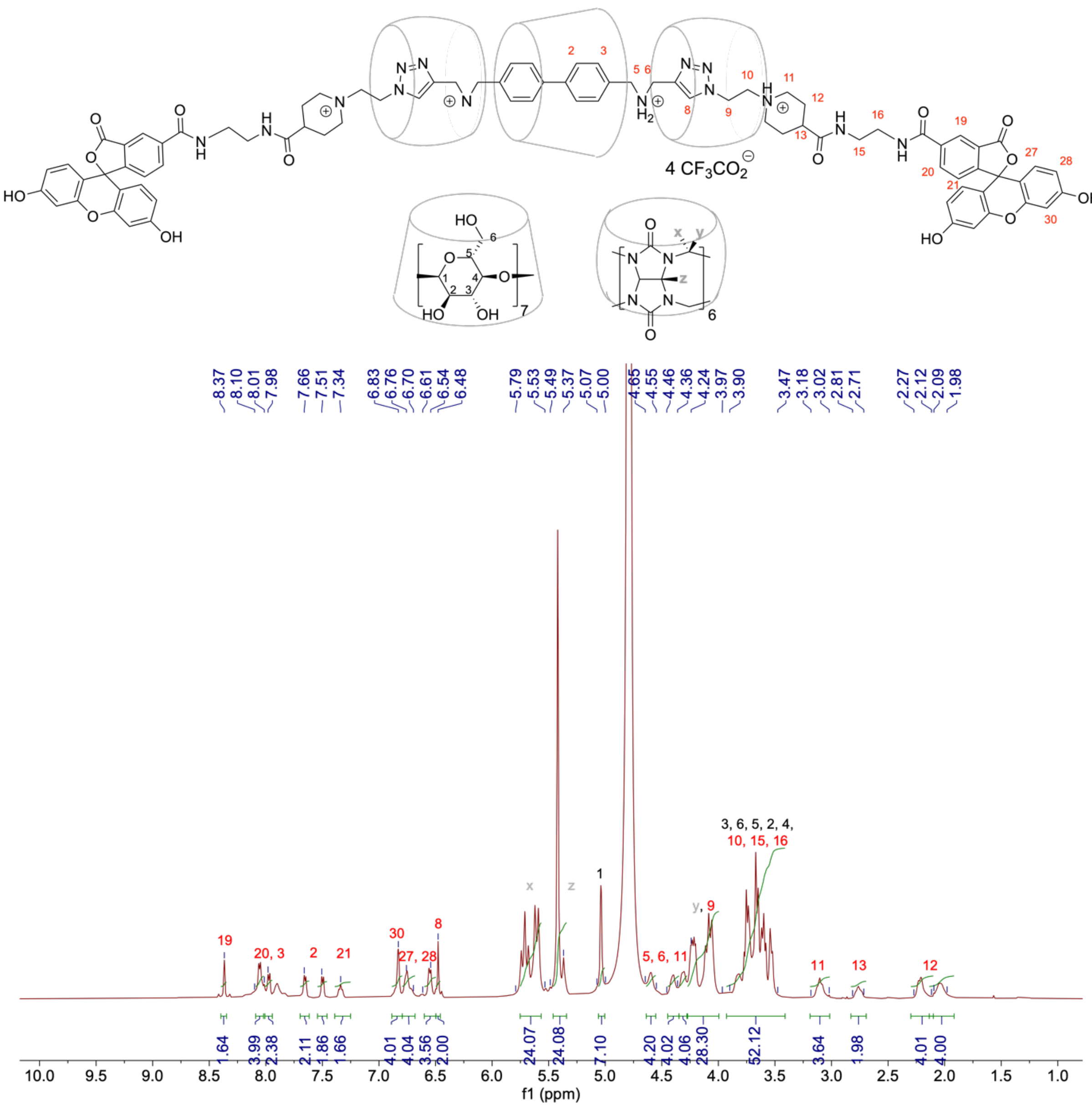

Figure S44. ^{1}H NMR of [4]rotaxane **7** (D_2O, 500 MHz).

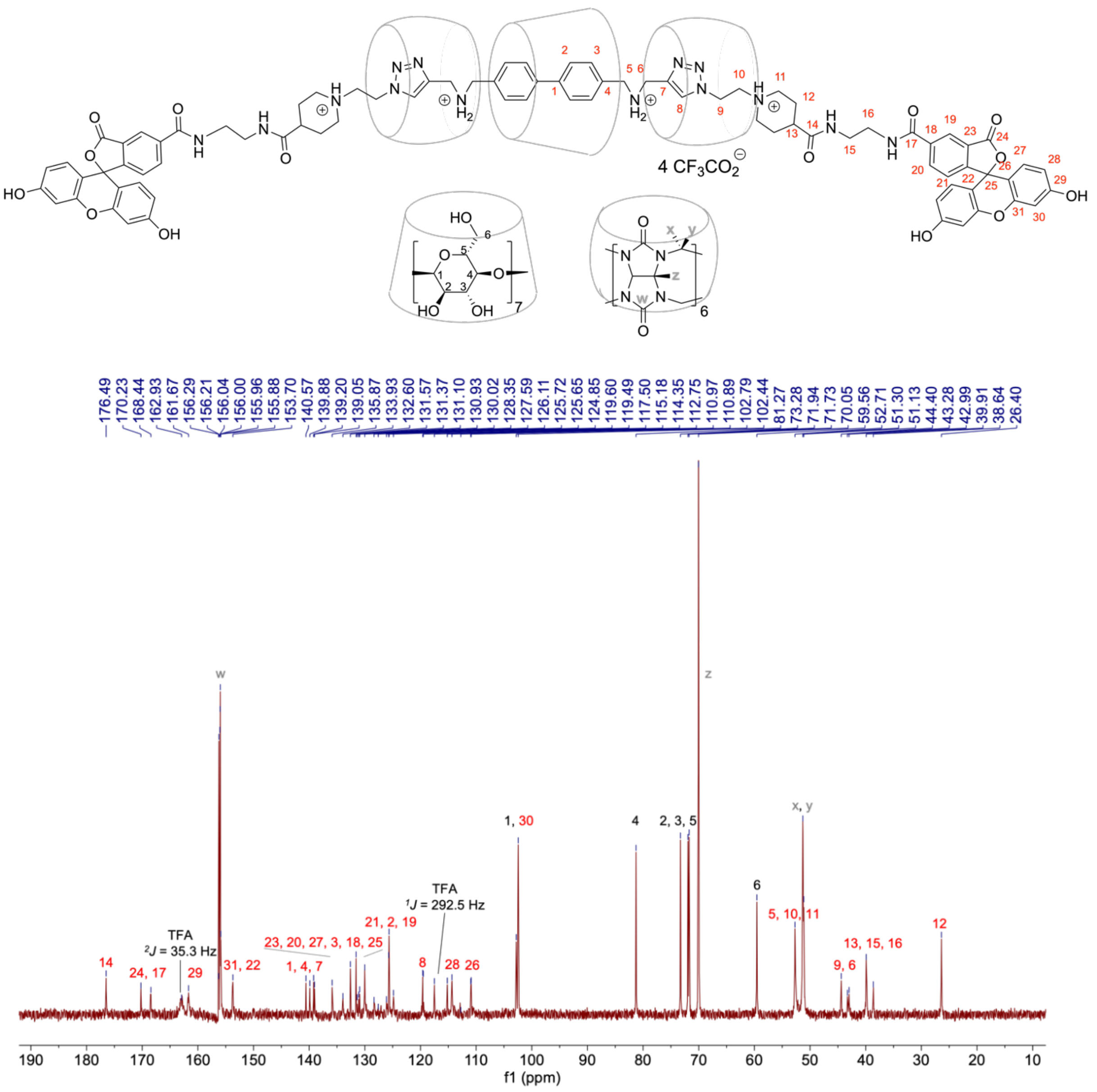

Figure S45. $^{13}C\{^{1}H\}$ NMR of [4]rotaxane **7** (D_2O, 126 MHz).

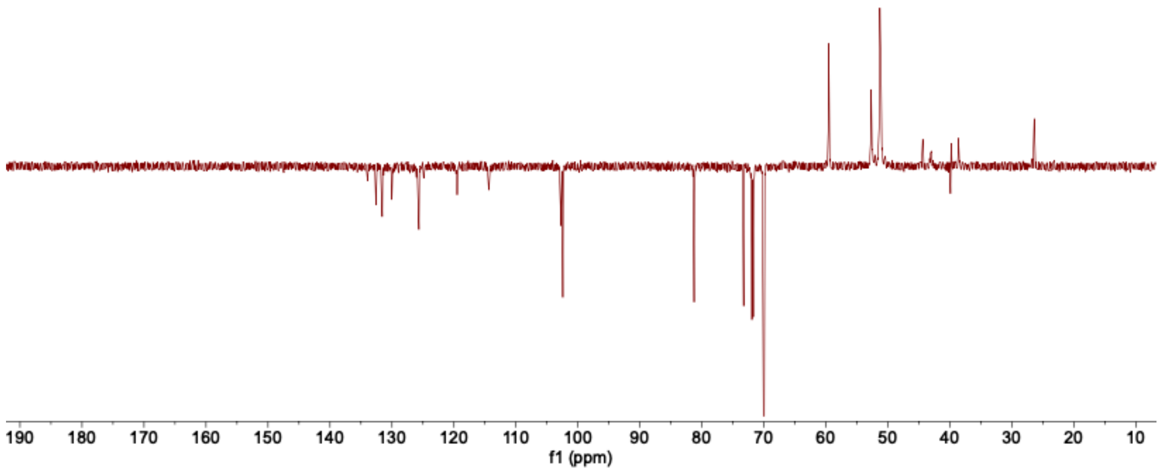

Figure S46. DEPT-135 of [4]rotaxane **7** (D_2O).

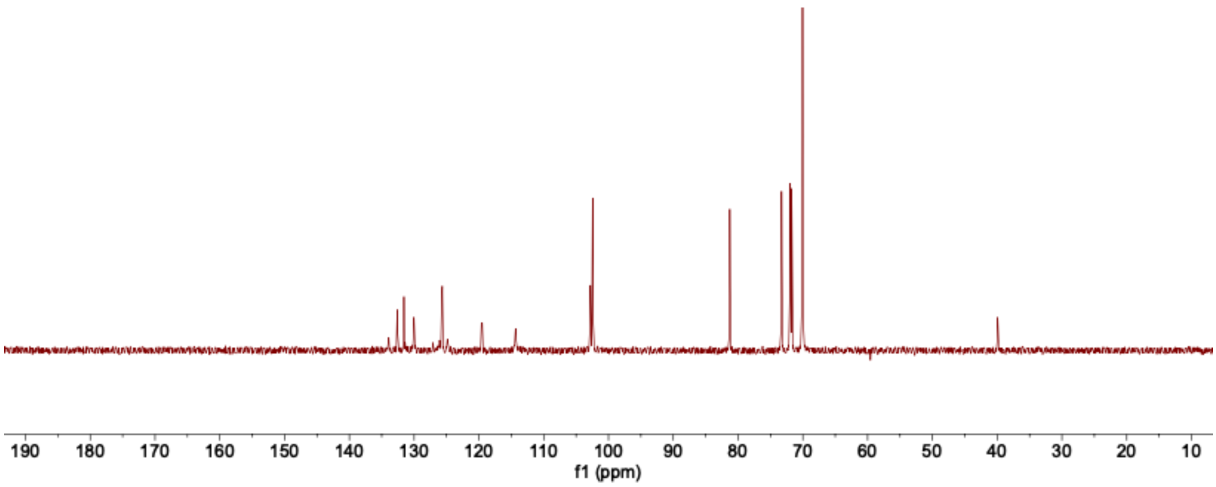

Figure S47. DEPT-90 of [4]rotaxane **7** (D_2O).

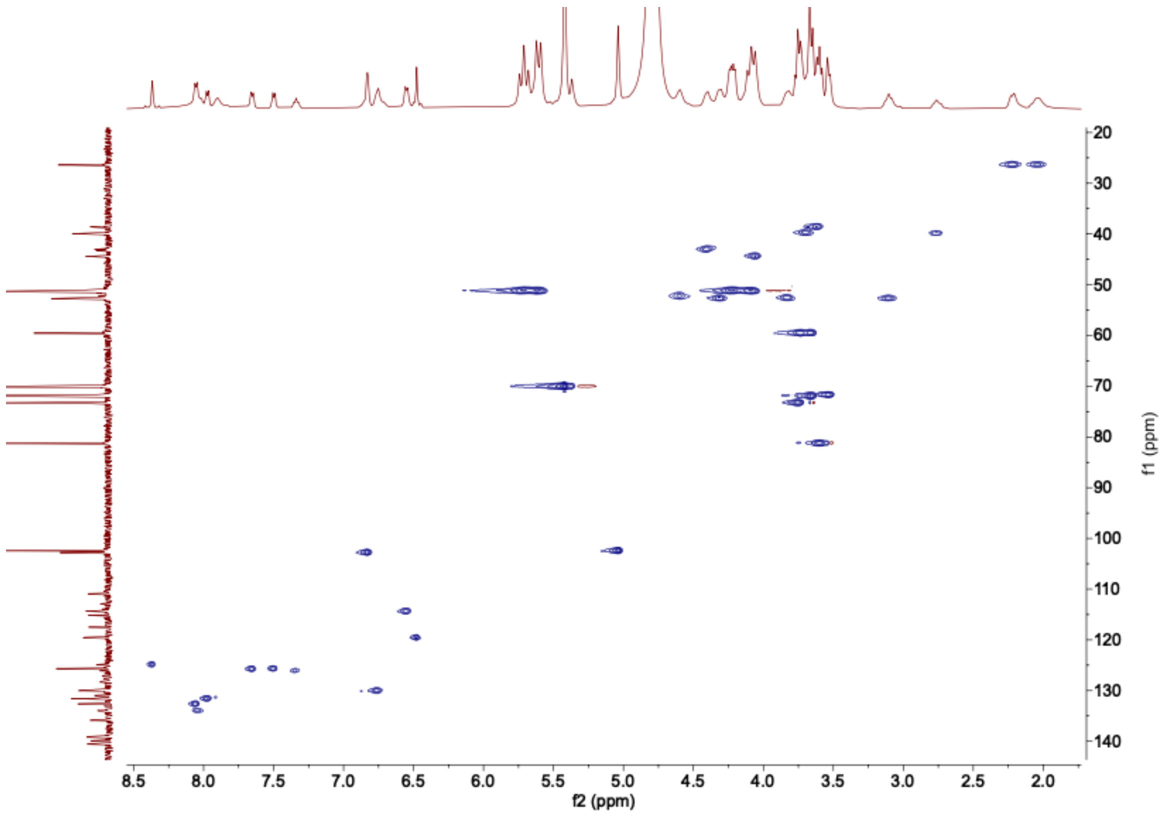

Figure S48. HSQC of [4]rotaxane **7** (D_2O).

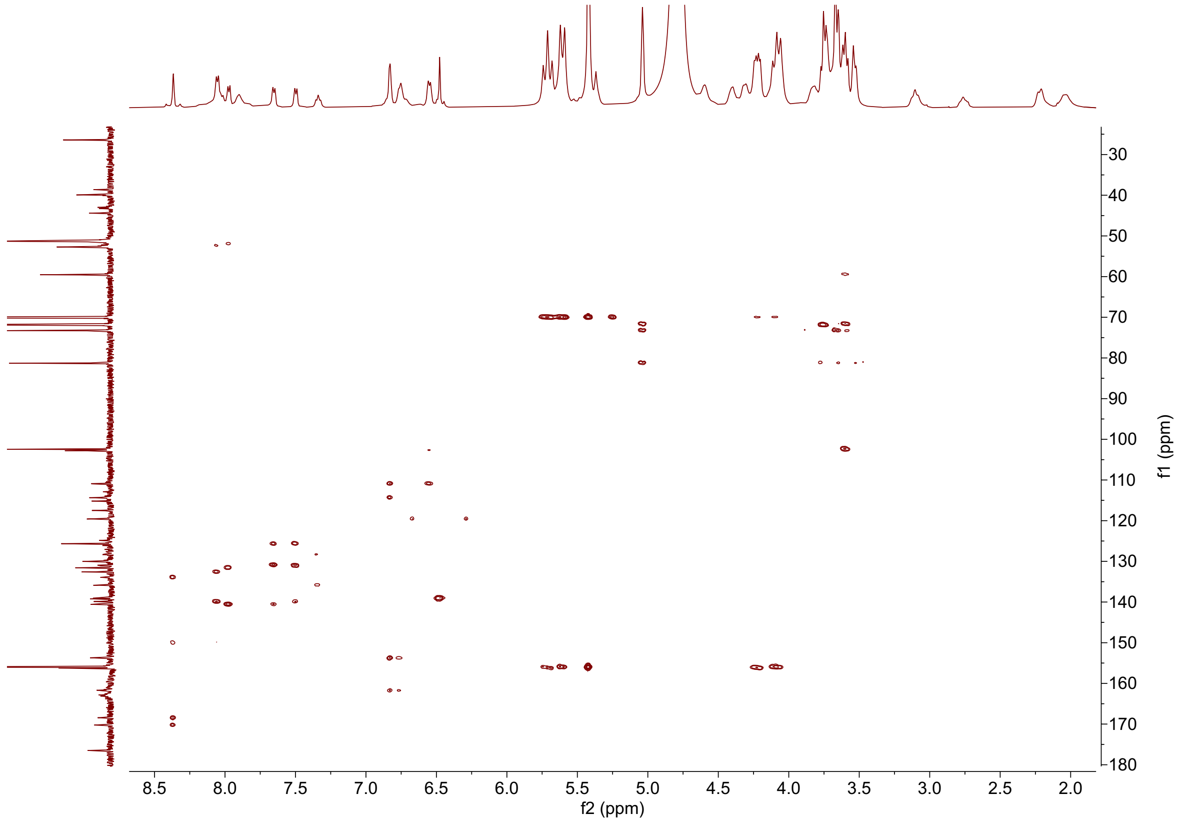

Figure S49. HMBC of [4]rotaxane **7** (D_2O).

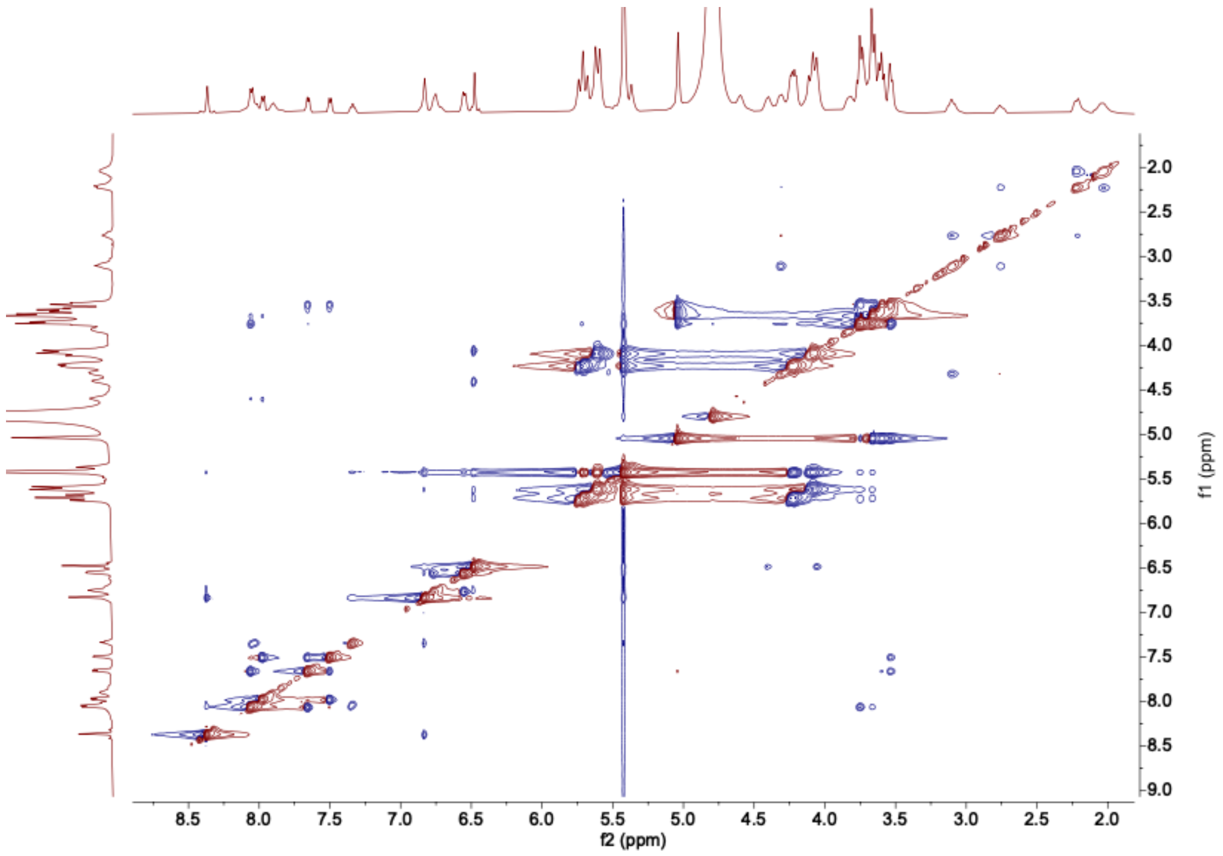

Figure S50. ROESY of [4]rotaxane **7** (D_2O).

Electronic absorption spectroscopy and determination of the molar absorption coefficient for [4]rotaxane **7**

Seven different concentrations of [4]rotaxane **7** were prepared in H_2O. The electronic absorption (UV-Vis) spectrum for each sample was recorded using a 1 cm cell. Molar absorption coefficients were calculated after plotting concentration against absorbance as shown in **Figure S51** and

Table S*1*.

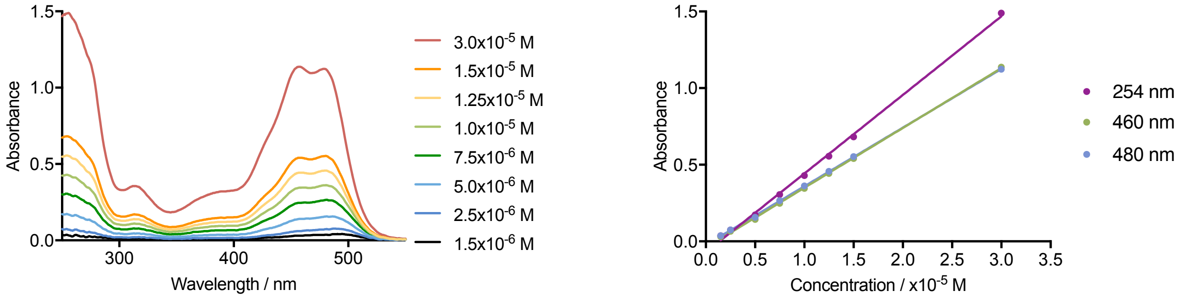

Figure S51. Electronic absorption spectroscopy to determine molar absorption coefficients of [4]rotaxane **7**.

Table S1. Molar absorption coefficients of [4]rotaxane **7**.

Wavelength / nm	254	460	480
Molar absorption coefficient ε / $M^{-1}.cm^{-1}$	51,280	39,030	38,260

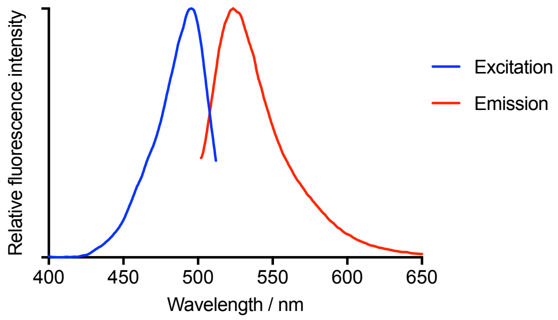

Figure S52. Excitation and fluorescence emission spectra for the [4]rotaxane **7**.

*Synthesis of the PSMA targeted [3]semirotaxane **9** and metallo[3]rotaxane ^{nat}Ga-**9***

Compound 9

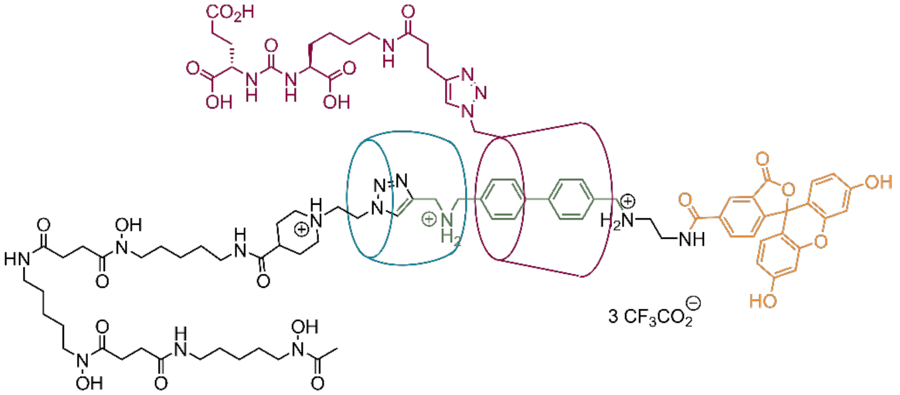

To a solution of **1** (1 mg, 1.5 µmol, 1 equiv.) and **8** (2.4 mg, 1.5 µmol, 1 equiv.) in H_2O (0.5 mL) were added **3** (1.1 mg, 1.5 µmol, 1 equiv.) and CB[6] (1.5 mg, 1.5 µmol, 1 equiv.). The reaction mixture was heated to 70 °C for 1 min. The crude was purified by using preparative HPLC at a flow rate of 7 mL min^{-1} with a linear gradient of A (MeOH, Sigma-Aldrich, HPLC grade) and B (distilled water containing 0.1% TFA): *t* = 0 min A 5% + B 95%, *t* = 30 min A 100% + B 0%. After lyophilisation, **9** was obtained as a white powder (2.3 mg, 38% yield). The product was estimated by analytical HPLC to have a purity >90%; ^{1}H NMR (500 MHz, D_2O) δ = 8.22−8.33 (*m*, 1H), 8.06 (*d*, *J* = 8.0 Hz, 2H), 7.96−8.05 (*m*, 1H), 7.60−7.68 (*m*, 4H), 7.50 (*d*, *J* = 8.0 Hz, 2H), 7.42 (*s*, 1H), 7.26−7.33 (*m*, 1H), 6.91−7.07 (*m*, 3H), 6.76−6.91 (*m*, 2H), 6.56 (*s*, 1H), 5.67−5.81 (*m*, 12H), 5.50 (*s*, 12H), 4.94−5.08 (*m*, 7H), 4.36−4.65 (*m*, 6H), 4.23−4.36 (*m*, 14H), 4.13−4.23 (*m*, 2H), 3.94−4.13 (*m*, 2H), 3.40−3.94 (*m*, 50H), 3.09−3.29 (*m*, 12H), 3.01−3.09 (*m*, 2H), 2.74−2.85 (*m*, 5H), 2.40−2.58 (*m*, 10H), 2.18−2.29 (*m*, 2H), 2.13 (*s*, 3H), 2.03−2.18 (*m*, 2H), 1.75−1.91 (*m*, 2H), 1.55−1.75 (*m*, 8H), 1.44−1.55 (*m*, 8H), 1.24−1.42 ppm (*m*, 6H); ^{13}C{^{1}H} NMR (126 MHz, D_2O) δ = 177.2, 176.4, 176.0, 175.7, 174.8, 174.1, 173.8, 173.6, 170.5, 168.8, 163.6, 163.0 (*q*, ^{2}J = 36.1 Hz), 159.1, 156.3, 156.2, 156.1, 154.8, 146.2, 141.4, 139.9, 139.1, 134.9, 132.6, 131.0, 130.1, 126.4, 126.0, 124.0, 119.6, 116.3 (*q*, ^{1}J = 293.2 Hz), 115.7, 112.0, 111.9, 103.0, 102.5, 102.3, 81.7, 73.4, 73.3, 73.2, 73.2, 73.0, 71.9, 71.8, 71.7, 71.4, 70.3, 70.2, 70.1, 59.9, 58.4, 54.9, 53.3, 52.8, 52.0, 51.5, 51.4, 47.8, 47.7, 44.5, 42.7, 40.0, 39.9, 39.2, 39.1, 34.5, 30.7, 30.4, 30.1, 29.4, 27.9, 27.8, 27.6, 26.4, 26.2, 26.1, 25.8, 25.4, 23.0, 22.3, 20.9, 19.6, 19.2 ppm; HRMS (ESI) *m/z* calcd for $C_{168}H_{227}N_{43}O_{69}$ $[M+4H]^{4+}$ 987.6388 found 987.6393 (100).

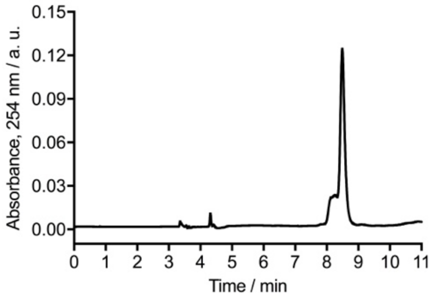

Figure S53. Reverse-phase analytical HPLC chromatogram of [3]semirotaxane **9**, λ= 254 nm.

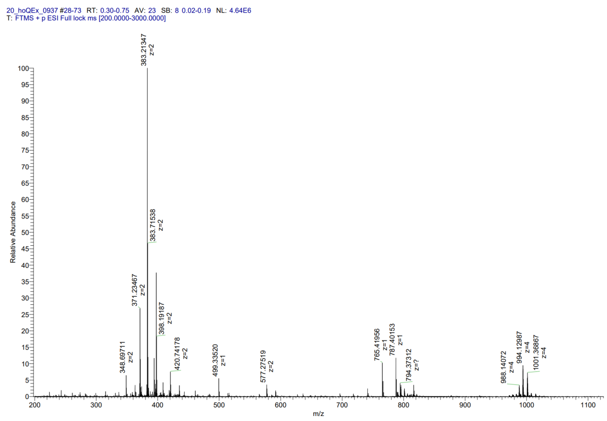

Figure S54. HRMS (ESI+) spectrum of [3]semirotaxane **9**.

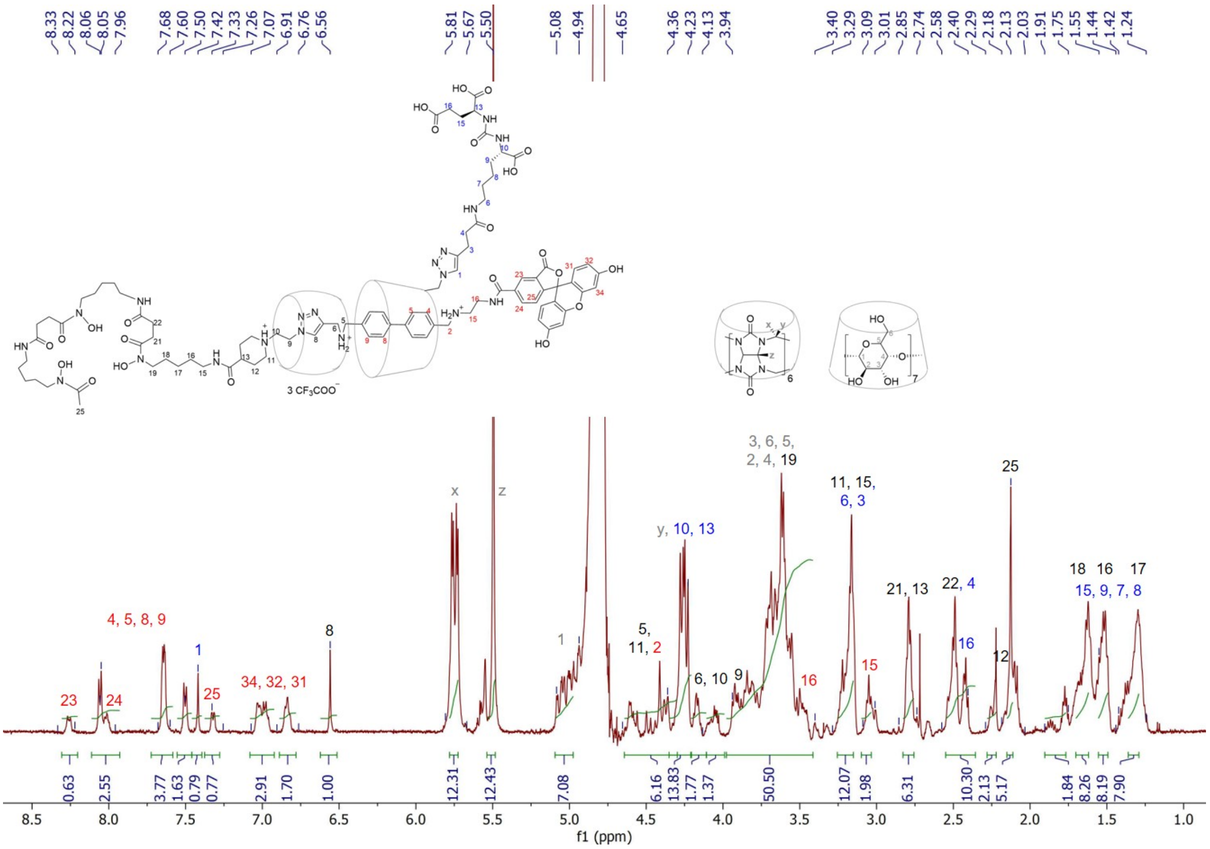

Figure S55. ^{1}H NMR of [3]semirotaxane **9** (D_2O, 500 MHz).

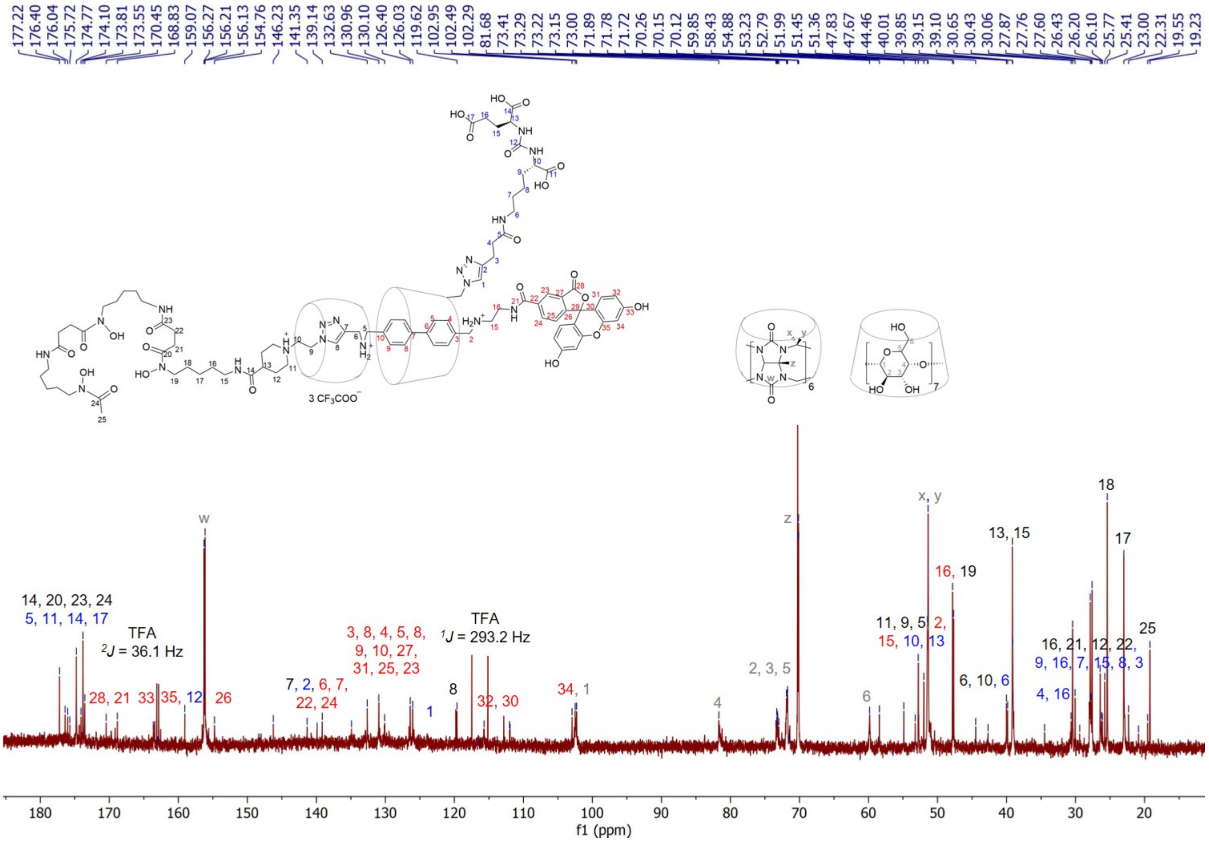

Figure S56. $^{13}C\{^{1}H\}$ NMR of [3]semirotaxane **9** (D_2O, 126 MHz).

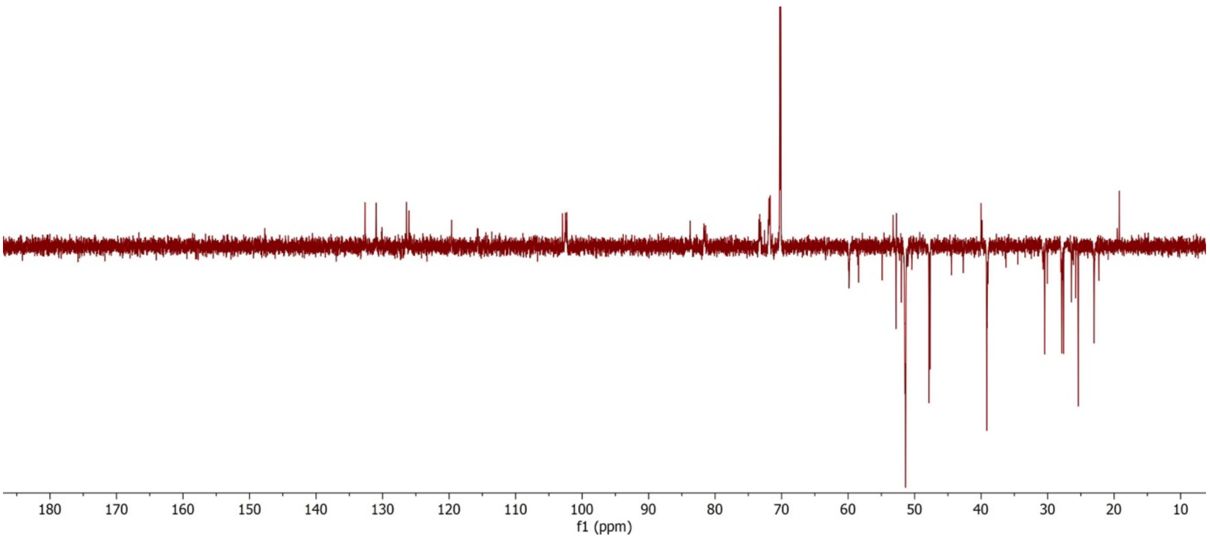

Figure S57. DEPT-135 of [3]semirotaxane **9** (D_2O).

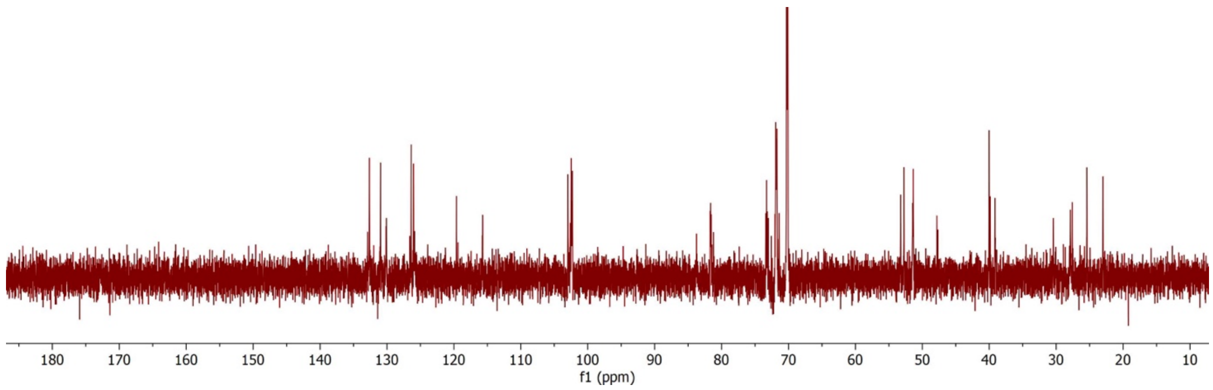

Figure S58. DEPT-90 of [3]semirotaxane **9** (D_2O).

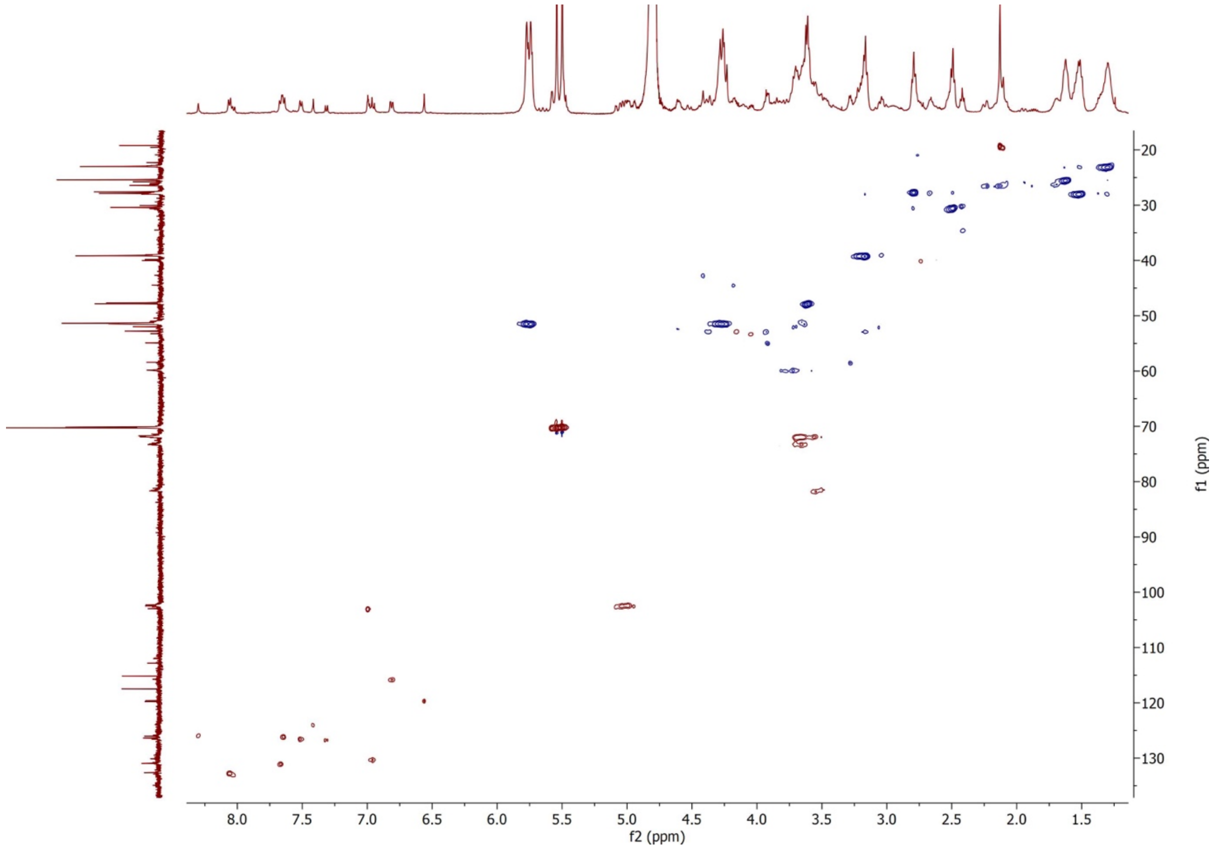

Figure S59. HSQC of [3]semirotaxane **9** (D_2O).

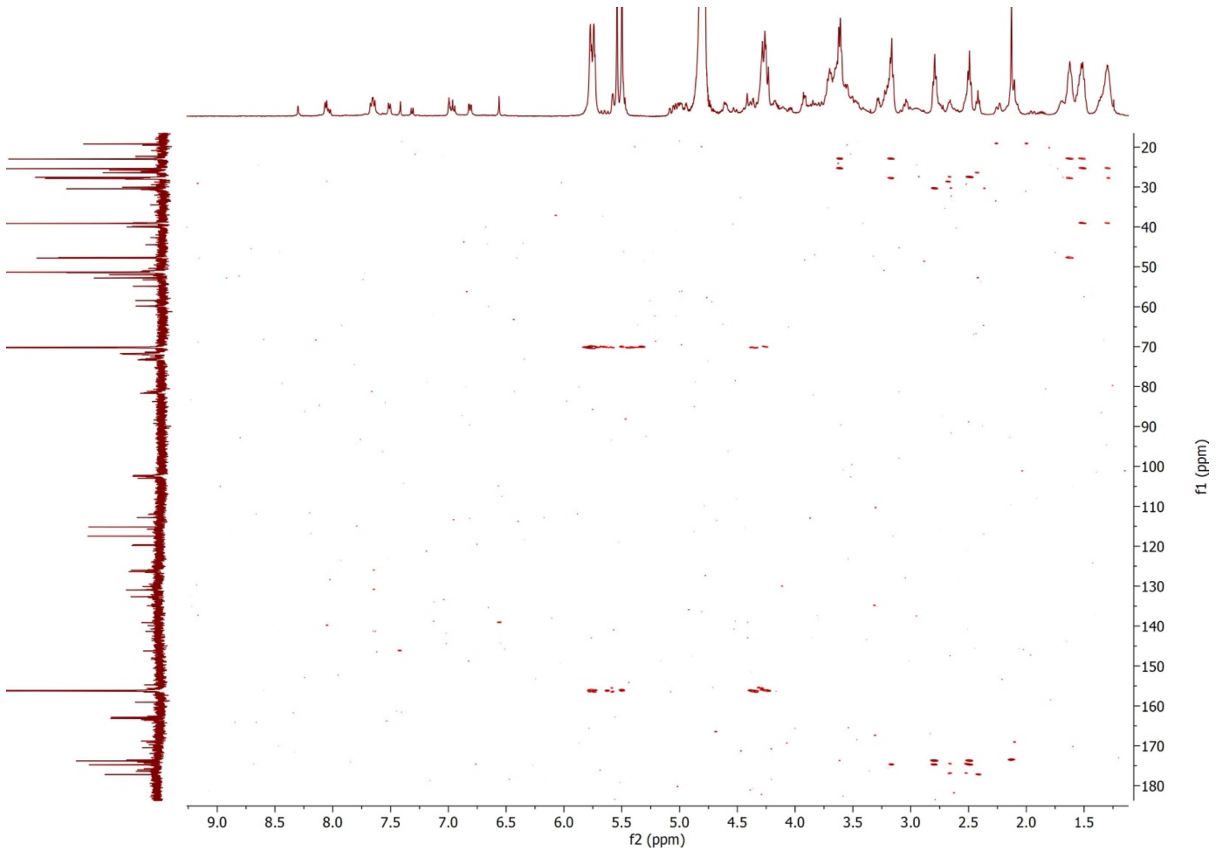

Figure S60. HMBC of [3]semirotaxane **9** (D_2O).

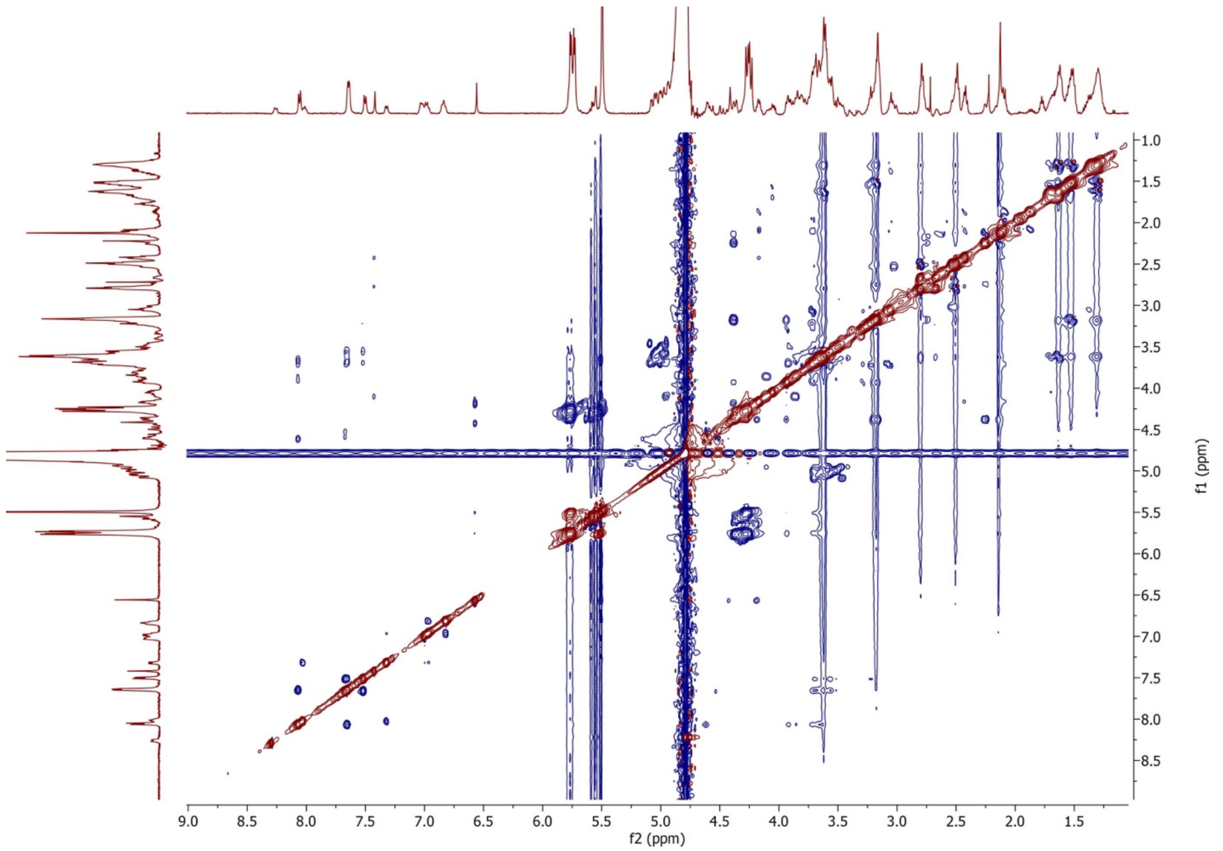

Figure S61. ROESY of [3]semirotaxane **9** (D_2O).

Metallo[3]rotaxane ^{nat}Ga-9

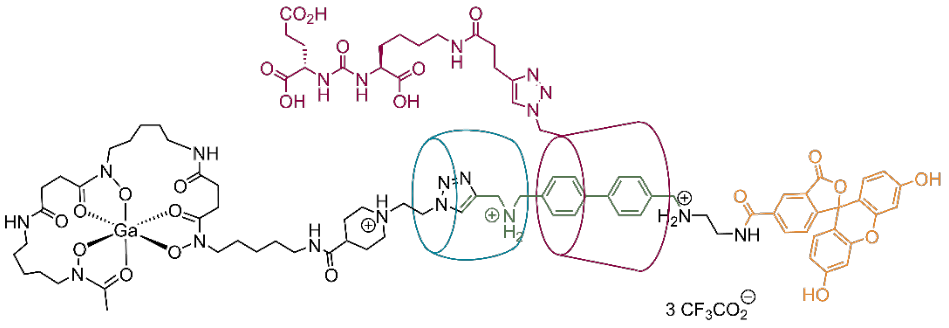

Following General procedure A, ^{nat}Ga-**9** was obtained as a slightly yellow residue. The product was estimated by analytical HPLC to have a purity >95%; HRMS (ESI) *m/z* calcd for $C_{168}H_{226}GaN_{43}O_{69}$ $[M+4H]^{4+}$ 1004.6183 found 1004.6175 (100).

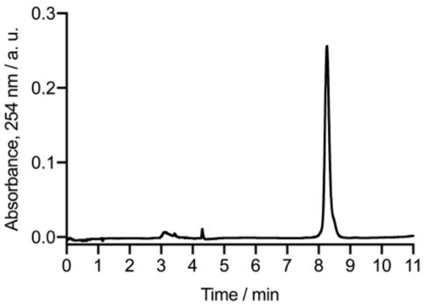

Figure S62. Reverse-phase analytical HPLC chromatogram of complex ^{nat}Ga-**9**, λ= 254 nm.

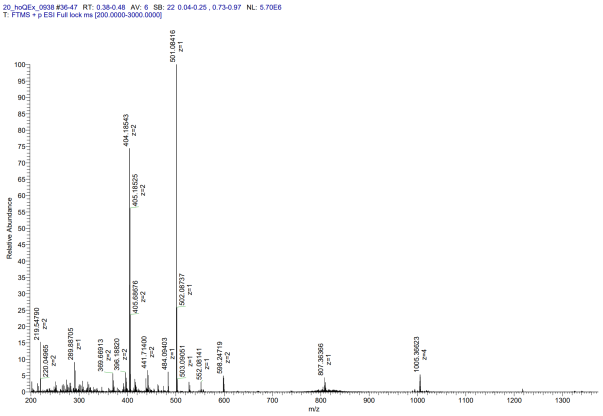

Figure S63. HRMS (ESI+) spectrum of complex ^{nat}Ga-**9**.

*Radiosynthesis of [^{68}Ga]Ga-**9***

Radiolabelling reactions to prepare [^{68}Ga]Ga-**9** were accomplished by the addition of an aliquot of [^{68}Ga][Ga(H_2O)$_6$]Cl_3(aq.) stock solution (~14 MBq diluted in H_2O to ~190 μL) to an aqueous solution of **9** (10 μL of 1 mM stock in H_2O) buffered with NaOAc (0.2 M, pH4.4, 50 μL) with a total reaction volume of 250 μL. The reactions were monitored by radio-iTLC (citrate buffer, 1.0 M, pH4.5) and complexation was found to be complete in less than 10 min at 23 °C giving a radiochemical conversion (RCC) >99% (R_f = 0.0 – 0.1). The product was characterised by analytical HPLC following the method described in the general section. Note: the UV-Vis detector and radioactivity detector were arranged serially with an offset time of approximately 0.10-0.30 min (depending on temperature). After optimisation [^{68}Ga]Ga-**9** was isolated with a molar activity of 17 MBq nmol^{-1} (measured with titration experiments, R^2 = 0.9892). The identity of the radiolabelled compound ([^{68}Ga]Ga-**9**) was confirmed by co-injection with an authenticated sample of non-radiolabelled complex ^{nat}Ga-**9**.

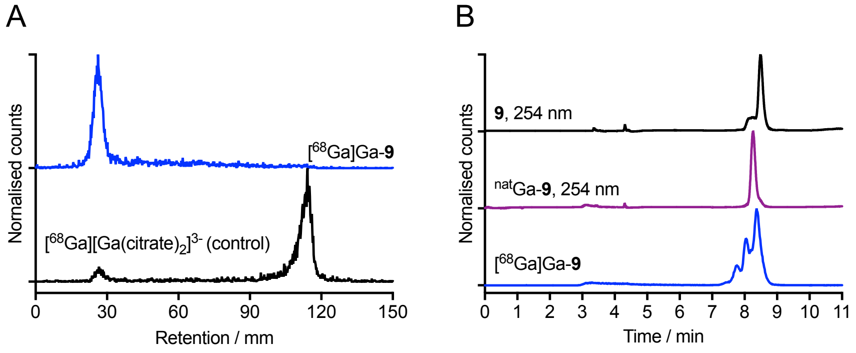

Figure S64. (A) Radio-iTLC chromatograms of **9** in citrate buffer and associated control; (B) Analytical HPLC chromatograms recorded at 254 nm of the purified semirotaxane **9**, and the corresponding ^{nat}Ga-**9** and [^{68}Ga]Ga-**9** [3]rotaxanes.

*Quantum yield of compound ^{nat}Ga-**9***

The fluorescence emission quantum yield of compound ^{nat}Ga-**9** was determined with respect to fluorescein in 0.1 M NaOH.[2] Compounds were excited at 480 nm and the integral of the emission from 485-620 nm was taken.

Equation S1. Quantum yield calculated using the comparative method of Williams et al.,[2] which involves the use of the well characterised standard fluorescein sample with a known quantum yield $\Phi_{\text{Fluorescein}}$.

$$\Phi_{\text{GaFD-301}} = \Phi_{\text{Fluorescein}} \frac{\text{Slope(natGa}-\mathbf{9})}{\text{Slope(fluorescein)}}$$

*Electronic absorption spectroscopy and determination of the molar absorption coefficient for compound ^{nat}Ga-**9***

Seven different concentrations of compound ^{nat}Ga-**9** were prepared in H_2O. The electronic absorption (UV-Vis) spectrum for each sample was recorded using a 1 cm cell. Molar absorption coefficients and were extracted by plotting concentration against absorbance as shown in **Figure S65** and **Table S2**.

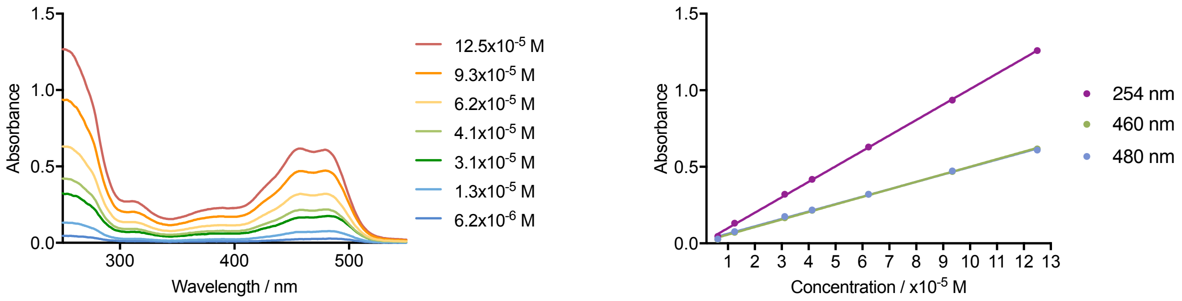

Figure S65. Electronic absorption spectroscopy to determine molar absorption coefficients of compound ^{nat}Ga-**9**.

Table S2. Molar absorption coefficients of compound ^{nat}Ga-**9**.

Wavelength / nm	254	460	480
Molar absorption coefficient ε / $M^{-1}.cm^{-1}$	10,091	4.926	4,836

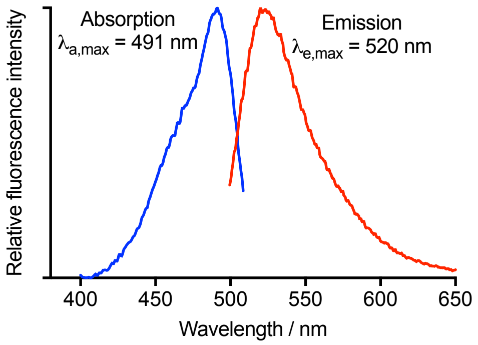

Figure S66. Electronic excitation and fluorescence emission spectra of compound ^{nat}Ga-**9**.

*Stability studies for **4**, **9**, ^{nat}Ga-**4**, ^{nat}Ga-**9**, [^{68}Ga]Ga-**4**and [^{68}Ga]Ga-**9***

Stability in water

Fresh solutions of **4**, **9**, ^{nat}Ga-**4**, ^{nat}Ga-**9** were prepared in H_2O (0.4 mM) and left at 23 °C for 96 h.

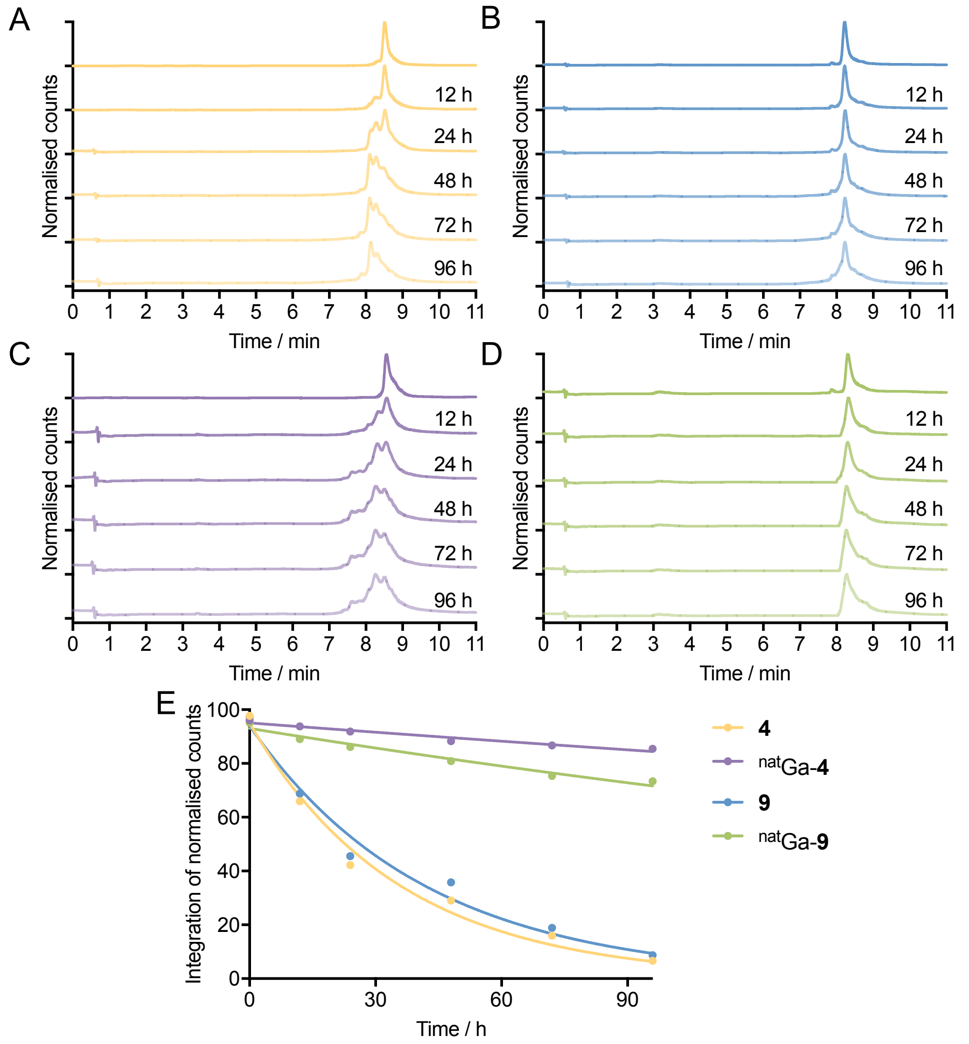

Figure S67. HPLC chromatograms recorded at 254 nm at various time points for: (A) **4**, (B) ^{nat}Ga-**4**, (C) **9**, (D) ^{nat}Ga-**9**, and (E) the corresponding plot obtained from integration of the HPLC data showing the relative stability of the compounds *versus* time.

Stability in PBS

[^{68}Ga]Ga-**4** and [^{68}Ga]Ga-**9** were prepared as previously described. The pH was adjusted to 8.0-8.5 with 1 M Na_2CO_3(aq.). To 100 µL of the reaction, 100 µL of PBS was added. The samples were incubated at 37 °C and the radiochemical purity (RCP) was monitored via radio-TLC at time points up to 2 h.

Table S3. Percentage RCP of [^{68}Ga]Ga-**4** and [^{68}Ga]Ga-**9** determined from radio-iTLC following incubation with PBS up to 2 h at 37 °C.

Time / min	RCP ± S.D. / %	
	[^{68}Ga]Ga-**4**	[^{68}Ga]Ga-**9**
0	97.0	99.0
10	97.7 ± 1.5	97.7 ± 2.1
30	95.3 ± 2.3	96.3 ± 1.0
60	95.0 ± 2.0	95.3 ± 1.5
120	94.3 ± 1.5	97.0 ± 0.5

Stability in human serum albumin

Reactions were prepared as described previously, human serum albumin (200 µL) was added, and the samples were incubated at 37 °C. Stability in human serum was monitored *via* radio-SEC (mobile phase: PBS) for time points up to 2 h.

Table S4. Percentage RCP of [^{68}Ga]Ga-**4** and [^{68}Ga]Ga-**9** determined from radio-SEC-HPLC following incubation with human serum for up to 2 h at 37 °C.

Time / min	RCP / %	
	[^{68}Ga]Ga-**4**	[^{68}Ga]Ga-**9**
0	96	100
10	93	95
60	96	96
120	94	92

*Cell binding assays with [^{68}Ga]Ga-**4** and [^{68}Ga]Ga-**9***

Cells were harvested and distributed in Eppendorf tubes (2.5 x 10^6 cells / vial) in media (270 µL) or media with sodium azide (270 µL, 0.1%). Reactions were prepared as previously described and diluted (3-fold) in cell media and then added (30 µL, 100 kBq) to the prepared cells. After mixing for 1.5 h at 37 °C, the samples were centrifuged (2000 rpm, 3 min) and the cell pellet washed with ice-cold PBS (2 x 1 mL) keeping the samples on ice between washes. The radioactivity associated with each sample was quantified by using the gamma counter. Experiments were performed in triplicate. Aliquots of the reactions (30 µL, 100 kBq) were added into three additional Eppendorf tubes not containing cells and used as control to measure the total activity.

Blocking Assay

Cells were harvested and distributed in Eppendorf tubes (2.5 $\times$ 10^6 cells / vial) in media with PSMA binding motif (200 µM). Cells were then incubated for 1 h. Reactions were prepared as described above, diluted in cell media (3-fold) and added (30 µL, 100 kBq) to the prepared cells. After mixing for 1.5 h at 37 °C, the samples were centrifuged (2000 rpm, 3 min) and the cell pellet washed with ice-cold PBS (2 $\times$ 1 mL) keeping the samples on ice between washes. The radioactivity associated with each sample was quantified by using the gamma counter. Experiments were performed in triplicate.

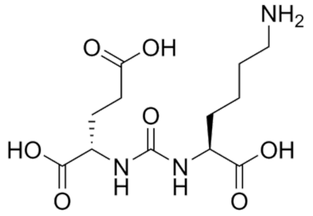

Figure S68. Chemical structure of the PSMA binding ligand used in cellular blocking experiments.

References

1. d'Orchymont, F. & Holland, J. P. Supramolecular Rotaxane-Based Multi-Modal Probes for Cancer Biomarker Imaging**. *Angew. Chemie - Int. Ed.* **61**, e202204072 (2022).
2. Rhys Williams, A. T., Winfield, S. A. & Miller, J. N. Relative fluorescence quantum yields using a Computer-controlled luminescence spectrometer. *Analyst* **108**, 1067–1071 (1983).
